# 1-Deazaguanosine-Modified RNA: The Missing Piece for Functional RNA Atomic Mutagenesis

**DOI:** 10.1021/jacs.2c01877

**Published:** 2022-06-06

**Authors:** Raphael Bereiter, Eva Renard, Kathrin Breuker, Christoph Kreutz, Eric Ennifar, Ronald Micura

**Affiliations:** † Institute of Organic Chemistry and Center for Molecular Biosciences, University of Innsbruck, Innrain 80-82, Innsbruck 6020, Austria; ‡ Architecture et Réactivité de l’ARN - CNRS UPR 9002, Université de Strasbourg, Institut de Biologie Moléculaire et Cellulaire, 2 Allée Conrad Roentgen, Strasbourg 67084, France

## Abstract

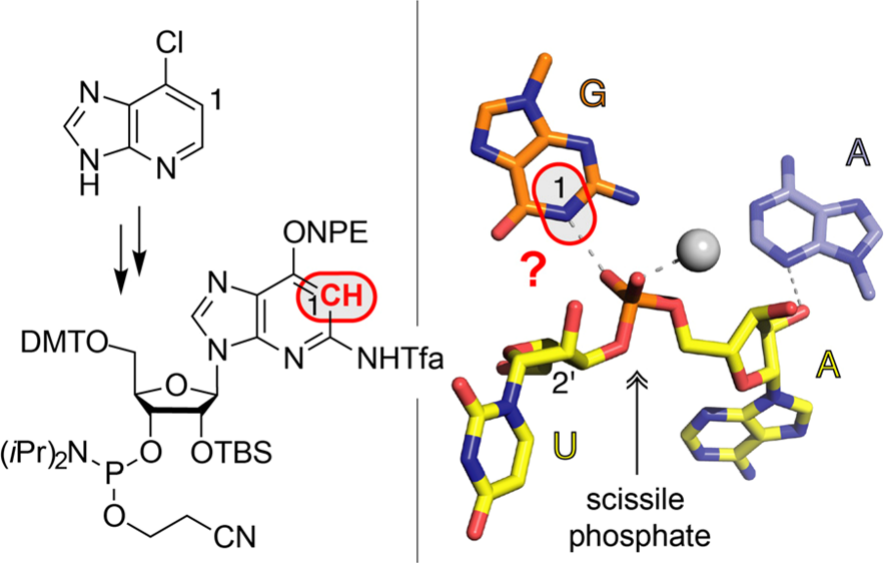

Atomic mutagenesis is the key to advance our understanding of RNA recognition and RNA catalysis. To this end, deazanucleosides are utilized to evaluate the participation of specific atoms in these processes. One of the remaining challenges is access to RNA-containing 1-deazaguanosine (c^1^G). Here, we present the synthesis of this nucleoside and its phosphoramidite, allowing first time access to c^1^G-modified RNA. Thermodynamic analyses revealed the base pairing parameters for c^1^G-modified RNA. Furthermore, by NMR spectroscopy, a c^1^G-triggered switch of Watson-Crick into Hoogsteen pairing in HIV-2 TAR RNA was identified. Additionally, using X-ray structure analysis, a guanine–phosphate backbone interaction affecting RNA fold stability was characterized, and finally, the critical impact of an active-site guanine in twister ribozyme on the phosphodiester cleavage was revealed. Taken together, our study lays the synthetic basis for c^1^G-modified RNA and demonstrates the power of the completed deazanucleoside toolbox for RNA atomic mutagenesis needed to achieve in-depth understanding of RNA recognition and catalysis.

## Introduction

Deazanucleosides are needed for atomic mutagenesis studies to explore RNA structure, function, and catalysis.^1−5^ The exchange of a specific nitrogen atom in a nucleobase by carbon can critically affect RNA properties because the hydrogen acceptor (imino, =N−) or hydrogen donor (amido or amino, −NH−) capabilities are impaired at the specific position.^6−8^ This is crucial for base pairing,^8,9^ RNA–protein recognition,^3,6,8,9^ RNA–small molecule recognition,^10^ and RNA-catalyzed chemical reactions.^4,11−14^ In particular, atomic mutagenesis lead to our current mechanistic understanding of ribozymes,^15−18^ including the ribosome.^19−22^

Thus far, diverse deazanucleosides have been utilized for RNA atomic mutagenesis experiments; these are 3-deazacytidine (c^3^C),^14,23,24^ 7-deazaadenosine (c^7^A),^4,12−14,16,17^ 3-deazaadenosine (c^3^A),^15,23^ 1-deazaadenosine (c^1^A),^12,13,15,23^ and 7-deazaguanosine (c^7^G).^14,16^ Furthermore, an efficient synthesis of 3-deazaguanosine (c^3^G) and the corresponding phosphoramidite has been reported recently and adds to the deazanucleoside tool box.^25,26^ The missing piece, however, is 1-deazaguanosine (c^1^G), which is urgently needed for RNA atomic mutagenesis studies to probe the role of active site guanosines in catalysis of diverse ribozymes and for ligand recognition in the binding pockets of many riboswitches. In this work, we present a novel synthetic route toward c^1^G, the corresponding phosphoramidite and its incorporation into oligoribonucleotides by RNA solid-phase synthesis. Furthermore, we describe the impact of c^1^G on the thermodynamic stability of RNA double helices. Moreover, we found evidence for Hoogsteen base pair formation of c^1^G with protonated cytosine in HIV-2 TAR RNA by nuclear magnetic resonance (NMR) spectroscopy. The study is complemented by the crystal structure of a c^1^G-containing RNA hairpin to shed light on a specific guanine N1–phosphate backbone interaction observed in the wild-type RNA, and finally, we evaluate the crucial role of the guanosine N1 atom in catalysis of phosphodiester cleavage by the twister ribozyme.

## Results and Discussion

To date, synthetic routes to 1-deazaguanine nucleoside building blocks for oligonucleotide synthesis have been described for DNA only.^27^ DNA containing 1-deaza-2′-deoxyguanosine (c^1^dG) is unstable toward acids, and this feature has been utilized to generate abasic sites.^28^ Access to the naked ribonucleoside 1-deazaguanosine was first reported in the nineteen eighties,^29,30^ employing rather harsh nucleosidation reactions involving mercury cyanide and based on *O*^6^-benzylated 1-deazaguanine, which itself requires laborious multistep synthesis.^31^ Later, access to 1-deazaguanosine was demonstrated via 5-amino-1-β-d-ribofuranosylimidazole-4-carboxamide (AICA-riboside) as the key intermediate.^32^ We, however, decided to put efforts into a direct, more efficient, and unprecedented route from readily available starting materials.

### Synthesis of c^1^G Nucleoside

**Scheme 1:**
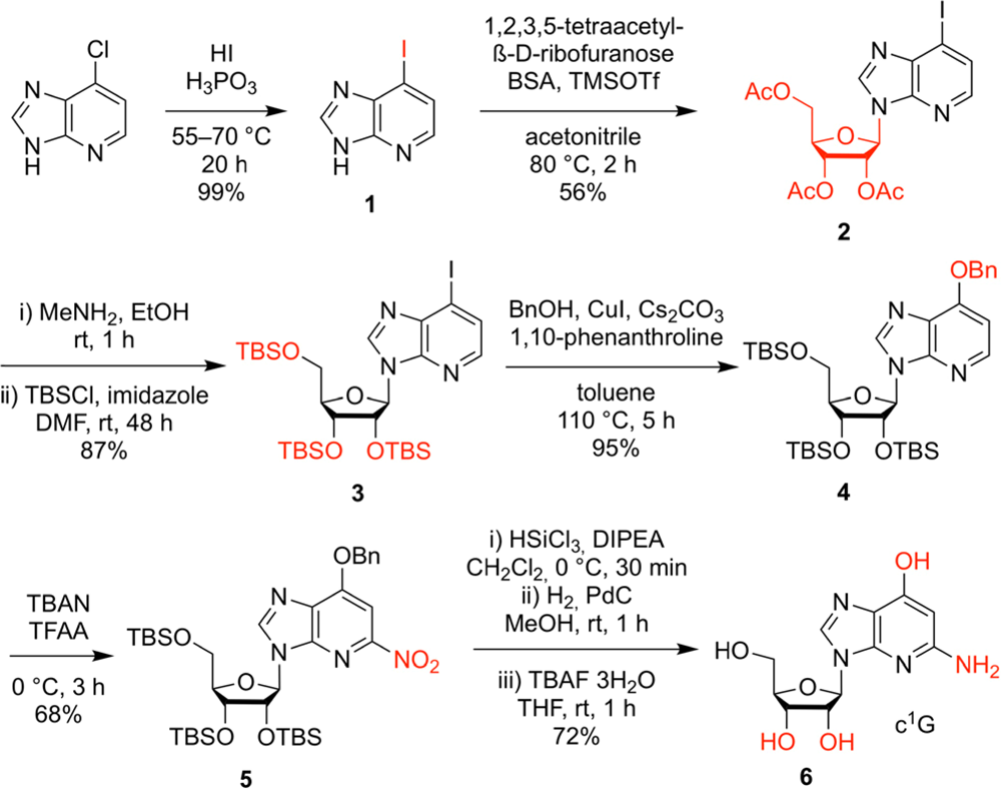
Synthesis of c^1^G Nucleoside **6** and Phosphoramidite Precursor **5** Reaction conditions and yields as indicated. *N*,*O*-Bis(trimethylsilyl)acetamide, BSA; trimethylsilyl trifluoro-methanesulfonate, TMSOTf; *tert*-butyldimethylsilyl, TBS; tetrabutyl-ammonium nitrate, TBAN; trifluoroacetic anhydride, TFAA; *N*,*N*-diisopropylethylamine, DIPEA; tetra-*n*-butylammonium fluoride, TBAF.

For c^1^G nucleoside **6** and the phosphoramidite precursor **5** (Scheme 1), we started the synthesis from commercially available 6-chloro-1-deazapurine, which was quantitatively transformed to the corresponding 6-iodo derivative **1** by treatment with hydroiodic and phosphorous acids. Then, silyl-Hilbert–Johnson nucleosidation of 6-iodo-1-deazapurine **1** and 1,2,3,5-tetra-*O*-acetyl-β-d-ribofuranose in the presence of *N*,*O*-bis(trimethylsilyl)acetamide (BSA) and trimethylsilyl trifluoromethanesulfonate (TMSOTf) provided nucleoside **2** in good yields. The exchange of acetyl to *tert*-butyldimethylsilyl (TBS) protection of the ribose hydroxyl groups (compound **3**) was required to enable efficient copper-catalyzed coupling of the aryl iodide with benzyl alcohol to furnish nucleoside **4**, inspired by the work of the Buchwald laboratory.^33^ Then, site-specific nitration of the *O*^6^-benzyloxy-1-deazapurine moiety was accomplished using tetrabutylammonium nitrate (TBAN) and trifluoroacetic anhydride (TFAA) to obtain nucleoside **5**, in analogy to work by Koomen.^34−36^ Finally, selective reduction of the nitro group was conducted by HSiCl_3_ referring to Benaglia and coworkers,^37^ followed by the cleavage of the benzyl group by hydrogenation, and cleavage of the silyl ethers to give 1-deazaguanosine **6** in 22% overall yield in six steps with five chromatographic purifications; in total, 0.4 g of **6** was obtained in the course of this study.

### Synthesis of c^1^G Phosphoramidite

**Scheme 2:**
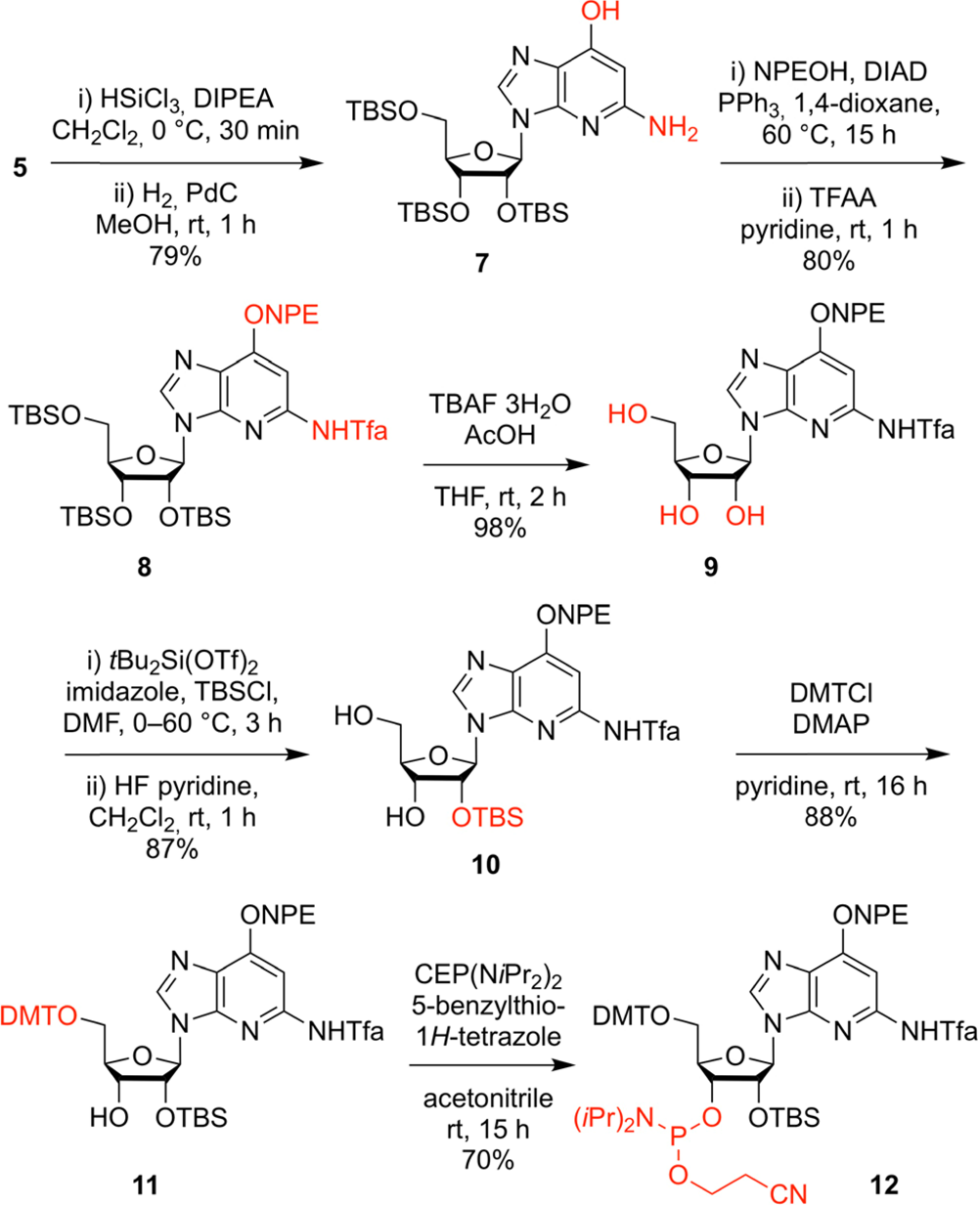
Synthesis of c^1^G Phosphoramidite **12** Reaction conditions and yields as indicated. *N*,*N*-Diisopropylethylamine, DIPEA; (2-nitrophenyl)ethyl, NPE; diisopropyl azodicarboxylate, DIAD; trifluoroacetic anhydride, TFAA; trifluoroacetyl, Tfa; tetrabutylammonium fluoride, TBAF; *tert*-butyldimethylsilyl, TBS; 4,4′-dimethoxytrityl chloride, DMT; 4-(dimethylamino)pyridine, DMAP; cyanoethyl, CE.

We started the synthesis toward c^1^G building block **12** from precursor **5** with the reduction of the nitro group using HSiCl_3_, followed by the cleavage of the benzyl group via hydrogenation providing nucleoside **7** (Scheme 2). Then, the *O*^6^ functionality was protected with a (2-nitrophenyl)ethyl (NPE) moiety applying Mitsunobu reaction conditions, followed by protection of the exocyclic NH_2_ group using trifluoroacetic anhydride (TFAA), resulting in derivative **8**. By the cleavage of the silyl ethers with tetrabutylammonium fluoride (TBAF), triol **9** was quantitatively obtained. Next, the 5′ and 3′ OH groups were simultaneously protected using di-*tert*-butylsilyl bis(trifluoromethanesulfonate) (*t*Bu_2_Si(OTf)_2_),^38,39^ followed by silylation of the 2′-OH group with *tert*-butyldimethylsilyl chloride (TBS-Cl) and subsequent removal of the 5′-*O* and 3′-*O* protection clamp with a solution of HF in pyridine to give compound **10**. The functionalization of the 5′-OH group with 4,4′-dimethoxytrityl chloride was conducted under standard conditions and yielded compound **11**. Finally, the phosphoramidite **12** was generated by treatment with 2-cyanoethyl-*N*,*N*,*N*′,*N*′-tetraisopropylphosphorodiamidite (CEP(N*i*Pr_2_)_2_) in the presence of 5-benzylthio-1*H*-tetrazol (BTT). Starting from precursor **5**, the target compound **12** was synthesized in six steps, with six chromatographic purifications and an overall yield of 33%; in total, 1.1 g of **12** was obtained in the course of this study.

### Synthesis of c^1^G-Modified RNA

**Figure 1:**
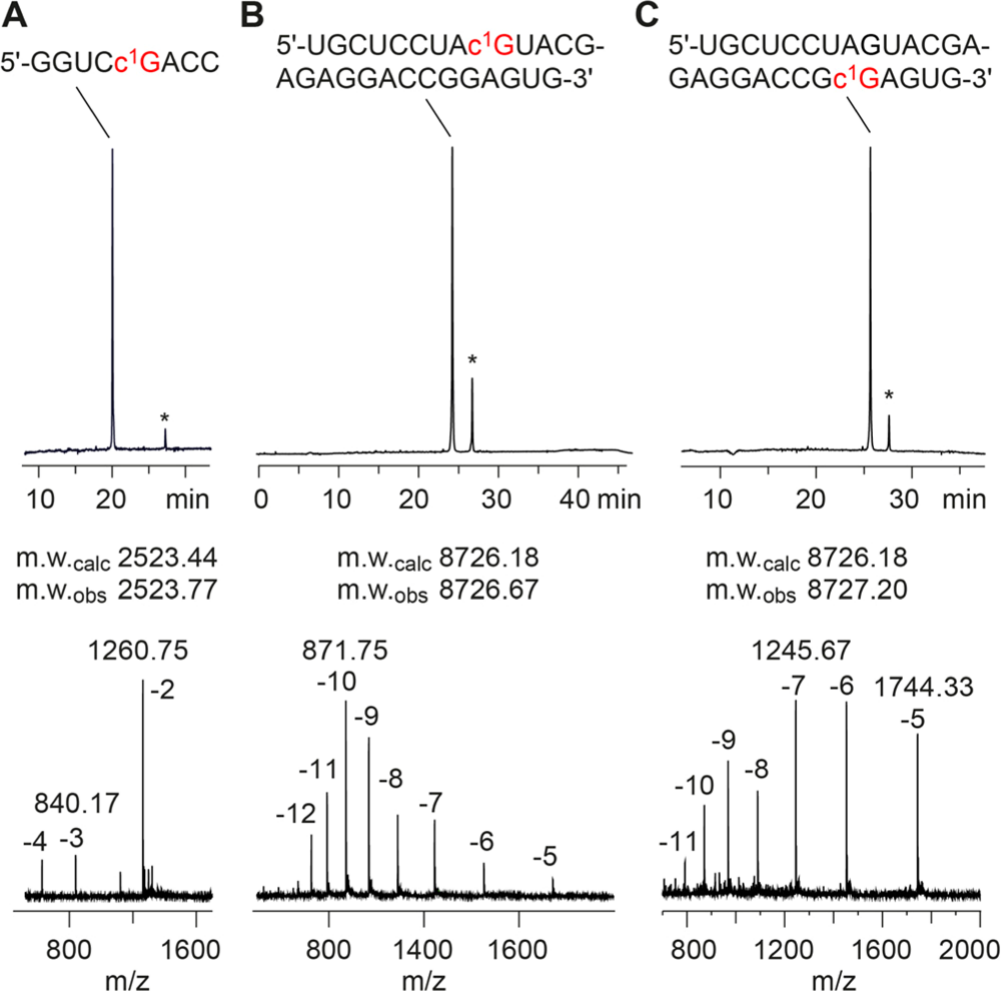
Characterization of c^1^G-modified RNA synthesized by standard RNA solid-phase synthesis using c^1^G building block 12. Anion-exchange HPLC traces (top) of purified 8 nt RNA (A), 27 nt RNA (B), and 27 nt RNA (C), and corresponding LC-ESI mass spectra (bottom). Asterisks indicate RNA dimers cross-linked via a CH_2_ moiety between two c^1^Gs (for detailed mass spectrometric analysis, see Supporting Figure S1). HPLC conditions: Dionex DNAPac column (4 × 250 mm), 80 °C (or as indicated), 1 mL min^–1^, 0–60% buffer B in 45 min; buffer A: Tris–HCl (25 mM), 10 mM NaClO_4_, pH 8.0, 20% acetonitrile; buffer B: Tris–HCl (25 mM), 600 mM NaClO_4_, pH 8.0, 20% acetonitrile. For LC-ESI MS conditions, see the Supporting Information.

The solid-phase synthesis of RNA with site-specific c^1^G modifications was performed using the new building block **12** together with 2′-*O*-TBS protected A, C, G U phosphoramidites, or alternatively, with 2′-*O*-[(triisopropylsilyl)oxy]methyl protected (TOM) amidites.^40,41^ The novel building blocks were coupled with yields higher than 98% according to the trityl assay. The cleavage of the oligonucleotides from the solid support and deprotection were conducted using methylamine/ammonia in water (AMA), followed by treatment with tetra-*n*-butylammonium fluoride (TBAF) in tetrahydrofuran. Salts were removed by size-exclusion chromatography, and RNAs were purified by anion-exchange chromatography under denaturating conditions (60 to 80 °C column temperature; Figure 1 and Supporting Table S1). The molecular weights of the purified RNAs were confirmed by liquid chromatography (LC) electrospray-ionization (ESI) mass spectrometry (MS). The sequences of c^1^G containing RNAs synthesized in the course of this study are listed in Supporting Table S1. Notably, HPLC analysis of the crude deprotected c^1^G containing RNAs displayed a second product that was migrating slower, in particular, when TOM amidites were used. Isolation of this product and mass spectrometric analysis using a high-resolution Fourier-transform ion cyclotron resonance (FT ICR) spectrometer suggested RNA dimers that were cross-linked between two c^1^G nucleosides by a CH_2_ bridge, most likely between their exocyclic amino groups (for details, see Supporting Figure S1). Such a linkage most likely forms during deprotection of the TOM group where formaldehyde emerges as a byproduct.

### Base Pairing Stability of c^1^G-Modified RNA

**Figure 2:**
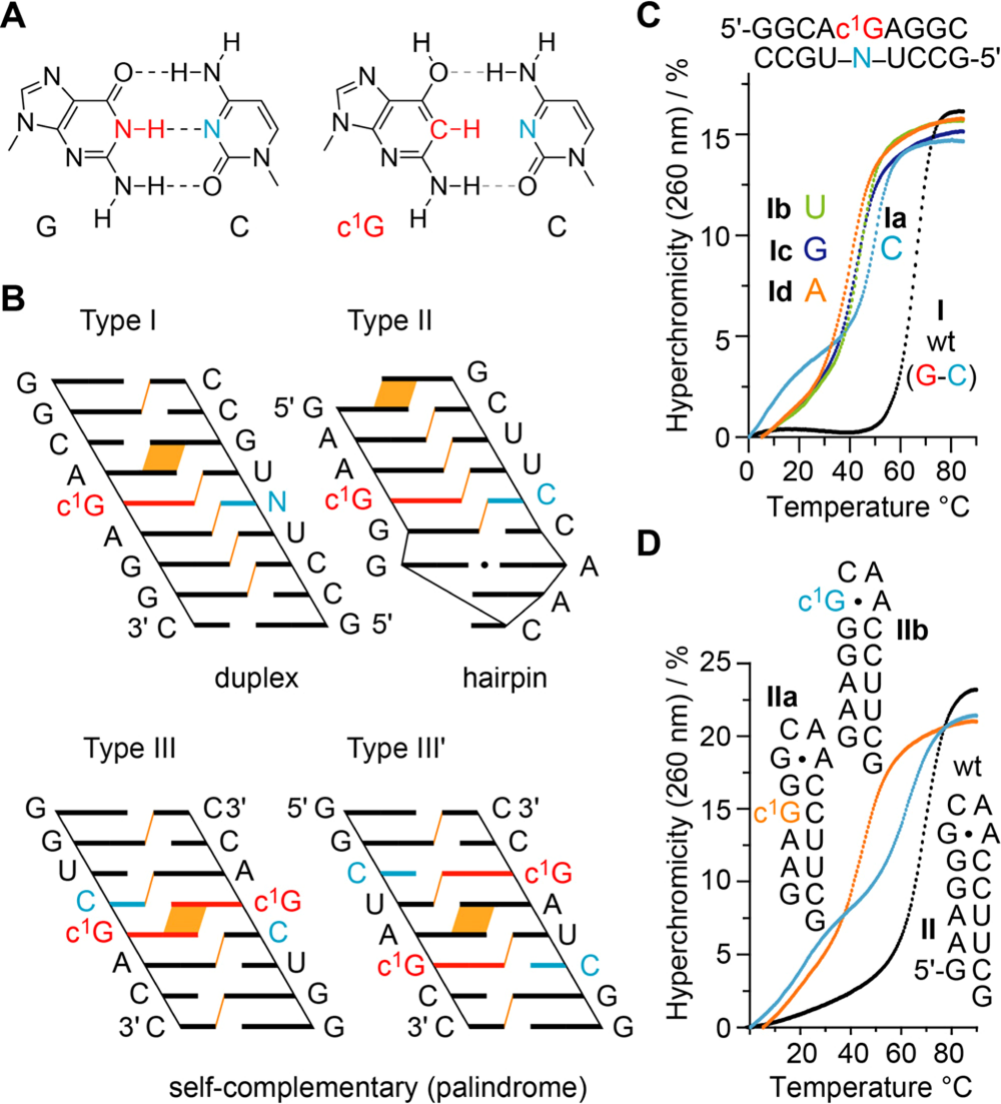
Thermodynamic analysis of base pairing of c^1^G-modified RNAs. (A) Chemical structure of Watson Crick G–C base pair juxtaposed to a hypothetic c^1^G–C pair. (B) Sequence design in cartoon presentation to highlight stacking interactions (purine–purine and purine–pyrimidine interstrand stacking indicated in orange). (C) Overlay of UV-melting profiles of type I RNA with c^1^G-N mismatches (N = A, C, G, or U). (D) Overlay of UV-melting profile of type II RNA hairpin with c^1^G in stem and loop, respectively. Conditions: *c*(RNA) = 12 μM; 10 mM Na_2_HPO_4_, 150 mM NaCl, pH 7.0.

In principle, for the nucleobase of c^1^G, tautomeric forms and distinct rotamers have to be considered. An earlier study reported the energy differences of 9-methyl-1-deazaguanine tautomers and rotamers estimated by ab initio calculations.^27^ It was found that the c^1^G tautomer/*syn*-rotamer that we show in Figure 2A is the most stable one, followed by the *anti*-rotamer with 6-OH providing H-donor properties at the Watson Crick face, being 4.7 kcal/mol less stable. Importantly, the N3–H pyridone tautomer is 20.4 kcal/mol less stable.^27^

c^1^G is expected to impair Watson–Crick pairing because the central N1–H of G is replaced by C–H, thereby depriving the capability for the formation of strong hydrogen bonds (Figure 2A). The design of the RNA double helices investigated is shown in Figure 2B. The first motif (Type I) represents a bimolecular duplex of nine base pairs with a single c^1^G modification in the center. The second motif (Type II) is a hairpin with a GCAA loop (extrastable GNRA) and c^1^G residing in the center of its short stem. The third RNA motif (Type III and III′) consists of palindromic RNA of eight base pairs and equivalent purine–pyrimidine stacking patterns with the c^1^Gs either directly stacked toward each other, or separated by two standard base pairs. The type III/III′ design is very sensitive for the impact arising from a modification on base pairing. With only two or three regular Watson–Crick base pairs next to the modification, the nucleation of such duplexes can become significantly hindered.^42,43^ Thus, these RNA palindromes are anticipated to significantly respond to the c^1^G modification reflected in changes of the thermodynamic pairing parameters (*T*_m_, Δ*G*, Δ*H*, Δ*S*).

The thermodynamic data we obtained for the three RNA systems by UV-spectroscopic melting profile measurements are summarized in Table 1 (for the corresponding melting profiles, see the Supporting Figures S2 to S11).^44,45^ The native type I RNA **I** melts at 66.7 °C (Figure 2C). 1-Deazaguanine opposite of cytosine (**Ia**) destabilizes the duplex by 15.8 °C. Destabilization is even more pronounced if U, G, and A are the mismatch partner (−21.8 °C for **Ib**, −23.2 °C for **Ic**, and −26.1 °C for **Id**, respectively). For the hairpin RNA (Type II), c^1^G opposite of C (**IIa**) also decreases the melting temperature compared to the native hairpin **II** (by −27.9 °C) (Figure 2D). We note that duplex **Ia** shows a second melting transition at lower temperature, around 18 °C (Figure 2C). This may arise from a higher order structure (e.g., triplex formation) that we were not able to characterize in detail.

**Table 1:** Thermodynamic Parameters of c^1^G-Modified RNA (and Unmodified References) Obtained by UV Melting Profile Analysis^a^

#	sequence (5′ → 3′)	*T*_m_ [°C]	Δ*T*_m_	Δ*G*°_298_ [kcal mol^–1^]	Δ*H*° [kcal mol^–1^]	Δ*S*° [cal mol^–1^ K^–1^]
I	GGCA**G**AGGC / GCCU**C**UGCC	66.7		–16.5 ± 0.4	–79.7 ± 4.6	–212 ± 14
Ia	GGCA**c**^**1**^**G**GAGGC / GCCU**C**UGCC	50.9	–15.8	–13.1 ± 0.9	–79.9 ± 7.7	–224 ± 23
Ib	GGCA**c**^**1**^**G**AGGC / GCCUC**U**GCC	45.0	–21.8	–11.4 ± 0.2	–73.6 ± 3.9	–209 ± 12
Ic	GGCA**c**^**1**^**G**AGGC / GCCUC**G**GCC	43.5	–23.2	–10.9 ± 0.3	–70.9 ± 5.2	–201 ± 16
Id	GGCA**c**^**1**^**G**AGGC / GCCUC**A**GCC	40.6	–26.1	–10.2 ± 0.1	–69.7 ± 3.5	–200 ± 11
II	GAA**G**G-GCAA-C**C**UUCG (hairpin)	72.7		–6.6 ± 0.1	–49.8 ± 0.8	–145 ± 3
IIa	GAA**c**^**1**^**G**G-GCAA-C**C**UUCG (hairpin)	44.8	–27.9	–2.8 ± 0.4	–48.5 ± 3.2	–153 ± 9
IIb	GAA**G**G-**c**^**1**^**G**CA**A**-C**C**UUCG (hairpin)	63.1	–9.6	–6.2 ± 0.2	–55.0 ± 2.7	–165 ± 8
III	GGU**CG**ACC (palindrome)	58.3		–13.2 ± 0.9	–64.6 ± 8.6	–172 ± 26
IIIa	GGU**Cc**^**1**^**G**ACC (palindrome)	22.4	–35.9	–6.3 ± 0.2	–55.0 ± 2.7	–164 ± 9
III′	GG**C**UA**G**CC (palindrome)	60.7		–14.5 ± 1.1	–72.3 ± 9.5	–194 ± 28
III′a	GG**C**UA**c**^**1**^**G**CC (palindrome)	24.1	–36.5	–6.6 ± 0.2	–58.4 ± 1.1	–174 ± 4

a Buffer: 10 mM Na_2_HPO_4_, 150 mM NaCl, pH 7.0. *T*_m_ values are listed at a concentration of 12 μM RNA. The estimated errors of UV-spectroscopically determined *T*_m_ values are ±0.2 °C. Δ*H* and Δ*S* values were obtained by van’t Hoff analysis according to refs (44, 45). Errors for Δ*H* and Δ*S*, arising from noninfinite cooperativity of two-state transitions and from the assumption of a temperature-independent enthalpy, are typically 10–15%. Additional error is introduced when free energies are extrapolated far from melting transitions; errors for Δ*G* are typically 3–5%. We note that for the biphasic profiles of **Ia** and **IIb**, the *T*_m_ values and the errors were calculated for the second melting transition (between 30 and 85 °C).

To further elucidate the impact of c^1^G on base pairing, we investigated the short palindromic RNAs that are particularly sensitive to double helix nucleation as mentioned above.^42,43^ Indeed, for c^1^G, the destabilization in both palindromic RNAs was large, reflected in −35.9/–36.5 °C reduced *T*_m_ values (**IIIa** and **III′a**), accounting for −17.9/–18.3 °C destabilization per single modification which is higher compared to the destabilization that we observed for a single c^1^G–C base pair in the bimolecular 9 bp duplex **Ia** with four regular – and hence nucleation-supportive – Watson–Crick base pairs at both 5′ and 3′ directions to the modification site.

Finally, we mention that the replacement of G in the *syn*G•A Hoogsteen base pair of a GNRA loop in hairpin **IIb** was tolerated with significantly less decrease in stability (Figure 2D). This is reasonable because the G-N1-H atom is not involved in H-bonding in the *syn*G•A Hoogsteen pair. Of note, we observe a second low temperature melting transition for **IIb**, which may arise from competitive formation of a mismatched duplex.

### Acid–Base Properties of 1-Deazaguanine

**Figure 3:**
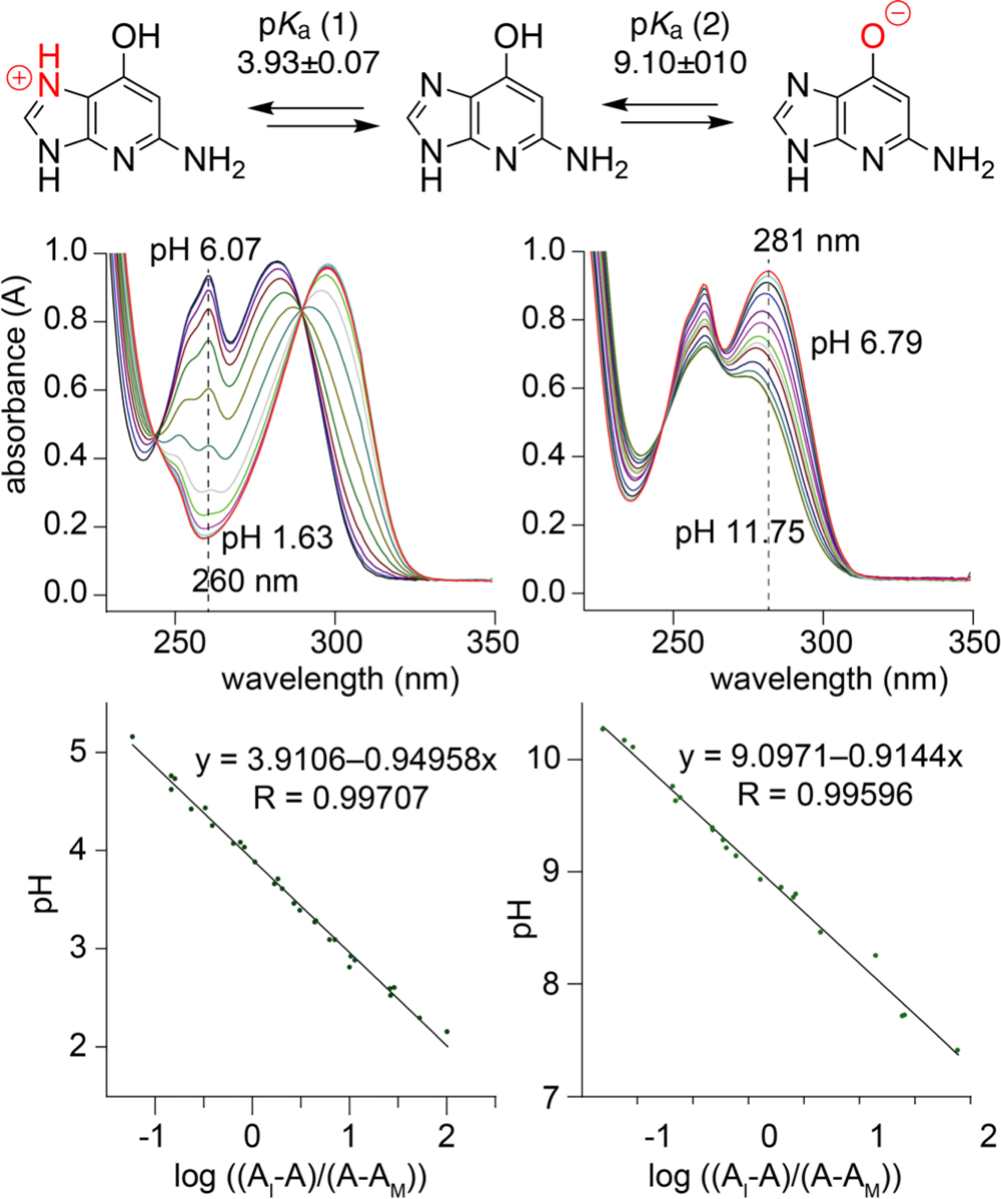
Determination of p*K*_a_ values of the c^1^G nucleobase by pH-dependent UV-spectroscopic titration experiments. Conditions: *c*(c^1^G) = 95 μM; 100 mM KCl, 25 mM citric acid (p*K*_a_(1)) and 25 mM TRIS (p*K*_a_(2)).

To understand base-pairing and catalytic properties of nucleobases in functional RNA, knowledge about their acid–base properties is crucial.^7,26^ To quantify the acid–base properties of c^1^G, we conducted pH-dependent UV-spectroscopic titration experiments. Figure 3 shows an overlay of spectra for the c^1^G nucleobase that were used for p*K*_a_ determinations. A value of 3.93 ± 0.07 (p*K*_a_ 1) was obtained, attributed to the protonation of N7 (Supporting Figure S12). The second value of 9.10 ± 0.10 (p*K*_a_ 2) was attributed to the deprotonation of the 6-OH group. The p*K*_a_ values are thus comparable to the ones of guanine, which range from 9.2 to 9.6 (p*K*_a_ 1, N1–H) and 3.2 to 3.3 (p*K*_a_ 2, N7), respectively.^46^

### X-Ray Structure Analysis of a c^1^G-Modified RNA

**Figure 4:**
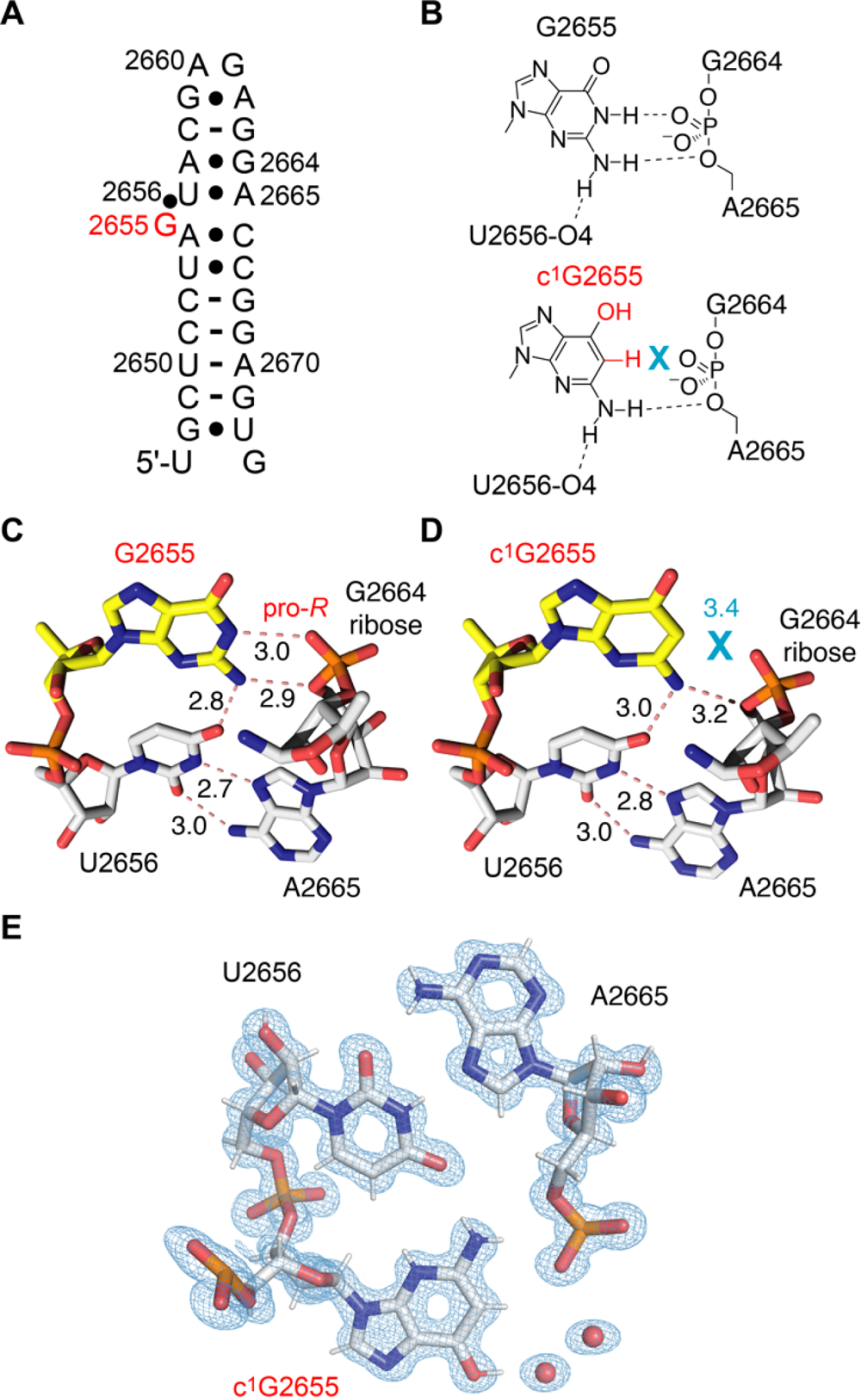
X-ray structure of c^1^G-modified RNA at 0.9 Å resolution. (A) Secondary structure of the *E. coli* sarcin/ricin stem-loop (SRL) RNA used for crystallization. The position for c^1^G nucleotide replacement is highlighted in red. (B) Chemical structure of G2655 interacting with the phosphate between G2664 and A2665 based on the crystal structure of native RNA PDB ID 3DVZ (top) and comparison to the c^1^G2655 interaction in the same RNA. (C) View on the base triple U2656-A2665-G2655 (PDB ID 3DVZ). (D) View on the base triple U2656-A2665-c^1^G2655 (PDB ID 7QP2). (E) 2*F*_obs_ – *F*_calc_ electron density map contoured at 1.5 σ level showing the c^1^G2655 containing triple (PDB ID 7QP2). Numbers are distances in Angström (Å).

To shed further light on the structural impact of c^1^G in RNA, we aimed at a high-resolution X-ray crystallographic analysis. We utilized the 27 nt fragment of the *E. coli* 23S rRNA sarcin/ricin loop (SRL), which is a frequently applied crystallization scaffold (Figure 4A).^47,48^ For the replacement of G by c^1^G, we tested three different positions, including nucleotide G2669 which forms a Watson–Crick base pair with C2651 in the regular A-form double helical region, G2659 which forms a Hoogsteen pair with A2662 in the loop, and G2655 which interacts with the phosphate of G2664 in a bidentate fashion (Figure 4B, C). From these three c^1^G-modified RNAs, only c^1^G2655-modified SRL RNA provided crystals that diffracted to atomic resolution (0.9 Å) (Supplementary Table S2). X-ray structure determination demonstrated that the c^1^G nucleobase is well defined in the electron density maps for the c^1^G-modified RNA (Figure 4D, E). The c^1^G-modified RNA structure superimposed with the unmodified RNA displayed a root-mean-square deviation (rmsd) of 0.09 Å (within the errors on coordinates of 0.09 Å). Direct comparison of the base triples U2656-A2665-G2655 (Figure 4C) and U2656-A2665-c^1^G2655 (Figure 4D) reveals that with the weakening (or loss) of the G2655 N1–H···O–P H-bond, c^1^G slightly opens up by retaining the H-bond between c^1^G2655 2-NH_2_ and O4 of U2656.

A comparison of the melting profiles of wild-type and c^1^G modified SRL hairpins indicated a modest weakening of the fold (Supporting Figure S13).

Taken together, our crystallization experiments imply that c^1^G does not significantly affect an RNA fold as long as it is not replacing G in a Watson–Crick base pair. The weakening (or loss) of an H-bond to the phosphate backbone seems better tolerated and allowed crystallization and structure solution.

### Base Pairing Mode Switched by c^1^G

**Figure 5:**
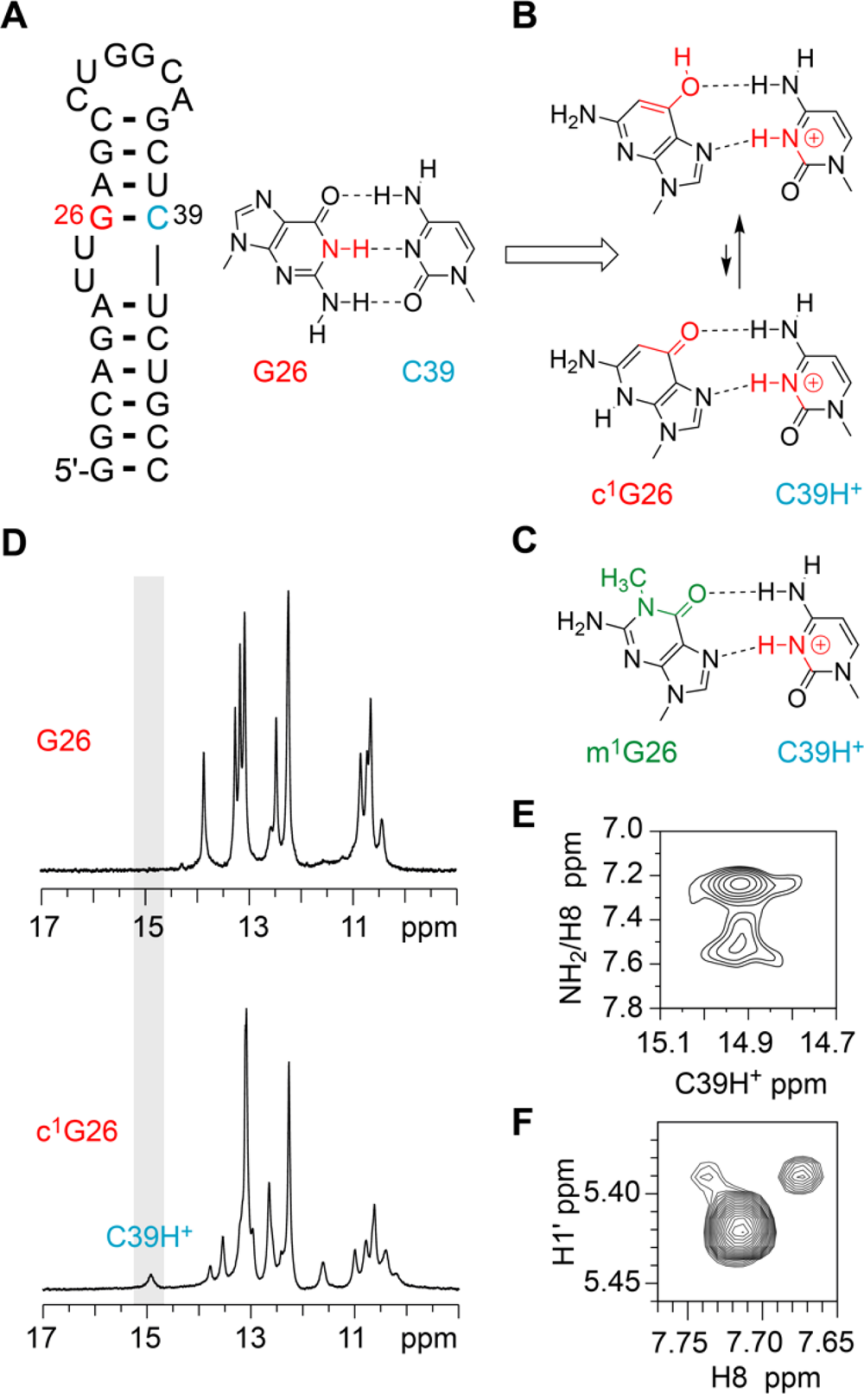
c^1^G-induced base pair switch analyzed by NMR spectroscopy. (A) Secondary structure of the HIV2 TAR RNA. The position for c^1^G nucleotide replacement is highlighted in red. (B) Chemical structure of the G26-C39 WC bp, and predicted *syn* c^1^G26-C39H^+^ HG bp (two tautomeric forms). (C) Comparison of c^1^G26-C39H^+^ base pair geometry to the *syn* m^1^G-C39H^+^ HG bp.^49^ (D) Comparison of ^1^H imino proton spectra of unmodified and c^1^G-modified HIV2 TAR RNA. (E) Tentative assignment of ^1^H,^1^H-NOESY spectrum (c^1^G-modified HIV2-TAR) showing the through space correlations of C39H^+^ to its NH_2_ group (and possibly to C8–H of c^1^G26). (**F**) ^1^H,^1^H-NOESY spectrum (c^1^G-modified HIV2-TAR) showing the correlations of c^1^G26 H1′ to C8–H of c^1^G26. Assignments supported by comparison to the corresponding TOCSY spectra (Supporting Figure S10) and ref (52). Conditions: 25 mM NaCl, 10% D_2_O, pH 5.8.

In A-form RNA, Hoogsteen (HG) base pairs are energetically disfavored relative to Watson–Crick (WC) pairs. With 1-deazaguanosine in our hands, however, we were wondering if stable HG pairing might become favorable. We thereby focused on the human immunodeficiency virus type 2 (HIV-2) transactivation response element (TAR) RNA, where a G26-C39 WC bp is adjacent to a dinucleotide bulge (Figure 5A). It was demonstrated earlier that upon replacement of G26 by 1-methylguanosine (m^1^G26), the formation of a HG base pair with C39 occurs (Figure 5B, C).^49^ While in this case N1-methylation represents a severe steric block at the Watson Crick face, we intended to test the hypothesis if a simple shape-complementary modification (such as c^1^G) is sufficient to switch the pairing mode (Figure 5B, C). Indeed, our NMR spectroscopic analysis of HIV-2 TAR RNA containing c^1^G26 revealed that the HG base pair c^1^G26(*syn*)-C39H^+^ forms in a comparable manner. Characteristically, we observed a downfield shifted imino proton at ∼15 ppm (Figure 5D, Supporting Figure S14) and downfield shifted amino protons (Figure 5E) of C39H^+^ that is hydrogen-bonded to *syn* c^1^G26. Furthermore, a strong intra-nucleotide H1′–H8 NOE cross-peak (Figure 5F) is consistent with the *syn* conformation of the c^1^G26 base.

### Active-Site c^1^G Impedes Twister Ribozyme Cleavage

Deazanucleobase-modified RNAs are frequently applied in atomic mutagenesis studies of ribozymes to shed light on the mechanism of the chemical reactions they catalyze.^10,18,24,50^ In particular, atomic mutagenesis experiments led to an in-depth understanding of general acid–base catalysis of small nucleolytic ribozymes that cleave their phosphodiester backbone, revealing the functionally crucial imino groups of purines and pyrimidines in the active site. For instance, this concerns the twister ribozyme^51^ where proton transfer from the (protonated) N3 of a conserved adenine (A6) at the cleavage site to the 5′-*O* leaving group significantly contributes to reaction catalysis; the replacement of this adenine by c^3^A or c^1^c^3^A rendered the twister ribozyme inactive.^15,52^ Another example is a phosphodiester cleavage by the pistol ribozyme.^53^ Replacements of active site purines by c^3^A, c^1^A, and c^7^G revealed the key residue – a highly conserved guanine (G33) – that serves as inner sphere coordination site for a hydrated Mg^2+^ ion, thereby likely providing a 1st shell water molecule as general acid for protonation of the 5′-*O* leaving group in the course of the reaction.^16,17^

**Figure 6:**
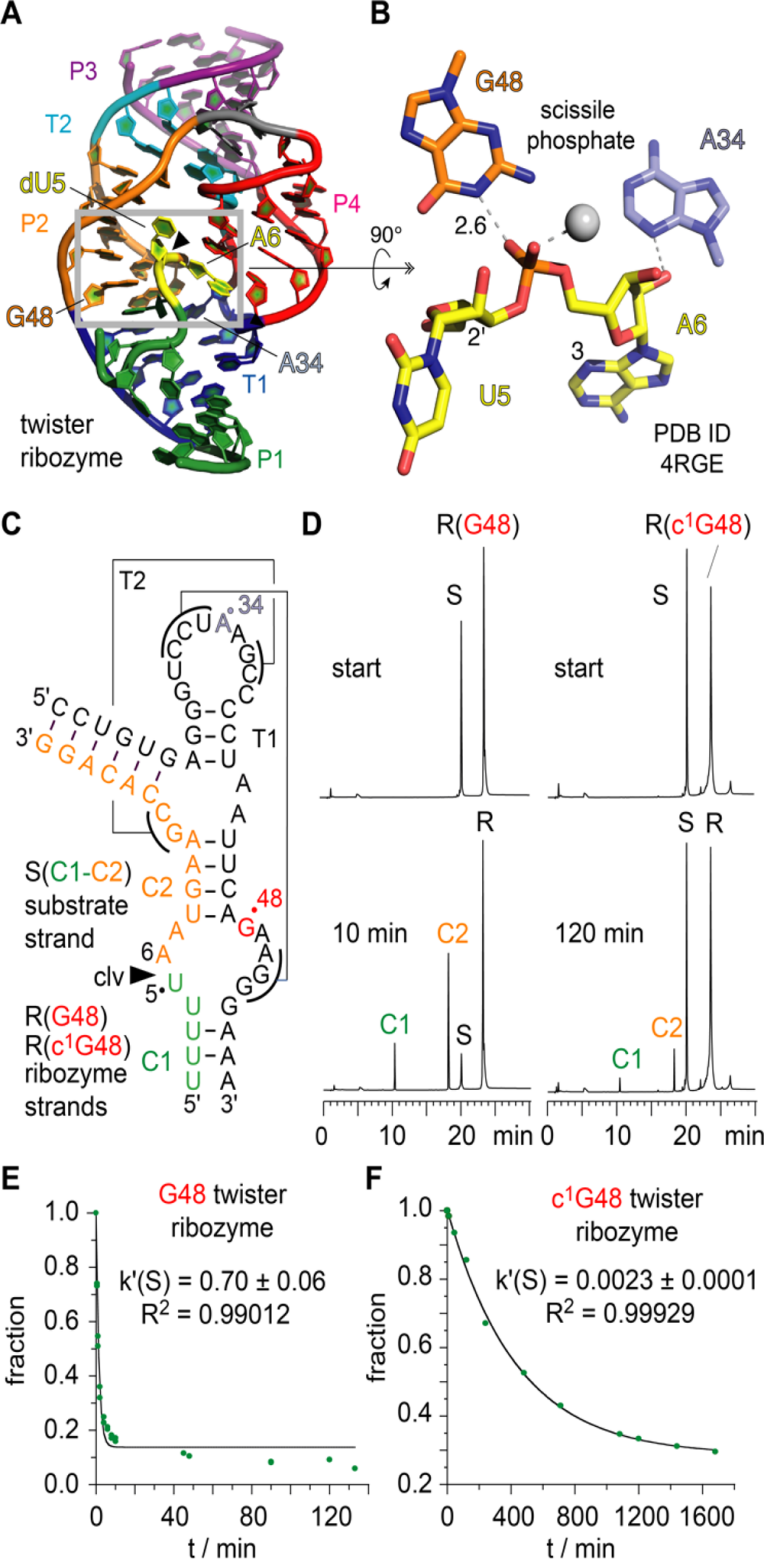
Atomic mutagenesis of the twister ribozyme: impact of an active site G-to-c^1^G mutation on activity to elucidate the mechanism of the phosphordiester cleavage. (A) Crystal structure of the twister ribozyme in a precatalytic state (PDB ID 4RGE).^54^ Active site highlighted by gray frame. Cleavage site dU5-A6 is colored yellow. (B) Close-up view showing the interaction of guanine-48 with the scissile phosphate; the 2′-OH nucleophile is modeled on U5; distance in Å. (C) Secondary structure of the two-strand ribozyme assembly used for functional cleavage assays. (D) HPLC traces of wild-type G48 (left) and c^1^G48 modified (right) ribozyme at two time points illustrate that product formation of the c^1^G-modified ribozyme is significantly impeded under otherwise same reaction conditions. Cleavage rate determination of wild-type G48 (E) and c^1^G48 (F) ribozymes.

Access to c^1^G-modified RNA now allows evaluation of active site guanines that are suspected to be involved in reaction catalysis via their Watson–Crick face. To exemplify this, we picked the three-way junctional twister ribozyme,^53^ for which several structures of precatalytic states were solved by X-ray crystallography^15,54−58^ and structural dynamics elucidated by smFRET imaging.^10,59^ Bases U5 and A6 at the cleavage site are splayed apart, with a guanine (G48 for PDB ID 4RGE^54^ and 5DUN^15^) in a hydrogen bond distance (2.6 Å) between the N1 atom and the nonbridging pro-*R* oxygen of the scissile phosphate (Figure 6A). The structures implicate that G48 may play a significant role in phosphorane transition state stabilization. Furthermore, several studies propose the hypothesis of concerted general acid–base catalysis for twister in which G48 acts as the general base (Figure 6B).^52,56,60^

With c^1^G in hand, we can now probe whether or not the NH donor of the Watson–Crick face of the suspected G48 is indeed a determinant in reaction catalysis (i.e., β and/or γ-catalysis according to refs (61, 62)). Involvement of G48 as general base in phosphodiester cleavage catalysis implies either N1-deprotonation or enol tautomerization or at least hydrogen bonding to activate the attacking 2′-OH nucleophile. Also, stabilization of the pentavalent phosphorane transition state is conceivable as extrapolated from the G48N1–H···O=P interaction seen in the crystal structure.^54^ All these scenarios are severely affected upon the replacement of G48 by c^1^G; we therefore anticipated that cleavage becomes abolished. We, however, found that cleavage still occurs, albeit with a 275-fold reduction in rate (Figure 6C–F and Supporting Figures S15, S16).

The remaining cleavage activity indicates that the other catalytic determinants (i.e., α- and δ- catalysis according to refs (61) and (62)) are sufficient to achieve residual activity of c^1^G48-modified twister ribozyme. We further note that the 6-OH group of c^1^G (possessing a comparable p*K*_a_ to N1–H of G) is dislocated in comparison with N1 (in G) and therefore is not likely to be able to efficiently take over its role. However, the 2-NH_2_ group of guanine is present also in c^1^G, and therefore, this NH_2_ group can contribute to stabilization of the phosphorane transition state (together with a remaining weak stabilization originating from a C1–H interaction with phosphorane).

## Conclusions

Our study introduces robust syntheses of c^1^G, the corresponding phosphoramidite, and c^1^G modified RNA. The synthetic foundation enabled comprehensive analysis of the biophysical properties of such modified RNA, and furthermore, enabled c^1^G atomic mutagenesis in functional RNA assays. This led to evidence for c^1^G switching the mode of base pairing from Watson–Crick to Hoogsteen. Moreover, the approach allows direct evaluation of ribozyme mechanistic proposals that claim a catalytic role for guanosines via their N1 position, the central H-donor of their Watson–Crick face. Beyond twister, such guanosines are found in many ribozymes including twister sister, pistol, hatchet, and the most recently discovered RNA methyltransferase ribozymes.^18,24,63^ Functional atomic mutagenesis relying on c^1^G RNA modifications will contribute to achieve an in-depth understanding of RNA catalysis of ribozymes that exhibit a much broader reactivity scope than previously anticipated.

## Supporting Information Available

Additional methods information, synthetic procedures and NMR spectra of all compounds, table of all RNAs synthesized and MS data, RNA melting curves, X-ray data collection and crystallographic refinement statistics, NMR spectra, and HPLC cleavage assays of the twister ribozyme (PDF)

## References

[ref1] Polacek N. Atomic mutagenesis of the ribosome: towards a molecular understanding of translation. Chimia 2013, 67, 322–326. 10.2533/chimia.2013.322. 23863265

[ref2] Hoernes T. P.; Clementi N.; Juen M. A.; Shi X.; Faserl K.; Willi J.; Gasser C.; Kreutz C.; Joseph S.; Lindner H.; Hüttenhofer A.; Erlacher M. D. Atomic mutagenesis of stop codon nucleotides reveals the chemical prerequisites for release factor-mediated peptide release. Proc. Natl. Acad. Sci. U. S. A. 2018, 115, E382–E389. 10.1073/pnas.1714554115. 29298914 PMC5776981

[ref3] Hu T.; Suter S. R.; Mumbleau M. M.; Beal P. A. TLR8 activation and inhibition by guanosine analogs in RNA: Importance of functional groups and chain length. Bioorg. Med. Chem. 2018, 26, 77–83. 10.1016/j.bmc.2017.11.020. 29174509 PMC5733708

[ref4] Forconi M.; Benz-Moy T.; Gleitsman K. R.; Ruben E.; Metz C.; Herschlag D. Exploring purine N7 interactions via atomic mutagenesis: the group I ribozyme as a case study. RNA 2012, 18, 1222–1229. 10.1261/rna.031567.111. 22543863 PMC3358644

[ref5] Seela F.; Debelak H.; Usman N.; Burgin A.; Beigelman L. 1-Deazaadenosine: synthesis and activity of base-modified hammerhead ribozymes. Nucleic Acids Res. 1998, 26, 1010–1018. 10.1093/nar/26.4.1010. 9461461 PMC147336

[ref6] Kapinos L. E.; Operschall B. P.; Larsen E.; Sigel H. Understanding the acid-base properties of adenosine: the intrinsic basicities of N1, N3 and N7. Chem. – Eur. J. 2011, 17, 8156–8164. 10.1002/chem.201003544. 21626581

[ref7] Krishnamurthy R. Role of pK(a) of nucleobases in the origins of chemical evolution. Acc. Chem. Res. 2012, 45, 2035–2044. 10.1021/ar200262x. 22533519 PMC3525050

[ref8] Acharya P.; Cheruku P.; Chatterjee S.; Acharya S.; Chattopadhyaya J. Measurement of nucleobase pKa values in model mononucleotides shows RNA-RNA duplexes to be more stable than DNA-DNA duplexes. J. Am. Chem. Soc. 2004, 126, 2862–2869. 10.1021/ja0386546. 14995203

[ref9] Bande O.; Braddick D.; Agnello S.; Jang M.; Pezo V.; Schepers G.; Rozenski J.; Lescrinier E.; Marlière P.; Herdewijn P. Base pairing involving artificial bases *in vitro* and *in vivo*. Chem. Sci. 2016, 7, 995–1010. 10.1039/c5sc03474d. 29896368 PMC5954848

[ref10] Micura R.; Höbartner C. Fundamental studies of functional nucleic acids: aptamers, riboswitches, ribozymes and DNAzymes. Chem. Soc. Rev. 2020, 49, 7331–7353. 10.1039/d0cs00617c. 32944725

[ref11] Fuchs E.; Falschlunger C.; Micura R.; Breuker K. The effect of adenine protonation on RNA phosphodiester backbone bond cleavage elucidated by deaza-nucleobase modifications and mass spectrometry. Nucleic Acids Res. 2019, 47, 7223–7234. 10.1093/nar/gkz574. 31276590 PMC6698743

[ref12] Spitale R. C.; Volpini R.; Heller M. G.; Krucinska J.; Cristalli G.; Wedekind J. E. Identification of an imino group indispensable for cleavage by a small ribozyme. J. Am. Chem. Soc. 2009, 131, 6093–6095. 10.1021/ja900450h. 19354216 PMC2692201

[ref13] Spitale R. C.; Volpini R.; Mungillo M. V.; Krucinska J.; Cristalli G.; Wedekind J. E. Single-atom imino substitutions at A9 and A10 reveal distinct effects on the fold and function of the hairpin ribozyme catalytic core. Biochemistry 2009, 48, 7777–7779. 10.1021/bi9011622. 19634899 PMC2754253

[ref14] Zheng L.; Falschlunger C.; Huang K.; Mairhofer E.; Yuan S.; Wang J.; Patel D. J.; Micura R.; Ren A. Proc. Natl. Acad. Sci. U. S. A. 2019, 116, 10783–10791. 10.1073/pnas.1902413116. 31088965 PMC6561176

[ref15] Košutić M.; Neuner S.; Ren A.; Flür S.; Wunderlich C.; Mairhofer E.; Vušurović N.; Seikowski J.; Breuker K.; Höbartner C.; Patel D. J.; Kreutz C.; Micura R. A Mini-Twister Variant and Impact of Residues/Cations on the Phosphodiester Cleavage of this Ribozyme Class. Angew. Chem., Int. Ed. 2015, 54, 15128–15133. 10.1002/anie.201506601. PMC4715771 26473980

[ref16] Neuner S.; Falschlunger C.; Fuchs E.; Himmelstoss M.; Ren A.; Patel D. J.; Micura R. Atom-Specific Mutagenesis Reveals Structural and Catalytic Roles for an Active-Site Adenosine and Hydrated Mg^2+^ in Pistol Ribozymes. Angew. Chem., Int. Ed. 2017, 56, 15954–15958. 10.1002/anie.201708679. 29098759

[ref17] Teplova M.; Falschlunger C.; Krasheninina O.; Egger M.; Ren A.; Patel D. J.; Micura R. Crucial Roles of Two Hydrated Mg^2+^ Ions in Reaction Catalysis of the Pistol Ribozyme. Angew. Chem., Int. Ed. 2020, 59, 2837–2843. 10.1002/anie.201912522. PMC7027511 31804735

[ref18] Scheitl C. P. M.; Ghaem Maghami M.; Lenz A. K.; Höbartner C. Site-specific RNA methylation by a methyltransferase ribozyme. Nature 2020, 587, 663–667. 10.1038/s41586-020-2854-z. 33116304 PMC7116789

[ref19] Erlacher M. D.; Lang K.; Shankaran N.; Wotzel B.; Hüttenhofer A.; Micura R.; Mankin A. S.; Polacek N. Chemical engineering of the peptidyl transferase center reveals an important role of the 2′-hydroxyl group of A2451. Nucleic Acids Res. 2005, 33, 1618–1627. 10.1093/nar/gki308. 15767286 PMC1065261

[ref20] Erlacher M. D.; Lang K.; Wotzel B.; Rieder R.; Micura R.; Polacek N. Efficient ribosomal peptidyl transfer critically relies on the presence of the ribose 2’-OH at A2451 of 23S rRNA. J. Am. Chem. Soc. 2006, 128, 4453–4459. 10.1021/ja0588454. 16569023

[ref21] Lang K.; Erlacher M. D.; Wilson D. N.; Micura R.; Polacek N. The role of 23S ribosomal RNA residue A2451 in peptide bond synthesis revealed by atomic mutagenesis. Chem. Biol. 2008, 15, 485–492. 10.1016/j.chembiol.2008.03.014. 18439847

[ref22] Polikanov Y. S.; Steitz T. A.; Innis C. A. A proton wire to couple aminoacyl-tRNA accommodation and peptide-bond formation on the ribosome. Nat. Struct. Mol. Biol. 2014, 21, 787–793. 10.1038/nsmb.2871. 25132179 PMC4156881

[ref23] Das S. R.; Piccirilli J. A. General acid catalysis by the hepatitis delta virus ribozyme. Nat. Chem. Biol. 2005, 1, 45–52. 10.1038/nchembio703. 16407993

[ref24] Flemmich L.; Moreno S.; Heel S.; Breuker K.; Micura R. A natural riboswitch scaffold with self-methylation activity. Nat. Commun. 2021, 12, 3877 10.1038/s41467-021-24193-7. 34162884 PMC8222354

[ref25] Mairhofer E.; Flemmich L.; Kreutz C.; Micura R. Access to 3-Deazaguanosine Building Blocks for RNA Solid-Phase Synthesis Involving Hartwig-Buchwald C-N Cross-Coupling. Org. Lett. 2019, 21, 3900–3903. 10.1021/acs.orglett.9b00855. 31081638

[ref26] Bereiter R.; Himmelstoß M.; Renard E.; Mairhofer E.; Egger M.; Breuker K.; Kreutz C.; Ennifar E.; Micura R. Impact of 3-deazapurine nucleobases on RNA properties. Nucleic Acids Res. 2021, 49, 4281–4293. 10.1093/nar/gkab256. 33856457 PMC8096147

[ref27] Kojima N.; Inoue K.; Nakajima-Shibata R.; Kawahara S.-i.; Ohtsuka E. A new, but old, nucleoside analog: the first synthesis of 1-deaza-2′-deoxyguanosine and its properties as a nucleoside and as oligodeoxynucleotides. Nucleic Acids Res. 2003, 31, 7175–7188. 10.1093/nar/gkh154. 14654693 PMC291881

[ref28] Kojima N.; Sugino M.; Mikami A.; Ohtsuka E.; Komatsu Y. Generation of an abasic site in an oligonucleotide by using acid-labile 1-deaza-2′-deoxyguanosine and its application to postsynthetic modification. Org. Lett. 2005, 7, 709–712. 10.1021/ol0474498. 15704931

[ref29] Schelling J.; Salemink C. Deazapurine derivatives XIV. The synthesis of 1-deazaguanosine. Recl.: J. R. Neth. Chem. Soc. 1975, 94, 153–156. 10.1002/recl.19750940704.

[ref30] Cline B. L.; Panzica R. P.; Townsend L. B. Syntheses of 5-Amino-3-(Beta-D-Ribofuranosyl)Imidazo[4,5-b]Pyridin-7-One (1-Deazaguanosine) and Related Nucleosides. J. Heterocycl. Chem. 1978, 15, 839–847. 10.1002/jhet.5570150524.

[ref31] Schelling J.; Salemink C. Deazapurine derivatives XIII 5,7-Disubstituted imidazo[4,5b]pyridines. A new synthesis of 1-deazaguanine. Recueil: J. Royal Netherlands Chem. Soc. 1974, 93, 160–162.

[ref32] Kojima N.; Minakawa N.; Matsuda A. Nucleosides and Nucleotides. Part 207: Studies in the Chemical Conversion of the 4-Carboxamide Group of 5-Amino-1-β-d-ribofuranosylimidazole-4-carboxamide (AICA-Riboside). Application for the Synthesis of 1-Deazaguanosine. Tetrahedron 2000, 56, 7909–7914. 10.1016/S0040-4020(00)00712-2.

[ref33] Wolter M.; Nordmann G.; Job G. E.; Buchwald S. L. Copper-catalyzed coupling of aryl iodides with aliphatic alcohols. Org. Lett. 2002, 4, 973–976. 10.1021/ol025548k. 11893199

[ref34] Deghati P.; Bieräugel H.; Wanner M. J.; Koomen G.-J. Mild and regioselective nitration of 1-deazapurine nucleosides using TBAN/TFAA. Tetrahedron Lett. 2000, 41, 1569–1573. 10.1016/S0040-4039(99)02073-0.

[ref35] Deghati P.; Wanner M. J.; Koomen G.-J. Regioselective nitration of purine nucleosides: synthesis of 2-nitroadenosine and 2-nitroinosine. Tetrahedron Lett. 2000, 41, 1291–1295. 10.1016/S0040-4039(99)02271-6.

[ref36] Wanner M. J.; Rodenko B.; Koch M.; Koomen G.-J. New (1-deaza)purine derivatives via efficient C-2 nitration of the (1-deaza)purine ring. Nucl., Nucl., Nucl. Acids 2004, 23, 1313–1320. 10.1081/NCN-200027566. 15571251

[ref37] Orlandi M.; Tosi F.; Bonsignore M.; Benaglia M. Metal-Free Reduction of Aromatic and Aliphatic Nitro Compounds to Amines: A HSiCl_3_-Mediated Reaction of Wide General Applicability. Org. Lett. 2015, 17, 3941–3943. 10.1021/acs.orglett.5b01698. 26262554

[ref38] Serebryany V.; Beigelman L. An efficient preparation of protected ribonucleosides for phosphoramidite RNA synthesis. Tetrahedron Lett. 2002, 43, 1983–1985. 10.1016/S0040-4039(02)00181-8.

[ref39] Serebryany V.; Beigelman L. Synthesis of 2′-*O*-Substituted Ribonucleosides. Nucl. Nucl. Nucl. Acids 2003, 22, 1007–1009. 10.1081/NCN-120022724. 14565332

[ref40] Pitsch S.; Weiss P. A.; Jenny J.; Stutz A.; Wu X. Reliable Chemical Synthesis of Oligoribonucleotides (RNA) with 2′-O-[(Triisopropylsilyl)oxy]methyl (2′-O-tom)-Protected Phosphoramidites. Helv. Chim. Acta 2001, 84, 3773–3795. 10.1002/1522-2675(20011219)84:12<3773::AID-HLCA3773>3.0.CO;2-E.

[ref41] Wachowius F.; Höbartner C. Chemical RNA modifications for studies of RNA structure and dynamics. ChemBioChem 2010, 11, 469–480. 10.1002/cbic.200900697. 20135663

[ref42] Saenger W. Principles of Nucleic Acid Structure; Springer: Berlin, 1984.

[ref43] Majlessi M.; Becker M. M. Formation of the double helix: a mutational study. Nucleic Acids Res. 2008, 36, 2981–2989. 10.1093/nar/gkn134. 18388130 PMC2396424

[ref44] Marky L. A.; Breslauer K. J. Calculating thermodynamic data for transitions of any molecularity from equilibrium melting curves. Biopolymers 1987, 26, 1601–1620. 10.1002/bip.360260911. 3663875

[ref45] Petersheim M.; Turner D. H. Base-stacking and base-pairing contributions to helix stability: thermodynamics of double-helix formation with CCGG, CCGGp, CCGGAp, ACCGGp, CCGGUp, and ACCGGUp. Biochemistry 1983, 22, 256–263. 10.1021/bi00271a004. 6824629

[ref46] Verdolino V.; Cammi R.; Munk B. H.; Bernhard Schlegel H. Calculation of pKa values of nucleobases and the guanine oxidation products guanidinohydantoin and spiroiminodihydantoin using density functional theory and a polarizable continuum model. J. Phys. Chem. B 2008, 112, 16860–16873. 10.1021/jp8068877. 19049279

[ref47] Olieric V.; Rieder U.; Lang K.; Serganov A.; Schulze-Briese C.; Micura R.; Dumas P.; Ennifar E. A fast selenium derivatization strategy for crystallization and phasing of RNA structures. RNA 2009, 15, 707–715. 10.1261/rna.1499309. 19228585 PMC2661828

[ref48] Correll C. C.; Wool I. G.; Munishkin A. The two faces of the Escherichia coli 23 S rRNA sarcin/ricin domain: the structure at 1.11 Å resolution. J. Mol. Biol. 1999, 292, 275–287. 10.1006/jmbi.1999.3072. 10493875

[ref49] Rangadurai A.; Zhou H.; Merriman D. K.; Meiser N.; Liu B.; Shi H.; Szymanski E. S.; Al-Hashimi H. M. Why are Hoogsteen base pairs energetically disfavored in A-RNA compared to B-DNA?. Nucleic Acids Res. 2018, 46, 11099–11114. 10.1093/nar/gky885. 30285154 PMC6237737

[ref50] Kath-Schorr S.; Wilson T. J.; Li N. S.; Lu J.; Piccirilli J. A.; Lilley D. M. J. General acid-base catalysis mediated by nucleobases in the hairpin ribozyme. J. Am. Chem. Soc. 2012, 134, 16717–16724. 10.1021/ja3067429. 22958171 PMC3707309

[ref51] Roth A.; Weinberg Z.; Chen A. G.; Kim P. B.; Ames T. D.; Breaker R. R. A widespread self-cleaving ribozyme class is revealed by bioinformatics. Nat. Chem. Biol. 2014, 10, 56–60. 10.1038/nchembio.1386. 24240507 PMC3867598

[ref52] Gebetsberger J.; Micura R. Unwinding the twister ribozyme: from structure to mechanism. Wiley Interdiscip. Rev. RNA 2017, 8, e1402 10.1002/wrna.1402. PMC5408937 27863022

[ref53] Weinberg Z.; Kim P. B.; Chen T. H.; Li S.; Harris K. A.; Lünse C. E.; Breaker R. R. New classes of self-cleaving ribozymes revealed by comparative genomics analysis. Nat. Chem. Biol. 2015, 11, 606–610. 10.1038/nchembio.1846. 26167874 PMC4509812

[ref54] Ren A.; Košutić M.; Rajashankar K. R.; Frener M.; Santner T.; Westhof E.; Micura R.; Patel D. J. In-line alignment and Mg^2+^ coordination at the cleavage site of the *env22* twister ribozyme. Nat. Commun. 2014, 5, 5534 10.1038/ncomms6534. 25410397 PMC4373348

[ref55] Liu Y.; Wilson T. J.; McPhee S. A.; Lilley D. M. J. Crystal structure and mechanistic investigation of the twister ribozyme. Nat. Chem. Biol. 2014, 10, 739–744. 10.1038/nchembio.1587. 25038788

[ref56] Wilson T. J.; Liu Y.; Domnick C.; Kath-Schorr S.; Lilley D. M. J. The Novel Chemical Mechanism of the Twister Ribozyme. J. Am. Chem. Soc. 2016, 138, 6151–6162. 10.1021/jacs.5b11791. 27153229

[ref57] Gaines C. S.; York D. M. Ribozyme Catalysis with a Twist: Active State of the Twister Ribozyme in Solution Predicted from Molecular Simulation. J. Am. Chem. Soc. 2016, 138, 3058–3065. 10.1021/jacs.5b12061. 26859432 PMC4904722

[ref58] Ucisik M. N.; Bevilacqua P. C.; Hammes-Schiffer S. Molecular Dynamics Study of Twister Ribozyme: Role of Mg(2+) Ions and the Hydrogen-Bonding Network in the Active Site. Biochemistry 2016, 55, 3834–3846. 10.1021/acs.biochem.6b00203. 27295275 PMC5127262

[ref59] Vusurovic N.; Altman R. B.; Terry D. S.; Micura R.; Blanchard S. C. Pseudoknot Formation Seeds the Twister Ribozyme Cleavage Reaction Coordinate. J. Am. Chem. Soc. 2017, 139, 8186–8193. 10.1021/jacs.7b01549. 28598157 PMC5697751

[ref60] Eiler D.; Wang J.; Steitz T. A. Structural basis for the fast self-cleavage reaction catalyzed by the twister ribozyme. Proc. Natl. Acad. Sci. U. S. A. 2014, 111, 13028–13033. 10.1073/pnas.1414571111. 25157168 PMC4246988

[ref61] Breaker R. R.; Emilsson G. M.; Lazarev D.; Nakamura S.; Puskarz I. J.; Roth A.; Sudarsan N. A common speed limit for RNA-cleaving ribozymes and deoxyribozymes. RNA 2003, 9, 949–957. 10.1261/rna.5670703. 12869706 PMC1370461

[ref62] Bevilacqua P. C.; Harris M. E.; Piccirilli J. A.; Gaines C.; Ganguly A.; Kostenbader K.; Ekesan Ş.; York D. M. An Ontology for Facilitating Discussion of Catalytic Strategies of RNA-Cleaving Enzymes. ACS Chem. Biol. 2019, 14, 1068–1076. 10.1021/acschembio.9b00202. 31095369 PMC6661149

[ref63] Jiang H.; Gao Y.; Zhang L.; Chen D.; Gan J.; Murchie A. I. H. The identification and characterization of a selected SAM-dependent methyltransferase ribozyme that is present in natural sequences. Nat. Catal. 2021, 4, 872–881. 10.1038/s41929-021-00685-z.

