# Supplementary material for: 1-Deazaguanosine-Modified RNA: The Missing Piece for Functional RNA Atomic Mutagenesis
Source: J Am Chem Soc. 2022 Jun 6;144(23):10344–52. doi: 10.1021/jacs.2c01877 (PMC9204769; doi:10.1021/jacs.2c01877)
Supplement: Supplementary file 1 — ja2c01877_si_001.pdf [file ja2c01877_si_001.pdf]

Supporting Information

1-Deazaguanosine modified RNA – The missing piece for functional RNA atomic mutagenesis

Raphael Bereiter, Eva Renard, Kathrin Breuker, Christoph Kreutz, Eric Ennifar and Ronald Micura

Contents

Supporting Methods

General procedures S02

Synthesis of 1-deazaguanosine **6** S05

Synthesis of 1-deazaguanosine phosphoramidite **12** S26

Supporting Tables

Supporting Table 1 S44

Supporting Table 2 S45

Supporting Figures

Supporting Figure 1 S46

Supporting Figure 2 S47

Supporting Figure 3 S48

Supporting Figure 4 S49

Supporting Figure 5 S50

Supporting Figure 6 S51

Supporting Figure 7 S52

Supporting Figure 8 S53

Supporting Figure 9 S54

Supporting Figure 10 S55

Supporting Figure 11 S56

Supporting Figure 12 S57

Supporting Figure 13 S58

Supporting Figure 14 S59

Supporting Figure 15 S60

Supporting Figure 16 S61

General procedures

Materials

Reagents were purchased in the highest available quality from commercial suppliers (Merck / Sigma-Aldrich, ABCR, VWR, ChemGenes, CarboSynth, Synthonix) and used without further purification. All reactions were carried out under argon atmosphere, unless otherwise noted. Analytical thin-layer chromatography (TLC) was performed on Macherey-Nagel Polygram® SIL G/UV_{254} plates. 0.2 mm Silica gel 60 for column chromatography was purchased from Macherey-Nagel.

NMR measurements of compounds

^{1}H, ^{13}C, and ^{31}P spectra were recorded on a Bruker Ultrashield™ 400 Plus spectrometer. Chemical shifts (δ) are reported relative to tetramethylsilane (TMS), referenced to the residual solvent signal (DMSO-d_6: 2.50 ppm for ^{1}H and 39.52 ppm for ^{13}C spectra; $CDCl_3$: 7.26 ppm for ^{1}H and 77.16 ppm for ^{13}C spectra). The following abbreviations were used to denote multiplicities: s = singlet, d = doublet, t = triplet, q = quadruplet, m = multiplet, b = broad. Signal assignments are based on ^{1}H-^{1}H-COSY, ^{1}H-^{13}C-HSQC and ^{1}H-^{13}C-HMBC experiments.

High-resolution mass spectrometry of compounds

High resolution mass spectra were recorded in positive ion mode on a Thermo Scientific Q Exactive Orbitrap, ionized via electrospray at 3.7 kV spray voltage.

RNA solid-phase synthesis

Standard phosphoramidite chemistry was applied for RNA strand elongation and incorporation of 1-deazaguanosine. 2'-*O*-TOM & acetyl protected nucleoside phosphoramidite building blocks and 2'-*O*-TBDMS 1000 Å CPG solid support (>15nt) were purchased from ChemGenes, Primer support™ 5G (<15nt) was purchased from GE Healthcare. All oligonucleotides were synthesized on a ABI 392 Nucleic Acid Synthesizer following standard methods: detritylation (90 sec) with dichloroacetic acid/1,2-dichloroethane (4/96); coupling (5.0 min) with phosphoramidites/acetonitrile (100 mM, 200 µL) and benzylthiotetrazole / acetonitrile (300 mM, 500 µL); capping (2 x 25 sec) with Cap A mild / Cap B mild (1/1 v/v), Cap A mild: phenoxyacetic anhydride/acetonitrile (100 mM), Cap B mild: *N*-methylimidazole / *sym*-collidine / tetrahydrofuran (0.160/0.265/10 v/v/v); oxidation (60 sec) with iodine (20 mM) in tetrahydrofuran/pyridine/H_2O (35/10/5 v/v/v). Phosphoramidites were diluted to a concentration of 0.1 mol/l and dried over activated molecular sieves (3 Å) overnight.

Deprotection, purification and quantification of unmodified and modified RNA

For basic deprotection of unmodified and c^1G modified RNA, the solid support was mixed with aqueous methylamine (40 %, 0.65 mL) and aqueous ammonia (28 %, 0.65 mL) for 15 minutes at 65 °C or 3 hours at 37 °C. The supernatant was removed and the solid support was washed twice with 0.5 mL tetrahydrofuran/H_2O (1/1). Combined supernatant and washings were evaporated to dryness and the residue was dissolved in a solution of tetra-*n*-butylammonium-fluoride in tetrahydrofuran (1.0 M, 1.5 mL) and incubated for 14 hours at 37 °C, for the removal of 2'-*O*-silyl protecting groups. The reaction was quenched by the addition of aqueous

tetraethylammonium acetate solution (1.0 M, 1.5 mL, pH 7.4). Tetrahydrofuran was removed under reduced pressure and the sample was desalted with size-exclusion column chromatography (GE Healthcare, HiPrep™ 26/10 Desalting; Sephadex G25) eluting with H_2O; collected fractions were evaporated and the RNA dissolved in H_2O (1 mL). The crude RNA was purified by anion exchange chromatography on a GE Healthcare Äkta Explorer HPLC System containing a semipreparative Dionex DNAPac™ PA-100 column (9 mm x 250 mm) at 60 or 80 °C, a pressure between 1.7 and 2.0 MPa and a flow rate of 1 mL/min (Eluent A: 25 mM Tris·HCl, 0.01 M $NaClO_4$, 20% acetonitrile, pH 8.0; Eluent B: 25 mM Tris·HCl, 0.6 M $NaClO_4$, 20 % acetonitrile, pH 8.0). Fractions containing RNA were diluted with 0.1 M triethylammonium bicarbonate solution, loaded on a C18 SepPak Plus® cartridge (Waters/Millipore), washed with H_2O and eluted with acetonitrile/H_2O (1/1). Crude and purified RNA were analyzed by anion exchange chromatography on a GE Healthcare äkta explorer HPLC System containing a Dionex DNAPac™ PA-100 column (4 mm x 250 mm) at 60 or 80 °C with a flow rate of 1 mL/min unless otherwise noted. For RNA shorter or equal to 15 nucleotides, a gradient of 0 – 40 % B in 30 minutes and for RNA longer than 15 nucleotides a gradient of 0 – 60 % B in 45 minutes was used; Eluent A: 25 mM Tris·HCl, 0.01 M $NaClO_4$, 20% acetonitrile, pH 8.0; Eluent B: 25 mM Tris·HCl, 0.6 M $NaClO_4$, 20 % acetonitrile, pH 8.0 HPLC traces were recorded at UV absorption by 260 nm. RNA quantification was performed on an Implen P300 Nanophotometer.

Mass spectrometry of unmodified and modified RNA

RNA samples (3 µL) were diluted with 40 mM Na_2H_2(EDTA)/H_2O (5/4) for a total volume of 30 µL, injected onto a C18 XBridge 2.5 µm (2.1 mm x 50 mm) column at a flow rate of 0.1 mL/min and eluted with 0 - 100 % B gradient at 30 °C (Eluent A: 8.6 mM triethylamine, 100 mM 1,1,1,3,3,3-hexafluoroisopropanol in H_2O; Eluent B: methanol). RNA traces were analyzed on a Finnigan LCQ Advantage Max electrospray ionization mass spectrometer with 4.0 kV spray voltage in negative mode.

Melting Curve measurements of unmodified and modified RNA

RNA samples were lyophilized as triethylammonium salts, dissolved in 800 or 330 µL Buffer (10 mM Na_2HPO_4 containing 150 mM NaCl at pH 7.0) and transferred into UV permeable high precision cells made of quartz SUPRASIL® with a light path of 10 mm or 1 mm. UV melting profiles were recorded at 250 and 260 nm on Varian Cary 100 or Agilent Cary 3500 UV-Vis spectrophotometers equipped with multiple cell holders and peltier temperature control devices. Each RNA was measured at five different concentrations (between ~1 and ~100 µM) and with at least four ramps (heating-cooling-heating-cooling; 1°C min^{-1} heating/cooling rate). T_m values were determined by calculating the first derivative, usually from data of the third ramp (heating).

NMR experiments

RNA samples were lyophilized as sodium salts, dissolved in 500 µL NMR buffer (25 mM NaCl, 10% D_2O, pH 5.8) and transferred into 5 mm NMR tubes. Sample concentrations varied

between 0.2 and 0.3 mM. All NMR experiments were conducted on a Bruker 600 MHz Avance II+ NMR or a 700 MHz Avance Neo NMR both equipped with a Prodigy TCI probe.

pK_a determination by UV-spectroscopy

1-Deazaguanine was dissolved in 800 µL buffer (25 µM citric acid for pK_a(1) and 25 µM tris-(hydroxymethyl)-aminomethane (TRIS) for pK_a(2), 100 mM KCl) to reach a final concentration of 95 µM. The mixture was transferred into UV permeable high precision cells made of quartz SUPRASIL® with a light path of 10 mm. pH depending absorption profiles were recorded from 400 nm to 200 nm on a Varian Cary-100 scans spectrophotometer equipped with a multiple cell holder and a peltier temperature control device.

Ribozyme cleavage assays

The ribozyme and substrate strands (2.42 nmol each) were lyophilized as triethylammonium salts and dissolved in 33 µL H_2O. The dissolved RNA strands were heated to 90 °C for 2 minutes and subsequently cooled to room temperature. 3 µL of this solution were used for time point zero. The remaining was mixed with 6 µL of 200 mM HEPES pH 7.5, 2 µL KCl 2 M and 2 µL $MgCl_2$ 40 mM to reach a final concentration of 55 µM of each RNA strand, 30 mM HEPES, 100 mM KCl and 2 mM Mg^{2+}. Cleavage reaction was initiated by the addition of $MgCl_2$ solution. Samples were drawn after the indicated time points and quenched by the addition of equal amounts of 40 mM Na_2H_2EDTA and diluted to 100 µl. We assayed the ribozyme under single turnover conditions for reasons of comparison to our previous studies on the twister ribozyme.

Crystallization and structure solution of c^{1}G modified RNA

A chemically-synthesized 27-nucleotide RNA corresponding to the Escherichia coli 23S rRNA sarcin/ricin loop (SRL), modified with c^{1}G at position 2655 was used for crystallization trials. RNA was dissolved in a buffer made with Na_2H_2EDTA pH 8.0 1 mM, Tris-HCl pH 8.0 10 mM at a 350 µM concentration. The RNA sample was heated at 55 °C for 10 min and cooled at 25 °C by switching off the heating block. Crystals were grown at 20 °C by mixing 2 µl of RNA sample with 1 µl of a crystallization buffer made with ammonium sulfate 3.2 M, magnesium chloride 10 mM, manganese chloride 10 mM and potassium 3-(*N*-morpholino) propanesulfonic acid (MOPS) pH 7.0 50 mM. Prior to data collection, crystals were cryoprotected for about 5 min in a reservoir solution containing 15% of glycerol, and 3.4 M ammonium sulfate, 10 mM magnesium chloride, 10 mM manganese chloride and 50 mM potassium MOPS pH 7.0, and flash-frozen in liquid ethane. Crystals were subsequently transferred into liquid nitrogen for automatic mounting. X-ray diffraction data were collected on the X06SA beamline at the SLS synchrotron. Data were processed with the XDS Package and the structure was solved by molecular replacement with MOLREP using an unmodified SRL RNA as a search model (PDB ID 3DVZ). The structure was refined with the PHENIX package. The model was built using Coot. Coordinates have been deposited with the PDB database (entry number 7QP2).

Synthesis of 1-deazaguanosine (6)

6-Iodo-1-deazapurine (1)

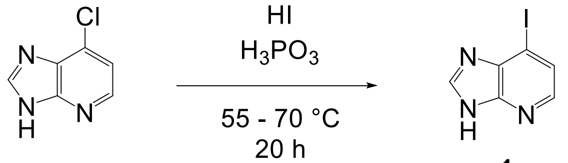

1

6-Chloro-1-deazapurine (3.15 g, 20.51 mmol) and phosphorous acid (H_3PO_3, 840.94 mg, 10.26 mmol) were suspended in 57 % aqueous hydroiodic acid (46.03 g, 27.08 mL, 205.12 mmol) and stirred at 55 °C for 18 hours followed by stirring at 70 °C for 2 hours. The resulting suspension was filtered of and washed with large quantities of ice cold water. The yellow solid was dried under high vacuum. Yield: 5.00 g of compound **1** as a yellow powdery solid (99 %). $[M+H]^+$ calcd.: 245.95; found: 245.95. ^{1}H-NMR: (400 MHz, DMSO-d$_6$, 25 °C): δ = 7.97 (1H, d, J=5.25 Hz, ***H***-C(1)), 8.24 (1H, d, J=5.25 Hz, ***H***-C(2)), 9.23 (1H, s, ***H***-C(8)), 13.78 (2H, b, ***H***-N(7&9)). ^{13}C-NMR: (400 MHz, DMSO-d$_6$, 25 °C): δ = 96.20 (***C***(6)), 128.65 (***C***(1)), 133.08 (***C***(5)), 144.18 (***C***(2)), 144.59 (***C***(8)), 145.28 (***C***(4)).

^{1}H-NMR (400 MHz, DMSO-d_6, 25 °C) of compound **1**

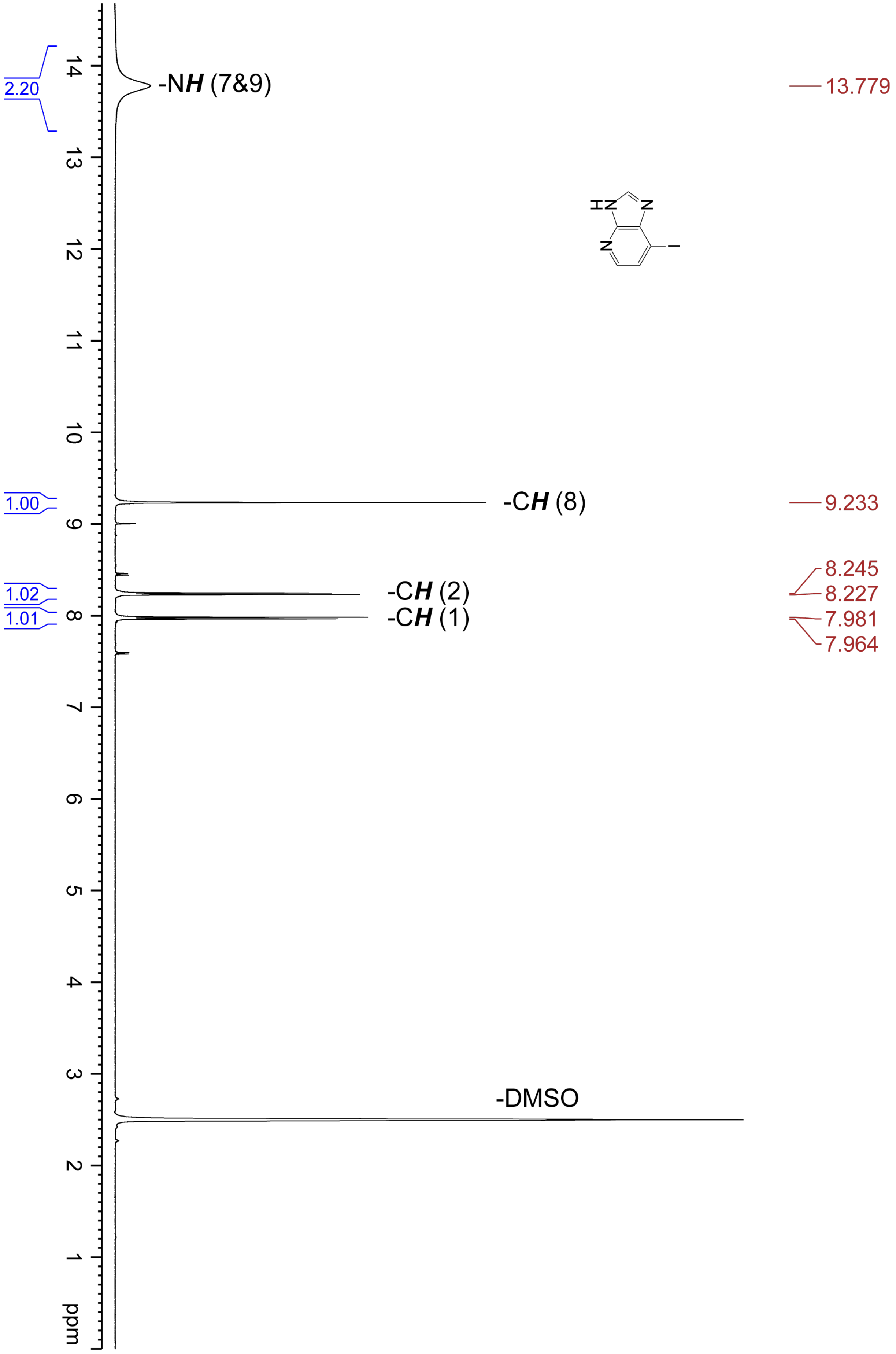

^{13}C-NMR (100 MHz, DMSO-d_6, 25 °C) of compound **1**

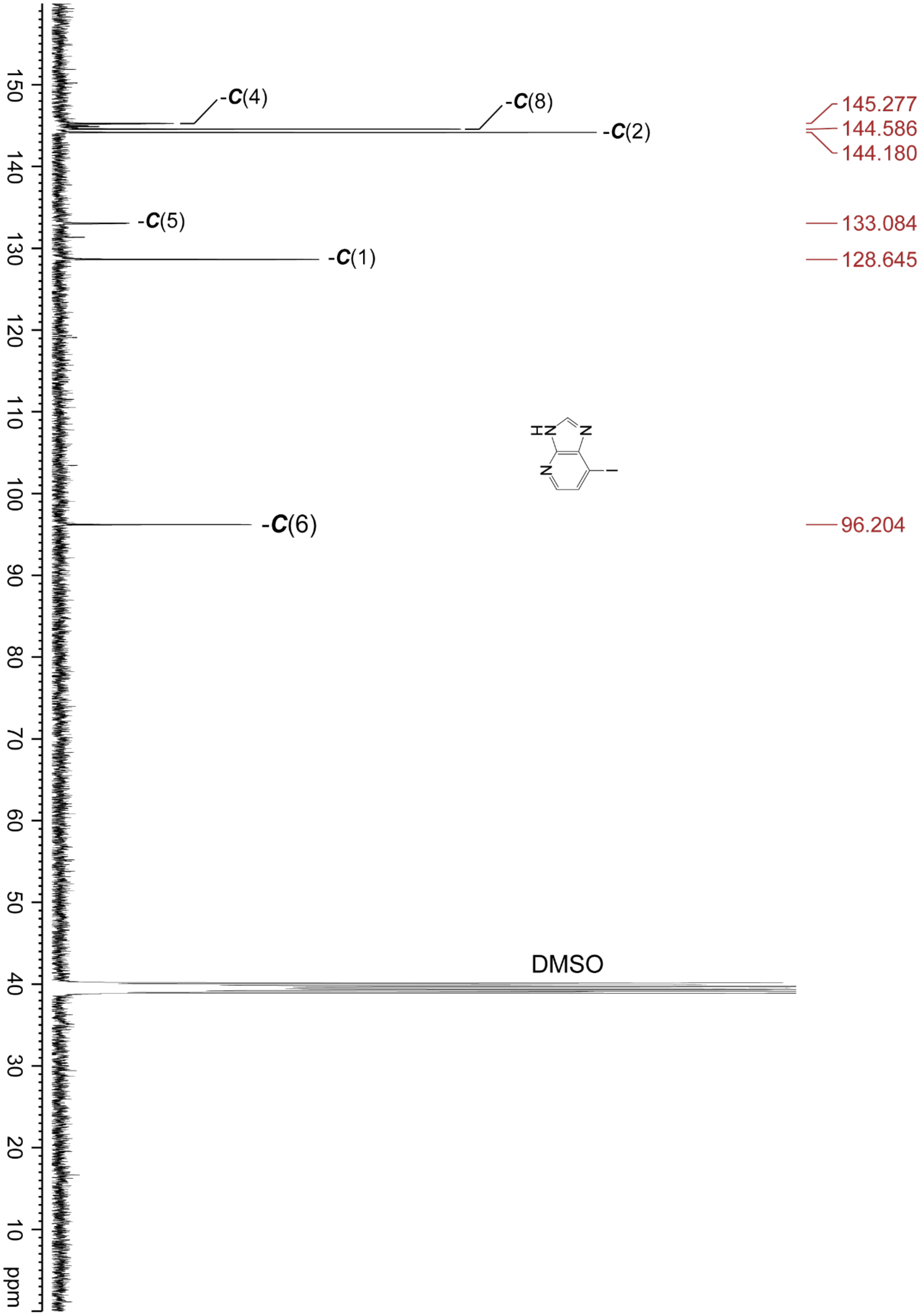

6-Iodo-9-(2',3',5'-tri-*O*-acetyl-*ß*-D-ribofuranosyl)-1-deazapurine (2)

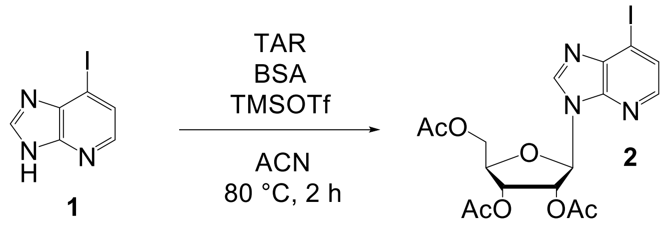

Compound **1** (5.03 g, 20.53 mmol) and *N*,*O*-bis-(trimethylsilyl)-acetamide (BSA, 4.59 g, 5.52 mL, 22.58 mmol) were suspended in 150 mL dry acetonitrile. The mixture was heated up to 80 °C for 15 minutes, continued by cooling the reaction to 0 °C. At this temperature, 1,2,3,5-tetra-*O*-acetyl-*β*-D-ribofuranose (TAR, 7.19 g, 22.58 mmol) and trimethylsilyl trifluoromethane-sulfonate (TMSOTf, 5.02 g, 4.08 mL, 22.58 mmol) were added and the reaction mixture was stirred at 80 °C for 2 hours, whereby the mixture turned into a dark solution. After complete consumption of the starting material, the solvent was evaporated and the residue was dissolved in dichloromethane, extracted twice with saturated sodium bicarbonate solution and finally with brine. The organic layer was dried over Na_2SO_4, filtered and evaporated to dryness. The crude product was purified with silica gel chromatography using 5 to 15 % acetone in dichloromethane as gradient. Yield: 5.80 g of compound **2** as a slightly yellow foam (56%). TLC: (CH_2Cl_2 / acetone, 88:12): R_f = 0.5. ESI-MS (m/z): $[M+H]^+$ calcd.: 504.03; found: 504.03. ^{1}H-NMR: (400 MHz, $CDCl_3$, 25 °C): δ = 2.07 (3H, s, COC***H₃***); 2.11 (3H, s, COC***H₃***); 2.14 (3H, s, COC***H₃***); 4.37 (1H, m, ***H(a)***-C(5')); 4.45 (2H, m, ***H(b)***-C(5') & ***H***-C(4')); 5.69 (1H, t, J=5.00 Hz, ***H***-C(3')), 6.02 (1H, t, J=5.36 Hz, ***H***-C(2')), 6.23 (1H, d, J=5.24 Hz, ***H***-C(1')); 7.72 (1H, d, J=5.08 Hz, ***H***-C(1)); 8.03 (1H, d, J=5.12 Hz, ***H***-C(2)); 8.27 (1H, s, ***H***-C(8)). ^{13}C-NMR: (400 MHz, $CDCl_3$, 25 °C): δ = 63.20 (***C***H₃-acetyl); 70.79 (***C***H₃-acetyl); 73.06 (***C***H₃-acetyl); 76.84 (***C***(5')); 77.15 (***C***(3')); 77.47 (***C***(2')); 80.37 (***C***(4')); 86.94 (***C***(1')); 99.27 (***C***(6)); 128.80 (***C***(1)); 139.39 (***C***(5)), 142.54 (***C***(8)); 144.33 (***C***(4)), 144.88 (***C***(2)), 169.45 (***C***=O(acetyl)); 169.69 (***C***=O(acetyl)); 170.46 (***C***=O(acetyl)).

^{1}H-NMR (400 MHz, $CDCl_3$, 25 °C) of compound **2**

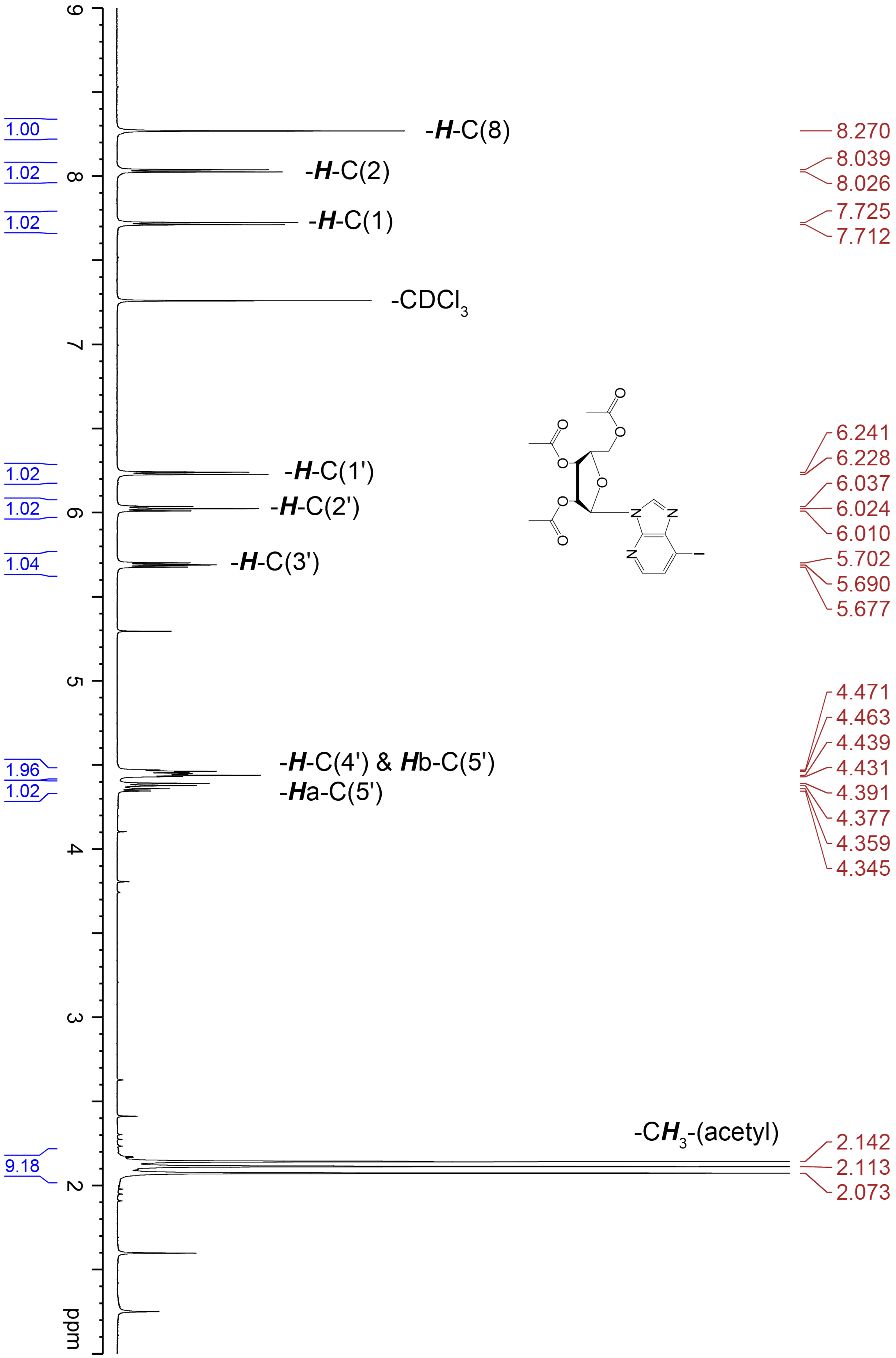

^{13}C-NMR (100 MHz, $CDCl_3$, 25 °C) of compound **2**

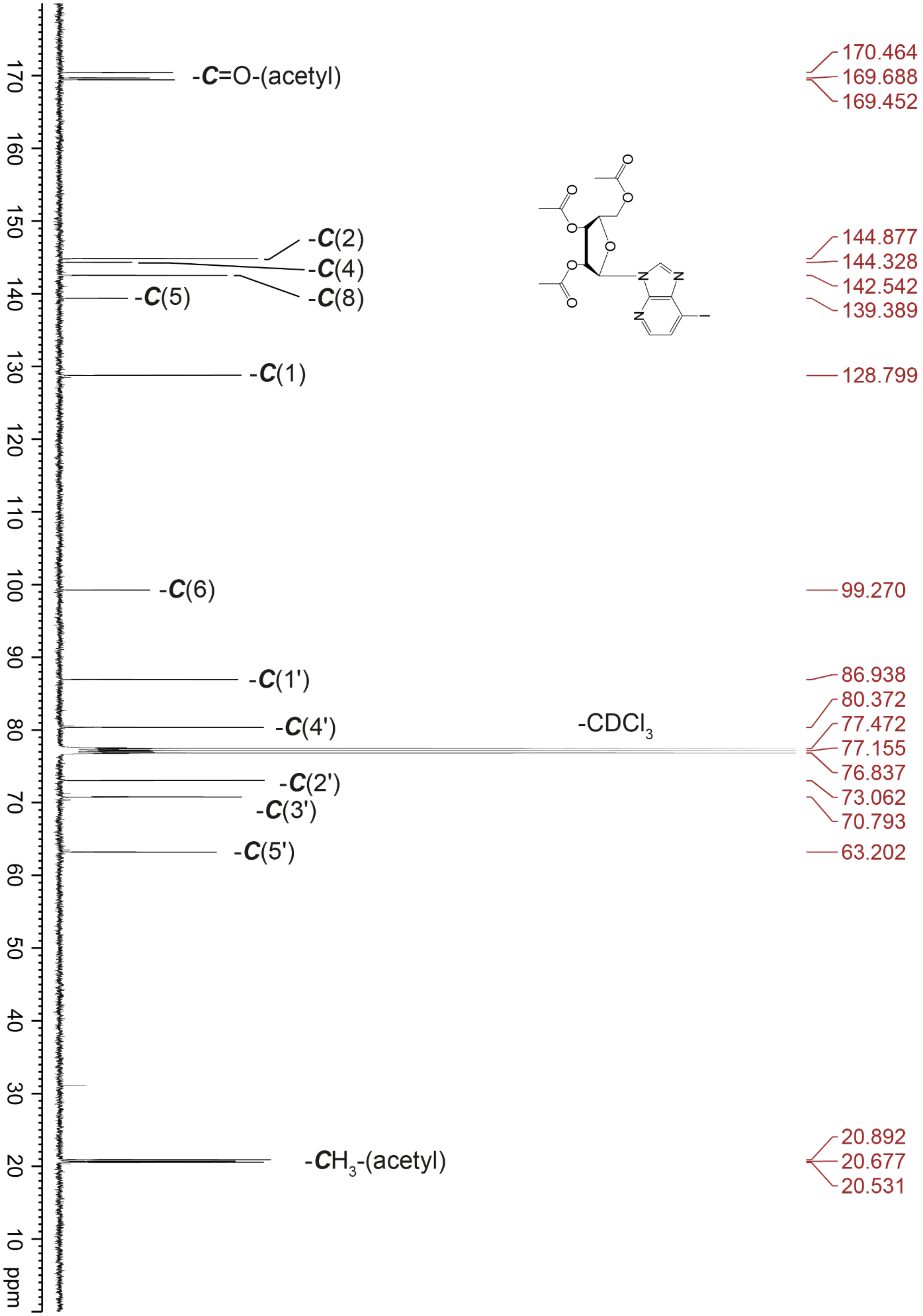

^{1}H, ^{13}C-HMBC NMR (400 MHz, $CDCl_3$, 25 °C) of compound **2**

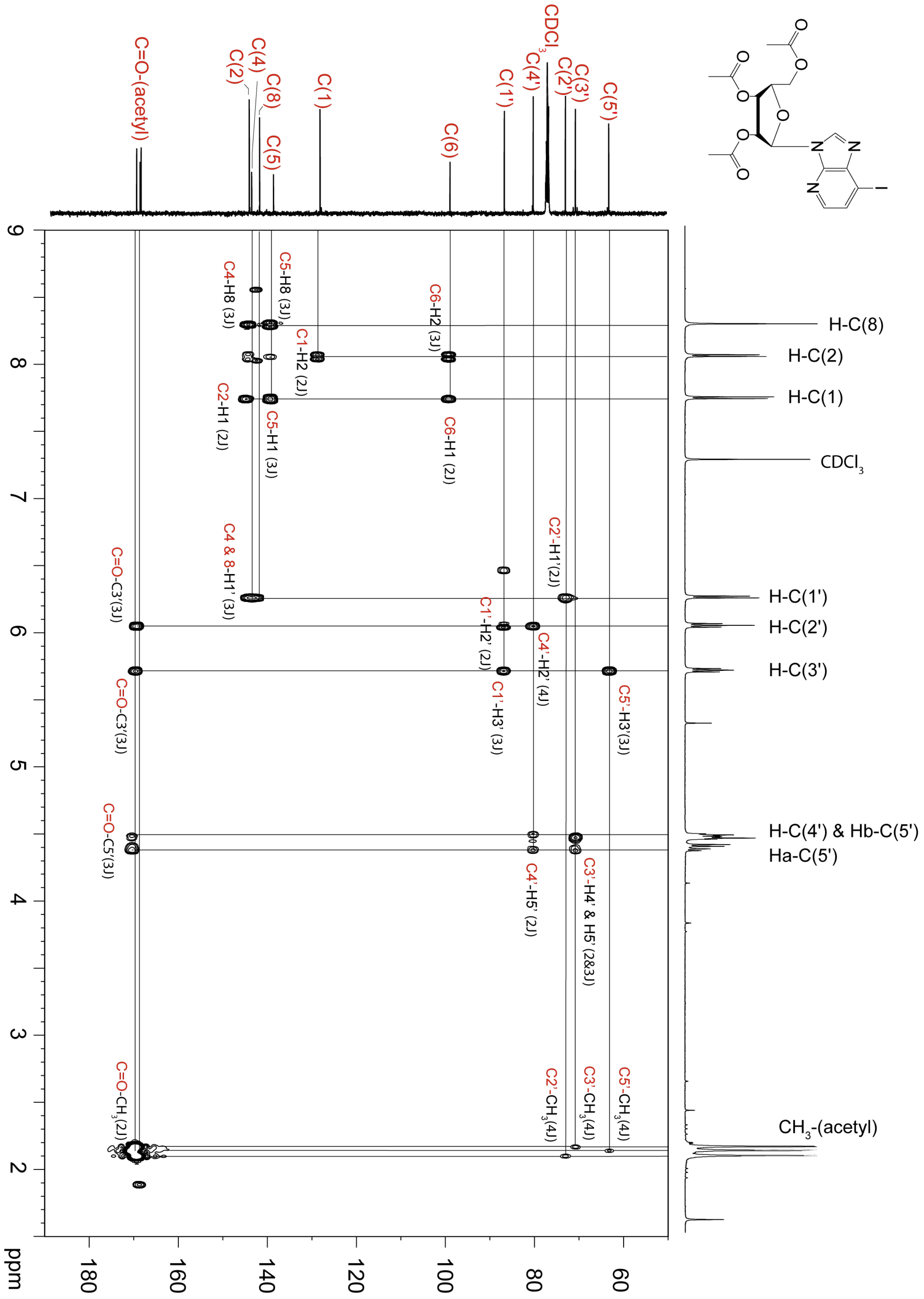

6-Iodo-9-[2',3',5'-*O*-tris(*tert*-butyldimethylsilyl)-*ß*-D-ribofuranosyl]-1-deazapurine (3)

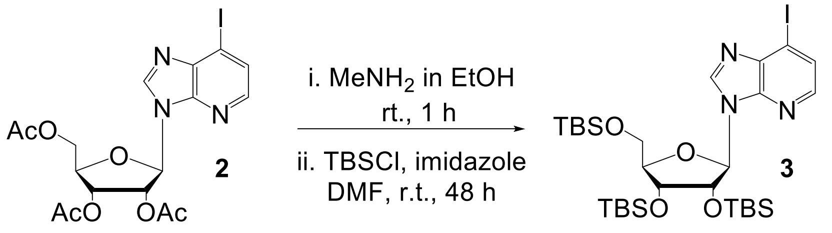

Compound **2** (927 mg, 1.84 mmol) was dissolved in a solution of 33 wt% methylamine in ethanol (4.51 g, 5.96 mL, 47.89 mmol) and stirred at room temperature for one hour. After deprotection of the acetyl moieties (TLC: R_f 0.3 in CH_2Cl_2/MeOH 9/1), the reaction mixture and all volatiles were evaporated and the crude product was dissolved in DMF (10 mL). Subsequently, imidazole (877.82 mg, 12.89 mmol) and *tert*-butyldimethylsilylchlorid (TBSCl, 1.67 g, 11.05 mmol) were added and the mixture was stirred for 48 hours at room temperature. After complete protection of the ribose, the solvent was removed under reduced pressure and the oily residue was dissolved in ethyl acetate. The organic layer was extracted 3 times with brine, dried over Na_2SO_4, filtered and evaporated to dryness. The crude product was purified with silica gel chromatography using 0 to 10 % ethyl acetate in cyclohexane as gradient. Yield: 1.16 g of compound **3** as a white foam (87%). TLC: (cyclohexane / ethyl acetate, 85:15): R_f = 0.42. ESI-MS (m/z): $[M+H]^+$ calcd.: 720.25; found: 720.25. ^{1}H-NMR: (400 MHz, $CDCl_3$, 25 °C): δ = -0.30 (3H, s, Si-C***H₃***); -0.07 (3H, s, Si-C***H₃***); 0.12 (12H, m, Si-C***H₃***); 0.77 (9H, s, Si-C(C***H₃***)₃)); 0.94 (18H, d, J=3.36 Hz, Si-C(C***H₃***)₃)); 3.78 (1H, m, ***H(a)***-C(5')); 4.02 (1H, m, ***H(b)***-C(5')); 4.14 (1H, m, ***H***-C(4')); 4.33 (1H, t, J=3.82 Hz, ***H***-C(3')); 4.75 (1H, t, J=4.90 Hz, ***H***-C(2')); 6.11 (1H, d, J=5.40 Hz, ***H***-C(1')); 7.67 (1H, d, J=5.08 Hz, ***H***-C(1)); 8.00 (1H, d, J=5.08 Hz, ***H***-C(2)); 8.41 (1H, s, ***H***-C(8)). ^{13}C-NMR: (100 MHz, $CDCl_3$, 25 °C): δ = (-5.22) – (-4.27) (-***C***H₃-Si-***C***H₃); 17.99 - 18.70 (Si-C(***C***H₃)₃); 25.81 – 26.26 (Si-***C***(CH₃)₃); 62.79 (***C***(5')); 72.30 (***C***(3')); 75.71 (***C***(2')); 85.71 (***C***(4')); 88.65 (***C***(1')); 98.61 (***C***(6)); 128.25 (***C***(1)); 139.22 (***C***(5)); 143.14 (***C***(8)); 144.38 (***C***(2)); 144.79 (***C***(4)).

^{1}H-NMR (400 MHz, $CDCl_3$, 25 °C) of compound **3**

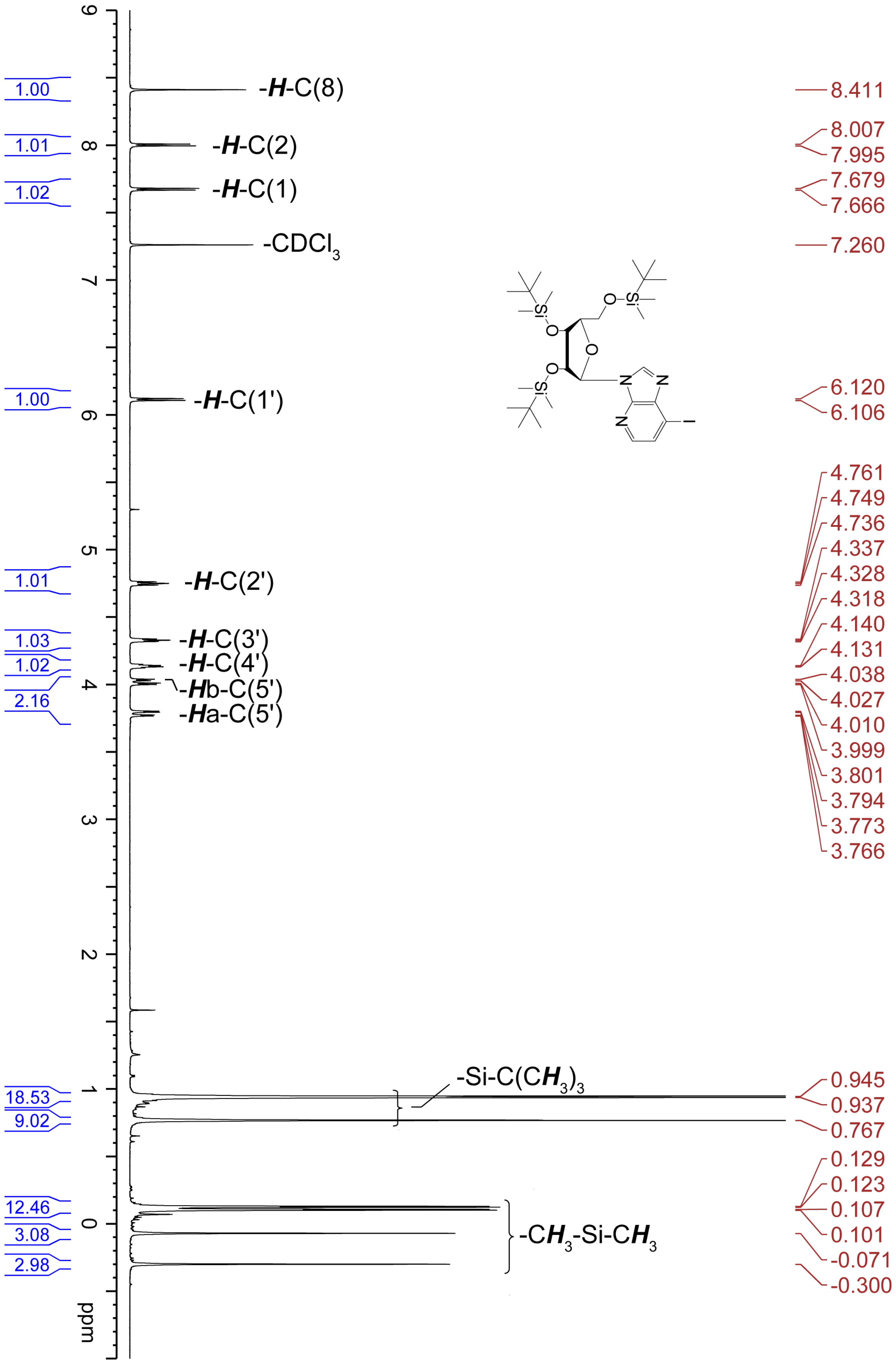

^{13}C-NMR (100 MHz, $CDCl_3$, 25 °C) of compound **3**

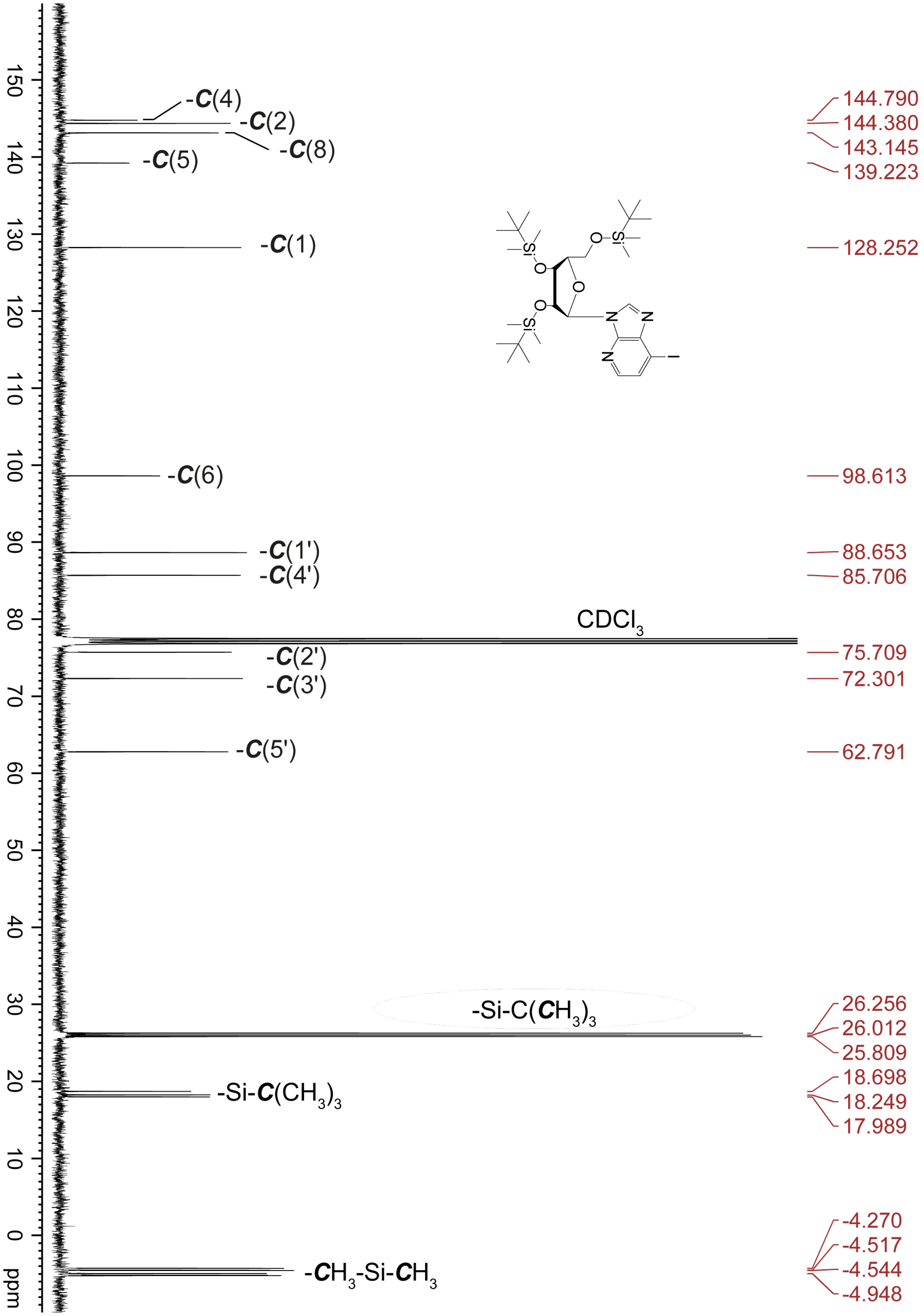

6-Benzoxy-9-[2',3',5'-*O*-tris(*tert*-butyldimethylsilyl)-ß-D-ribofuranosyl]-1-deazapurine (4)

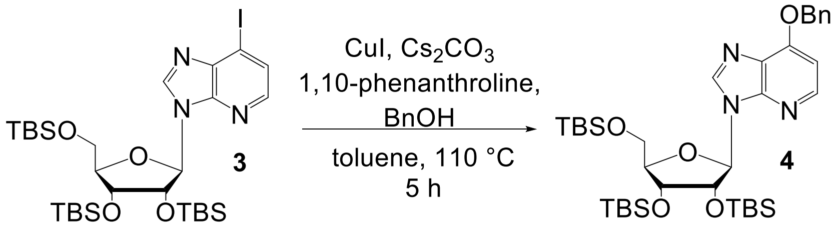

Compound **3** (2.23 g, 3.10 mmol), copper(I)iodide (CuI, 58.99 mg, 0.31 mmol), 1,10-phenanthroline (111.64 mg, 0.62 mmol), caesium carbonate (Cs_2CO_3, 1.41 g, 4.34 mmol) and benzylalcohol (BnOH, 669.9 mg, 632.01 µL, 6.20 mmol) were suspended in 1.55 mL toluene (0.5 mL per 1 mmol compound **3**) and stirred for 5 hours at 110 °C under atmospheric conditions. After complete reaction, the suspension was allowed to cool to room temperature and the catalyst was filtered off. The filtrate was evaporated to dryness and the crude product was purified with silica gel chromatography using 0 to 10 % ethyl acetate in cyclohexane as gradient. Yield: 2.06 g of compound **4** as a colourless oil (95 %). TLC: (cyclohexane / ethyl acetate, 85:15): R_f = 0.53. ESI-MS (m/z): $[M+H]^+$ calcd.: 700.40; found: 700.40. ^{1}H-NMR: (400 MHz, $CDCl_3$, 25 °C): δ = -0.26 (3H, s, Si-C***H₃***); -0.07 (3H, s, Si-C***H₃***); 0.12 (12H, m, Si-C***H₃***); 0.78 (9H, s, Si-C(C***H₃***)₃)); 0.94 (18H, d, J=9.05 Hz, Si-C(C***H₃***)₃)); 3.78 (1H, m, ***H(a)***-C(5')); 4.04 1H, m, ***H(b)***-C(5')); 4.13 (1H, m, ***H***-C(4')); 4.34 (1H, t, J=3.89 Hz, ***H***-C(3')); 4.76 (1H, t, J=4.81 Hz, ***H***-C(2')), 5.53 (2H, s, C***H₂***-benzyl); 6.13 (1H, d, J=5.26 Hz, ***H***-C(1')); 6.71 (1H, d, J=5.60 Hz, ***H***-C(1)); 7.31 - 7.50 (5H, m, C***H***-arom.-(benzyl)); 8.18 (1H, d, J=5.57 Hz, ***H***-C(2)); 8.25 (1H, s, ***H***-C(8)). ^{13}C-NMR: (100 MHz, $CDCl_3$, 25 °C): δ = (-5.22) – (-4.26) (-***C***H₃-Si-***C***H₃); 18.01 - 18.69 (Si-C(***C***H₃)₃); 25.85 - 26.25 (Si-***C***(CH₃)₃); 62.76 (***C***(5')); 71.36 (***C***H₂-benzyl); 72.24 (***C***(3')); 75.63 (***C***(2')); 85.43 (***C***(4')); 88.42 (***C***(1')); 103.72 (***C***(1)); 126.31 (***C***(5)); 127.74 - 128.72 (***C***H-benzyl); 136.39 (***C***-quart.benzyl); 140.96 (***C***(8)); 145.92 (***C***(2)); 148.97 (***C***(4)); 156.92 (***C***(6)).

^{1}H-NMR (400 MHz, $CDCl_3$, 25 °C) of compound **4**

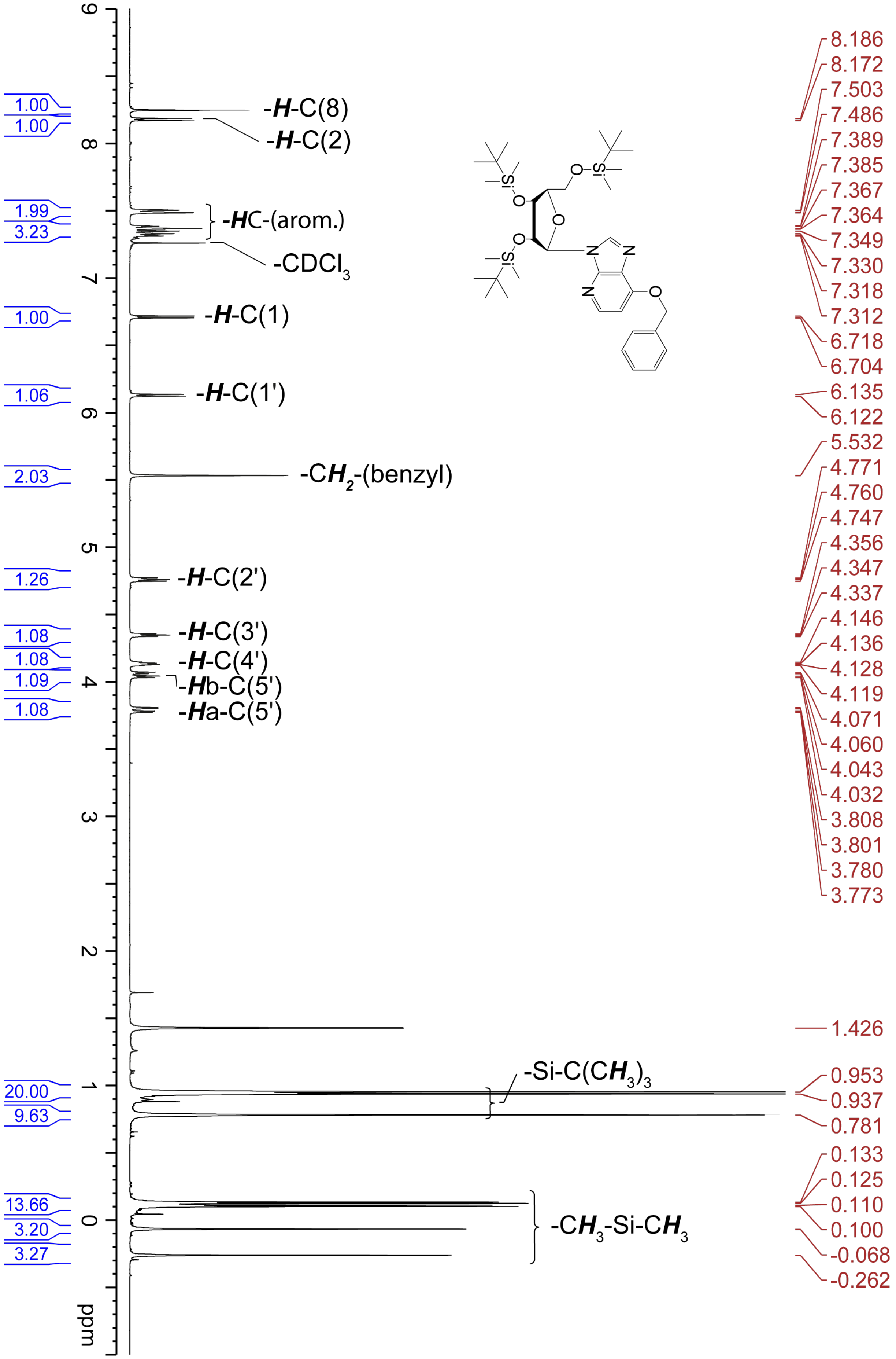

^{13}C-NMR (100 MHz, $CDCl_3$, 25 °C) of compound **4**

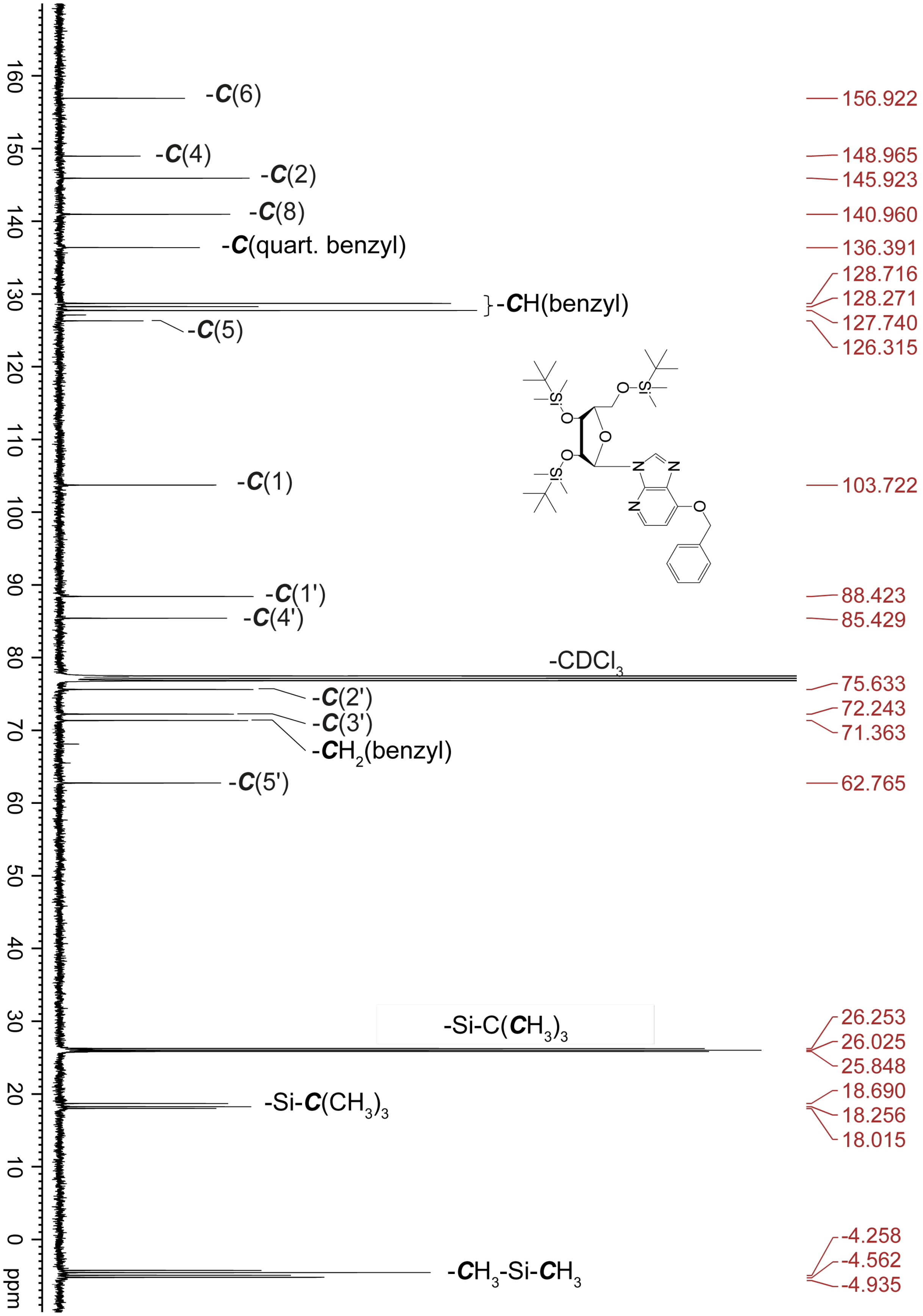

6-Benzoxy-2-nitro-9-[2',3',5'-*O*-tris-(*tert*-butyldimethylsilyl)-ß-D-ribofuranosyl]-1-deazapurine (5)

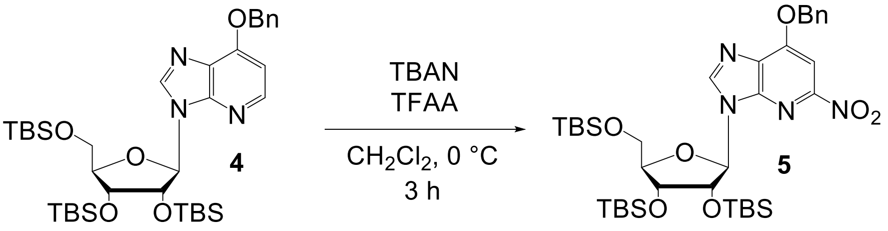

Tetrabutylammonium nitrate (TBAN, 724.06 mg, 2.38 mmol) was dissolved in 7.5 mL dry dichloromethane under argon atmosphere, then, trifluoroacetic anhydride (TFAA, 499.46 mg, 330.77 mL, 2.38 mmol) was added and the mixture was stirred at 0 °C for 10 minutes. Meanwhile, compound **4** (1.11 g, 1.59 mmol) was dissolved in 7.5 mL dry dichloromethane and cooled to 0 °C under argon atmosphere. The nitration mixture was transferred with a syringe and added over a period of 10 minutes to the dissolved substrate. The resulting reaction mixture was stirred for 3 hours at 0 °C under argon atmosphere. Afterwards, the reaction was quenched by adding the whole into a separatory funnel containing saturated sodium bicarbonate solution, followed by vigorously shaking. The organic layer was again washed with saturated sodium bicarbonate solution and brine and was finally dried over Na_2SO_4, filtered and evaporated to dryness. The crude product was purified with silica gel chromatography using 10 to 15 % ethyl acetate in cyclohexane as gradient. Yield: 781 mg of compound **5** as a slightly yellow foam (68%). TLC: (toluene / aceton, 97.5:2.5): R_f = 0.71. ESI-MS (m/z): $[M+H]^+$ calcd.: 745.38; found: 745.38. ^{1}H-NMR: (400 MHz, $CDCl_3$, 25 °C): δ = -0.18 (3H, s, Si-C***H₃***); -0.03 (3H, s, Si-C***H₃***); 0.10-0.17 (12H, m, Si-C***H₃***); 0.81 (9H, s, Si-C(C***H₃***)₃)); 0.95 (18H, d, J=9.05 Hz, Si-C(C***H₃***)₃)); 3.82 (1H, m, ***H(a)***-C(5')); 4.17 1H, m, ***H(b)***-C(5') & ***H***-C(4')); 4.36 (1H, t, J=3.92 Hz, ***H***-C(3')); 4.81 (1H, t, J=4.40 Hz, ***H***-C(2')), 5.67 (2H, s, C***H₂***-benzyl); 6.05 (1H, d, J=4.68 Hz, ***H***-C(1')); 7.35 - 7.53 (5H, m, C***H***-arom.-(benzyl)); 7.80 (1H, d, J=5.60 Hz, ***H***-C(1)); 8.50 (1H, s, ***H***-C(8)). ^{13}C-NMR: (100 MHz, $CDCl_3$, 25 °C): δ = (-5.30) – (-4.25) (-***C***H₃-Si-***C***H₃); 18.02 - 18.66 (Si-C(***C***H₃)₃); 25.82 - 26.24 (Si-***C***(CH₃)₃); 62.33 (***C***(5')); 71.68 (***C***(3')); 72.57 (***C***H₂-benzyl); 74.93 (***C***(2')); 85.42 (***C***(4')); 89.63 (***C***(1')); 99.04 (***C***(1)); 128.06 – 128.93 (***C***H-benzyl); 130.04 (***C***(5)); 135.22 (***C***-quart.benzyl); 145.30 (***C***(8)); 145.83 (***C***(4)); 153.62 (***C***(2)); 158.25 (***C***(6)).

^{1}H-NMR (400 MHz, $CDCl_3$, 25 °C) of compound **5**

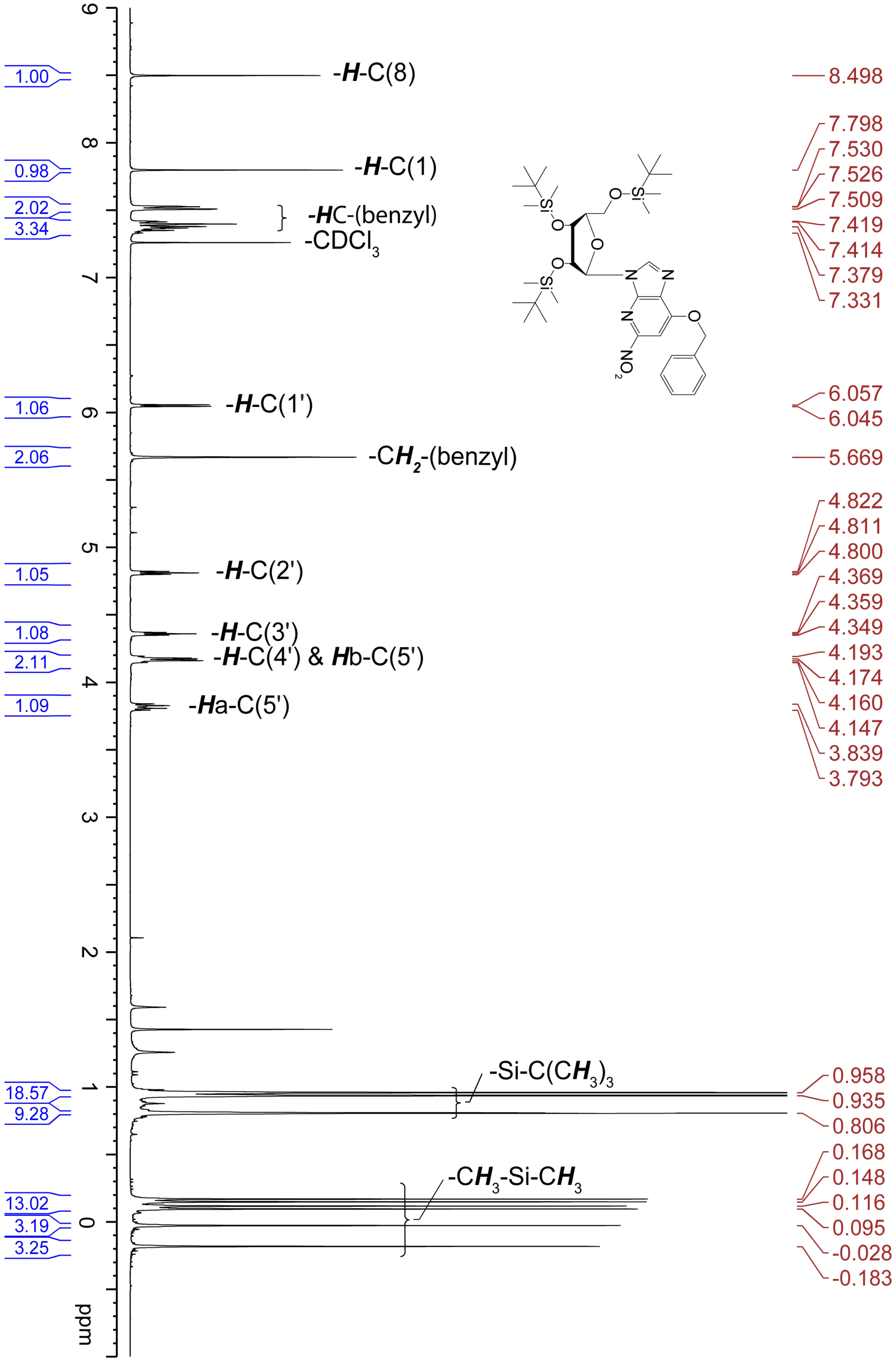

^{13}C-NMR (100 MHz, $CDCl_3$, 25 °C) of compound **5**

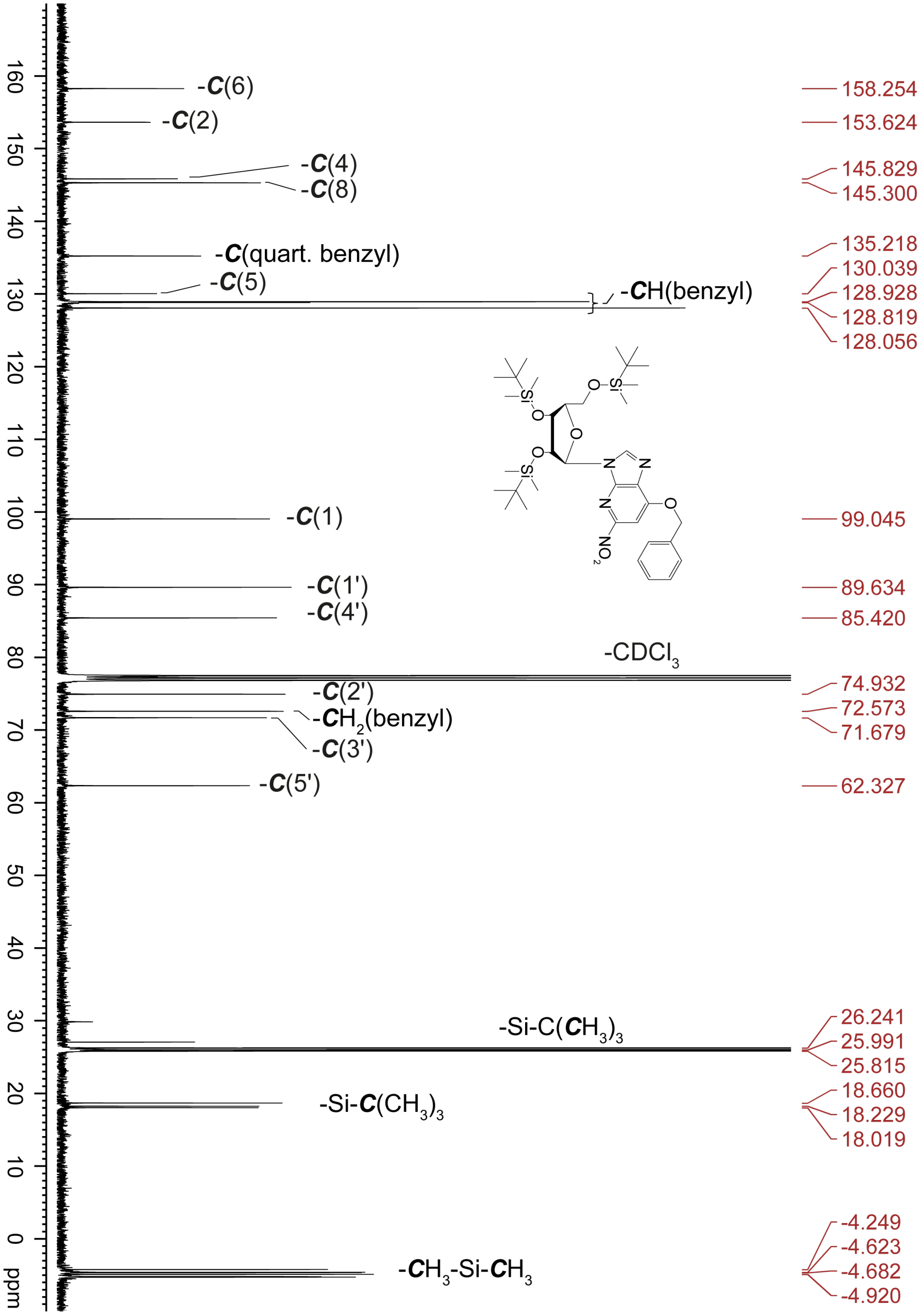

^{1}H, ^{13}C-HMBC NMR (400 MHz, $CDCl_3$, 25 °C) of compound **5**

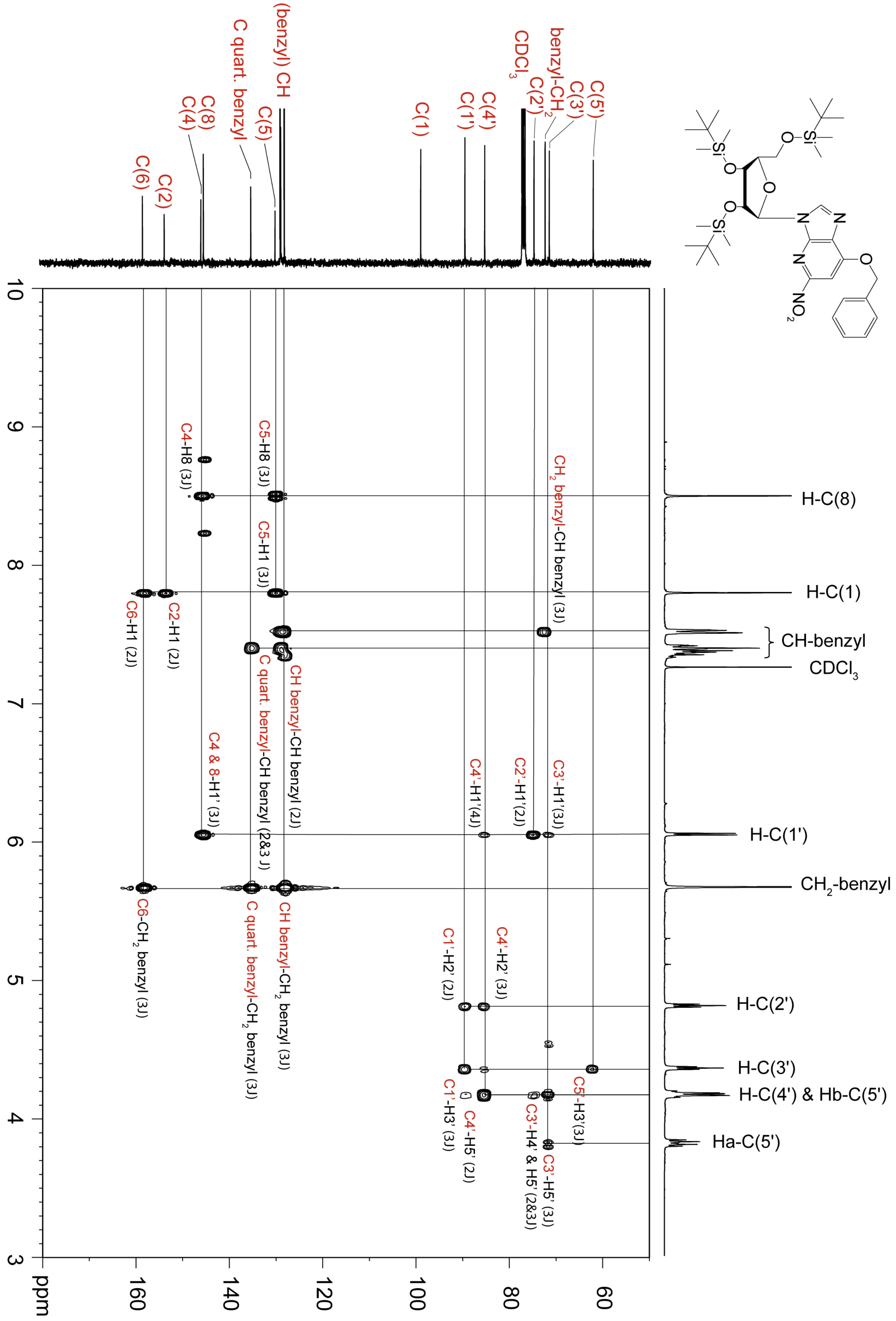

1-Deazaguanosine (6)

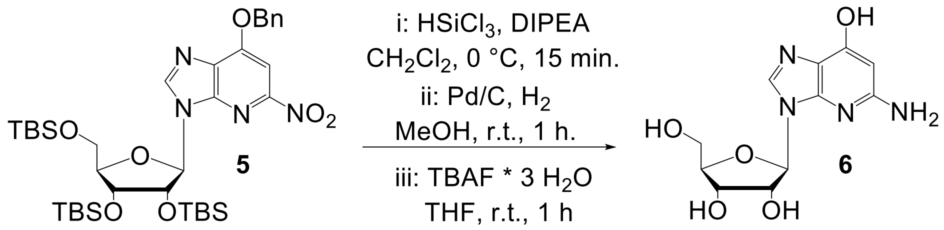

Step i: Compound **5** (443,00 mg, 594.51 µmol) was dissolved in 4.5 mL dry dichloromethane at 0 °C, then, *N*,*N*-diisopropylethylamine (DIPEA, 384.19 mg, 517.78 µL, 2.97 mmol) was added and the clear solution turned into brown. Subsequently, a solution made of 1.5 mL dichloromethane and trichlorosilane ($HSiCl_3$, 281.83 mg, 210.32 µL, 2.08 mmol) at 0 °C was added dropwise over a period of 10 minutes. After 30 minutes, the previously brown solution turned nearly colourless and reaction control showed complete consumption of the starting material. The reaction mixture was diluted with dichloromethane (10 mL) and was quenched by adding saturated sodium bicarbonate solution (10 mL). This mixture was allowed to stir for one hour. The resulting suspension was transferred into a separatory funnel. The aqueous layer was extracted two times with dichloromethane. The combined organic layers were washed two times with saturated sodium bicarbonate solution and once with brine, dried over Na_2SO_4 and evaporated to dryness. The product of step i was a slightly yellow foam of sufficient purity for the next step. TLC: (cyclohexane / ethyl acetate, 6:4): R_f = 0.62. **Step ii**: The product of step i and palladium on carbon 5% (Pd/C, 70.38 mg) were suspended in methanol (20 mL). A rubber septum was applied and hydrogen gas (balloon with syringe) was bubbled through the solution for 10 minutes. The mixture was allowed to stir for further 60 minutes under hydrogen atmosphere at room temperature. The catalyst was filtered off, and the filtrate was evaporated to dryness to yield a brownish solid, which was of sufficient purity for the next step. TLC: (cyclohexane / ethyl acetate, 6:4): R_f = 0.30. **Step iii**: The brownish solid of step ii was dissolved in tetrahydrofuran (4.7 mL) and tetra-*n*-butylammonium fluoride trihydrate (TBAF*3H_2O, 750.00 mg, 2.38 mmol) was added and stirred at room temperature for one hour. After complete deprotection, the product precipitated. The precipitated solid was filtered off and washed with several amounts of ether and was dried under high vacuum. The crude product was purified with reversed phase chromatography using 0 to 20 % acetonitrile in water as gradient. The pure product was dried over night at 100 °C under high vacuum to yield the anhydrous product. Yield: 120 mg of compound **6** as a white solid (72%). TLC: (methanol / dichloromethane, 2:8): R_f = 0.29. ESI-MS (m/z): $[M+H]^+$ calcd.: 283.10; found: 283.10 ^{1}H-NMR: (400 MHz, DMSO-*d*$_6$, 25 °C): δ = 3.53 (1H, m, ***H(a)***-C(5')); 3.64 (1H, m, ***H(b)***-C(5')); 3.91 (1H, m, ***H***-C(4')); 4.10 (1H, q, J=2.59 Hz, ***H***-C(3')); 4.54 (1H, t, J=5.66 Hz, ***H***-C(2')); 5.09 (1H, b, ***H***O-C(5')); 5.33 (1H, b, ***H***O-C(3')), 5.52 (1H, b, ***H***O-C(2')), 5.61 (2H, s, N***H₂***), 5.79 (1H, d, J=6.40 Hz, ***H***-C(1')); 5.82 (1H, s, ***H***-C(1)); 7.97 (1H, s, ***H***-C(8)); 10.59 (1H, s, O***H***). ^{13}C-NMR: (100 MHz, DMSO-*d*$_6$, 25 °C): δ =61.85 (***C***(5')), 70.80 (***C***(3')), 73.03 (***C***(2')), 85.44 (***C***(4')), 87.15 (***C***(1')), 89.60 (***C***(1)), 119.06 (***C***(5)), 136.78 (***C***(8)), 147.17 (***C***(4)), 157.16 (***C***(2)), 158.06 (***C***(6)).

^{1}H-NMR (400 MHz, DMSO-d_6, 25 °C) of compound **6**

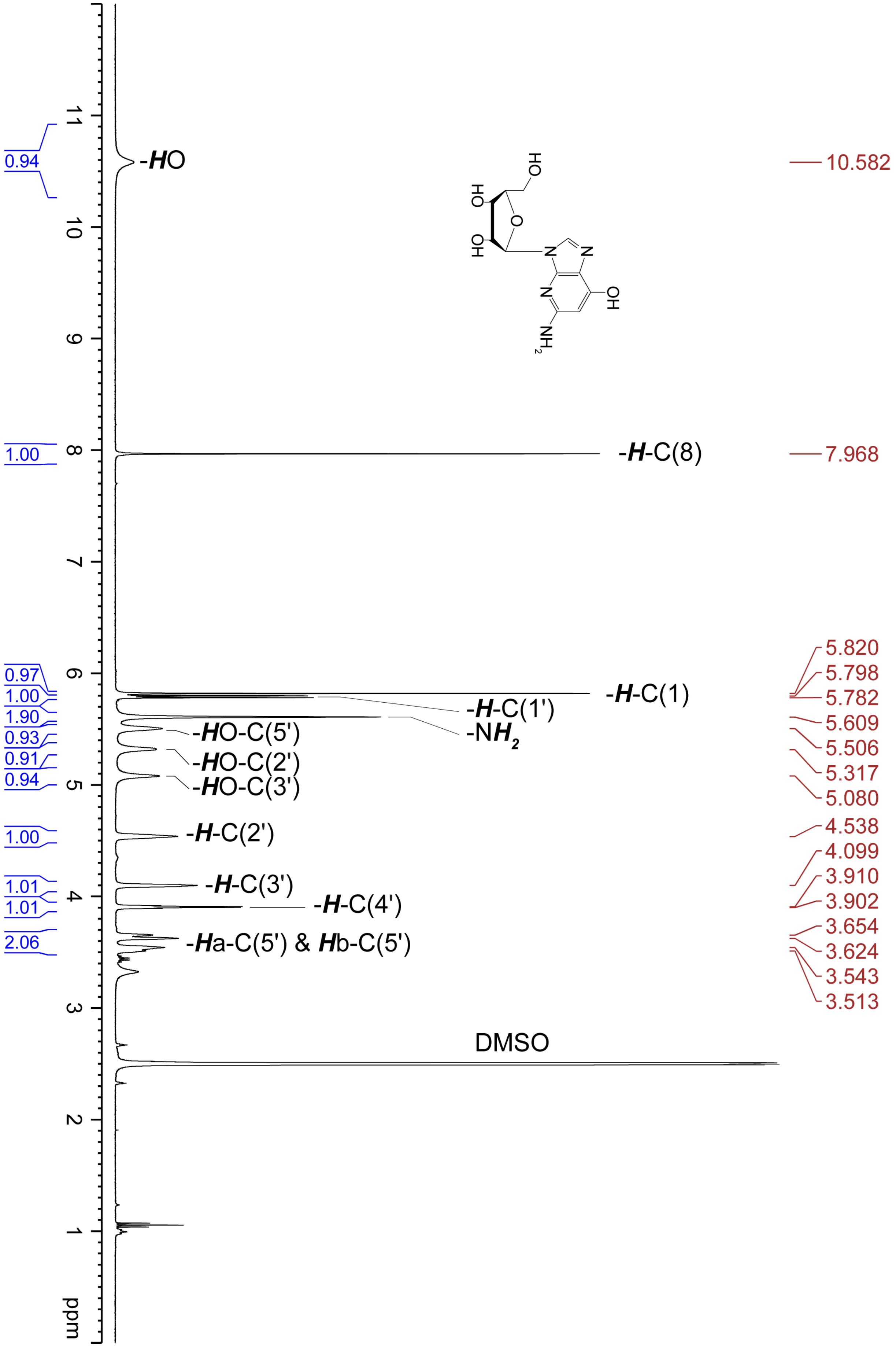

^{13}C-NMR (100 MHz, DMSO-d_6, 25 °C) of compound **6**

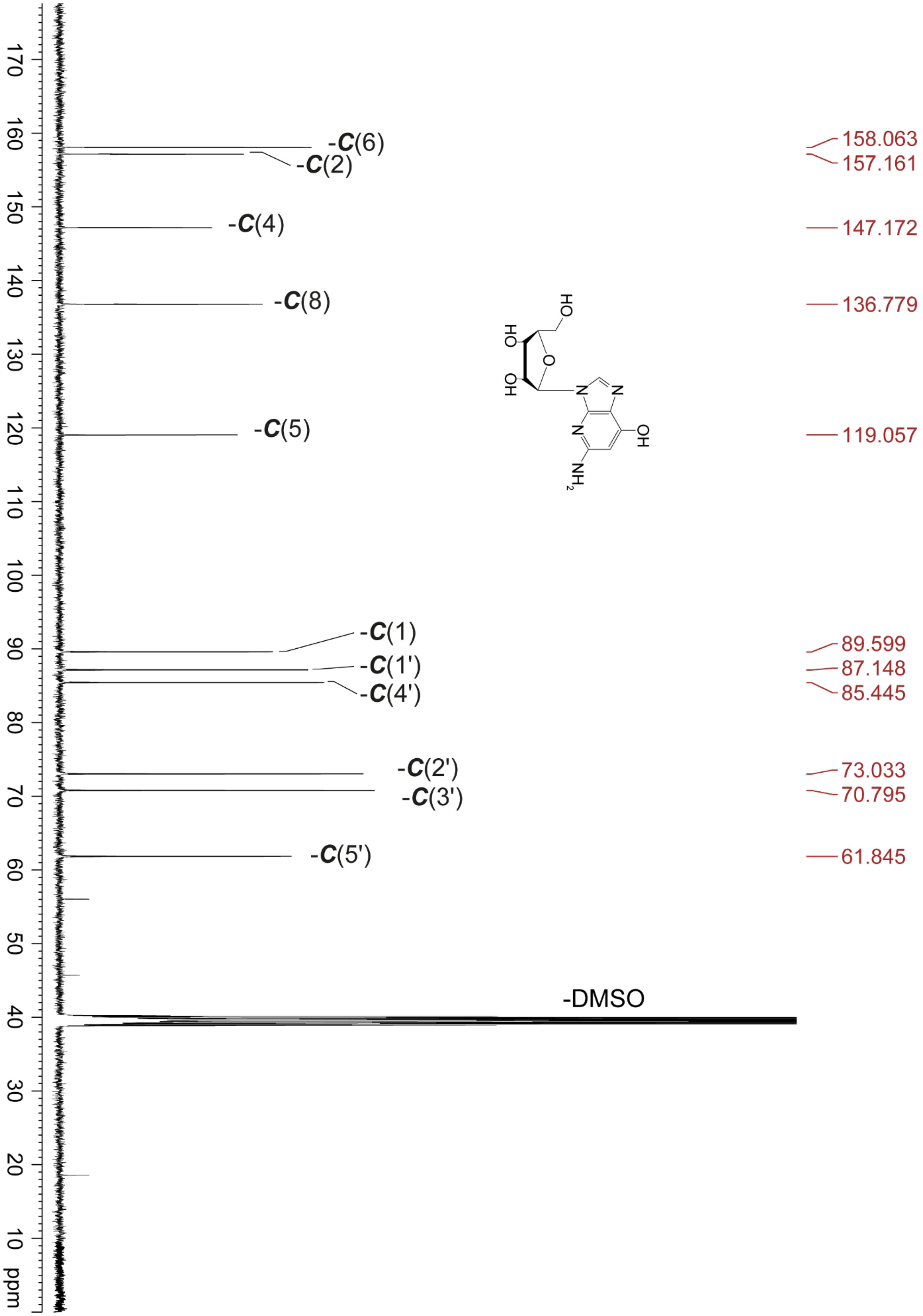

^{1}H, ^{13}C-HMBC NMR (400 MHz, DMSO-d_6, 25 °C) of compound **6**

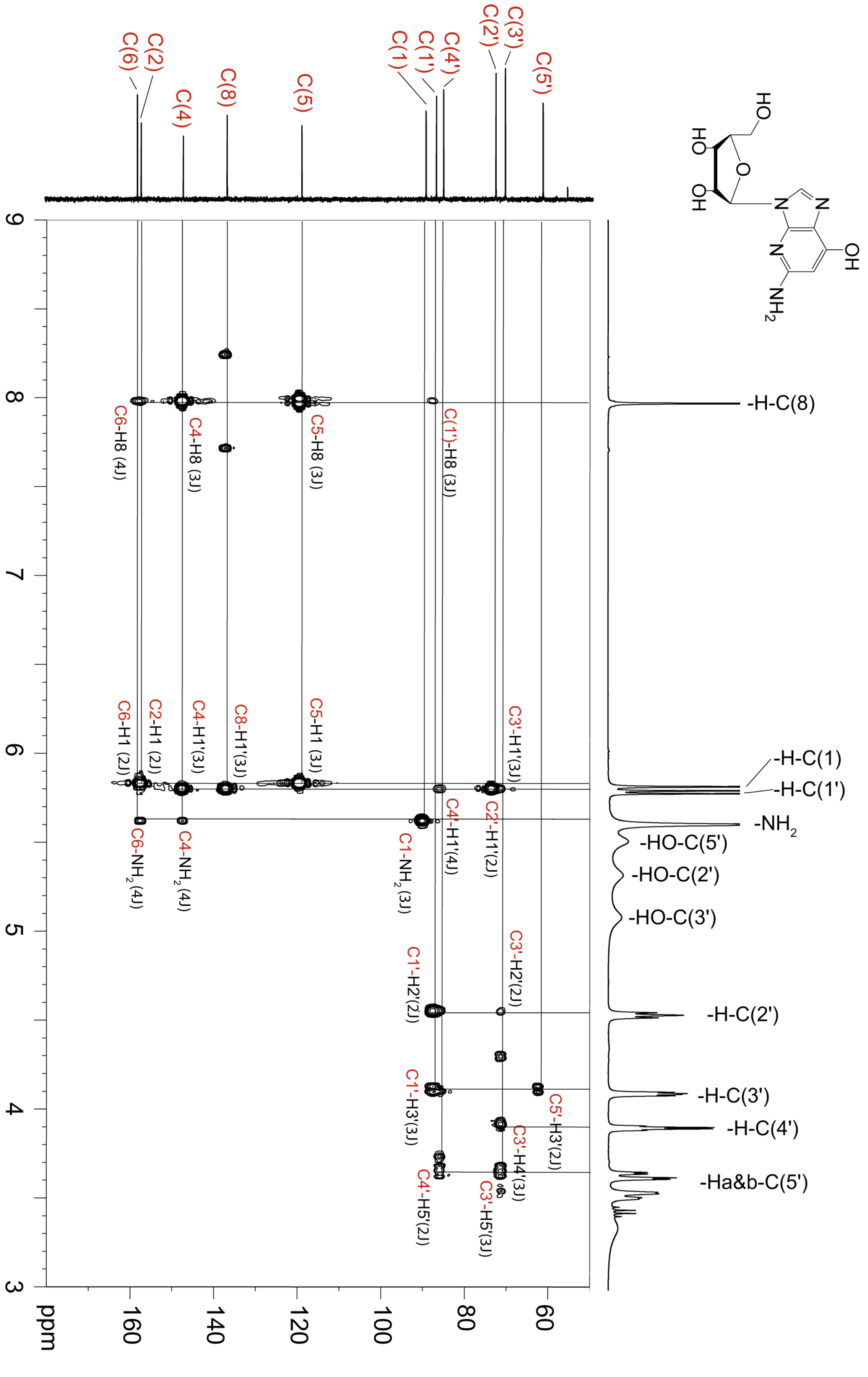

Synthesis of 1-deazaguanosine phosphoramidite (12)

2',3',5'-*O*-tris-(*tert*-Butyldimethylsilyl)- 1-deazaguanosine (7)

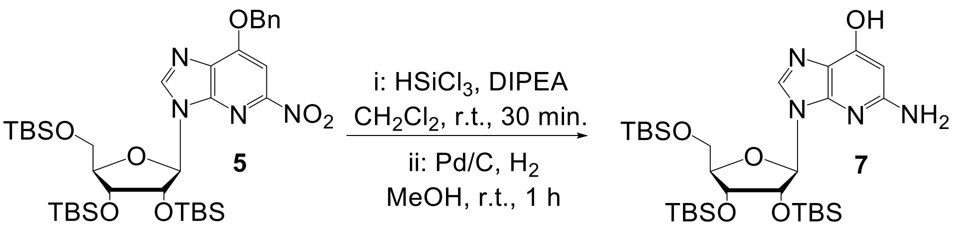

Step i: Compound **5** (580 mg, 778.36 µmol) was dissolved in dry dichloromethane (6 mL) at 0 °C, then, *N*,*N*-diisopropylethylamine (DIPEA, 503.01 mg, 678 µL, 3.89 mmol) was added and the clear solution turned into brown. Subsequently, a solution made of dry dichloromethane (2 mL) and trichlorosilane ($HSiCl_3$, 369 mg, 275 µL, 2.72 mmol) at 0 °C was added dropwise over a period of 10 minutes. After 30 minutes, the previously brown solution turned nearly colourless and reaction control showed complete consumption of the starting material. The reaction mixture was diluted with 10 mL dichloromethane and was quenched by adding saturated sodium bicarbonate solution (10 mL). This mixture was allowed to stir for one hour. The resulting suspension was transferred into a separatory funnel. The aqueous layer was extracted two times with dichloromethane. The combined organic layers were washed two times with saturated sodium bicarbonate solution and once with brine, dried over Na_2SO_4 and evaporated to dryness. The product of step i was a slightly yellow foam of sufficient purity for the next step. TLC: (cyclohexane / ethyl acetate, 6:4): R_f = 0.62. **Step ii**: The crude product of step i and palladium on carbon 5% (Pd/C, 92 mg) were suspended in methanol (20 mL). A rubber septum was applied and hydrogen gas (balloon with syringe) was bubbled through the solution for 10 minutes. The mixture was continued for stirring under hydrogen atmosphere for further 60 minutes at room temperature. The catalyst was filtered off, and the filtrate was evaporated to dryness to yield the crude product which was purified with silica gel chromatography using 15 to 25 % ethyl acetate in cyclohexane as gradient. Yield: 385 mg of compound **7** as a white solid (79 %). TLC: (cyclohexane / ethyl acetate, 6:4): R_f = 0.30. ESI-MS (m/z): $[M+H]^+$ calcd.: 625.36; found: 625.36. ^{1}H-NMR: (400 MHz, DMSO-*d*$_6$, 25 °C): δ = -0.3 (3H, s, Si-C***H*$_3$**); -0.11 (3H, s, Si-C***H*$_3$**); 0.09-0.13 (12H, m, Si-C***H*$_3$**); 0.72 (9H, s, Si-C(C***H*$_3$**)$_3$)); 0.92 (18H, d, J=9.05 Hz, Si-C(C***H*$_3$**)$_3$)); 3.71 (1H, m, ***H(a)***-C(5')); 3.87 - 3.96 (2H, m, ***H(b)***-C(5') & ***H***-C(4')); 4.22 (1H, t, J=6.12 Hz, ***H***-C(3')); 4.75 (1H, t, J=11.28 Hz, ***H***-C(2')), 5.6 (2H, s, -N***H*$_2$**); 5.79 (1H, ***H***-C(1)); 5.87 (1H, d, J=6.76 Hz, ***H***-C(1')); 7.97 (1H, s, ***H***-C(8)); 10.5 (1H, b, -O***H***). ^{13}C-NMR: (100 MHz, DMSO-*d*$_6$, 25 °C): δ = (-4.83) – (-4.63) (-***C***H$_3$-Si-***C***H$_3$); 17.56 - 18.02 (Si-C(***C***H$_3$)$_3$); 25.53 - 26.82 (Si-***C***(CH$_3$)$_3$); 62.87 (***C***(5')); 72.70 (***C***(3')); 74.48 (***C***(2')); 85.05 (***C***(4')); 85.56 (***C***(1')); 89.66 (***C***(1)); 118.61 (***C***(4)); 135.81 (***C***(8)); 156.78 (***C***(2)); 158.26 (***C***(6)).

^{1}H-NMR (400 MHz, DMSO-d_6, 25 °C) of compound **7**

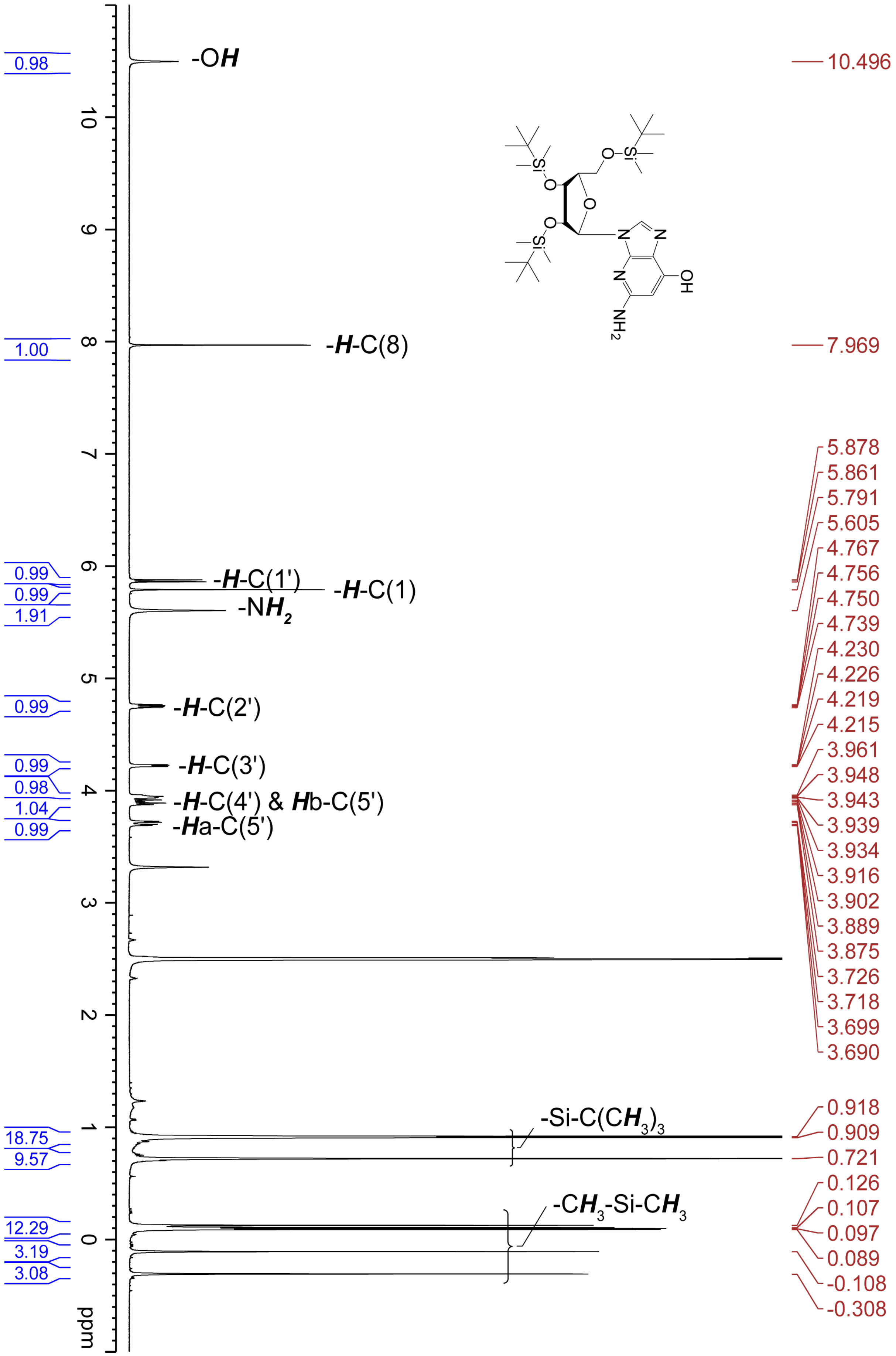

^{13}C-NMR (100 MHz, DMSO-d_6, 25 °C) of compound **7**

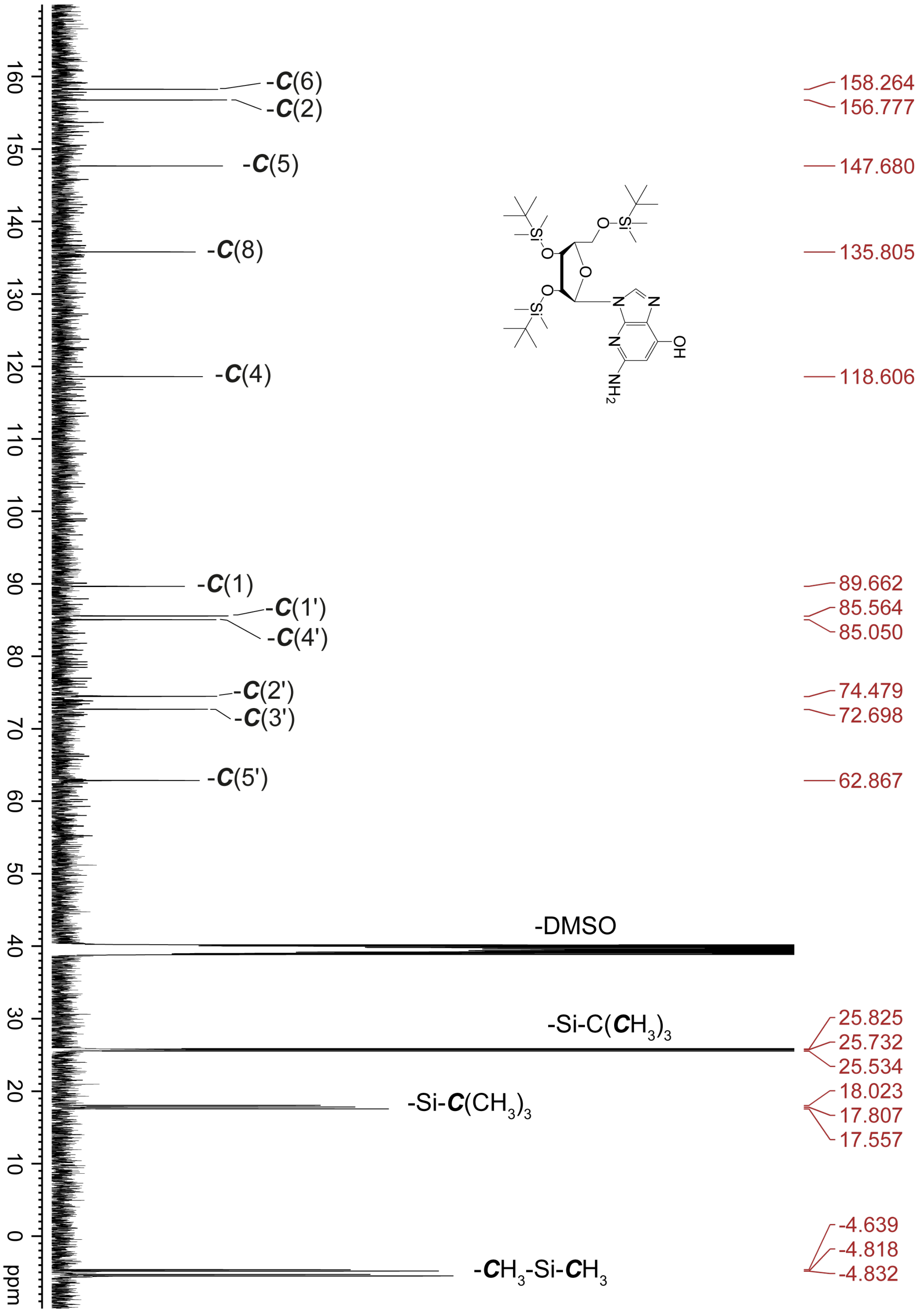

O^6-(*p*-Nitrophenylethyl)-N^2-trifluoroacetyl-2',3',5'-tris-*O*-(*tert*-butyldimethylsilyl)-1-deazaguanosine

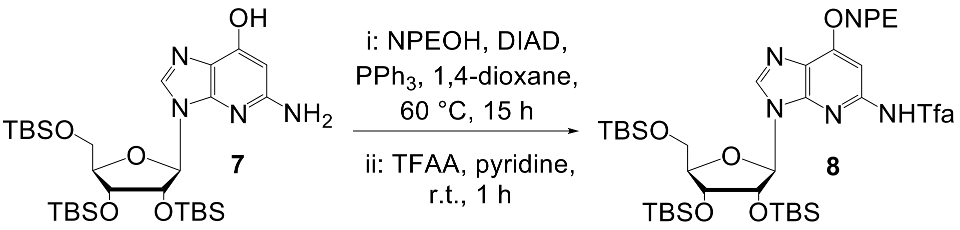

Step i: Compound **7** (2.40 g, 3.84 mmol), triphenylphosphine (PPh_3, 3.52 g, 13.44 mmol) and 2-(4-nitrophenyl)ethanol (NPEOH, 2.25 g, 13.44 mmol) were dissolved in 21 mL dry 1,4-dioxane and stirred for 10 minutes. Then, diisopropyl azodicarboxylate (DIAD, 2.72 g, 2.64 mL, 13.44 mmol) was added over a syringe and the mixture was stirred over night for 15 hours at 60 °C. Subsequently, the solvent and al volatiles were removed under reduced pressure, and the oily residue was purified over a short column using 20 to 30 % ethyl acetate in cyclohexane as gradient to get rid of most of the components. TLC: (1 % methanol in dichloromethane): R_f = 0.33. ESI-MS (m/z): $[M+H]^+$ calcd.: 774.41; found: 774.41. **Step ii:** The resulting product was dissolved in 24 mL dry pyridine and cooled to 0 °C, then trifluoroaceticanhydride (TFAA, 1.61 g, 1.07 mL, 7.67 mmol) was added over a syringe in 10 minutes at 0 °C. The reaction mixture was stirred for 90 minutes at room temperature. Afterwards the solvent was evaporated and the oily residue was dissolved in ethyl acetate and washed successively with 5% citric acid, saturated sodium bicarbonate solution and brine. The organic layer was dried over Na_2SO_4, filtrered and evaporated to dryness. The crude product was purified with silica gel chromatography using 0 to 15 % ethyl acetate in cyclohexane as gradient. Yield: 2.77 g of compound **8** as slightly yellow foam (83%). TLC: (cyclohexane / ethylacetate, 8:2): R_f = 0.54. ESI-MS (m/z): $[M+H]^+$ calcd.: 870.39; found: 870.39. ^{1}H-NMR: (400 MHz, $CDCl_3$, 25 °C): δ = -0.25 (3H, s, Si-C***H₃***); -0.03 (3H, s, Si-C***H₃***); 0.10 - 0.14 (12H, m, Si-C***H₃***); 0.80 (9H, s, Si-C(C***H₃***)₃)); 0.95 (18H, d, J=6.86 Hz, Si-C(C***H₃***)₃)); 3.34 (2H, t, J=6.79 Hz, -OCH₂C***H₂*** (NPE)); 3.88 (1H, m, ***H(a)***-C(5')); 3.97 (1H, m, ***H(b)***-C(5')); 4.13 (1H, m, ***H***-C(4')); 4.29 (1H, t, J=4.03 Hz, ***H***-C(3')); 4.47 (1H, t, J=4.72 Hz, ***H***-C(2')), 4.71 (2H, t, J=6.76 Hz, -OC***H₂***CH₂ (NPE)); 6.03 (1H, d, J=5.08 Hz, ***H***-C(1')); 7.52 (2H, d, J=8.75 Hz, o-***H***-phenyl (NPE)); 7.72 (1H, s, ***H***-C(1)); 8.18 (2H, d, J=8.73 Hz, m-***H***-phenyl (NPE)); 8.29 (1H, s, ***H***-C(8)); 8.37 (1H, b, -N***H***). ^{13}C-NMR: (100 MHz, $CDCl_3$, 25 °C): δ = (-5.26) – (-4.22) (-***C***H₃-Si-***C***H₃); 18.02 – 18.68 (Si-C(***C***H₃)₃); 25.75 - 26.23 (Si-***C***(CH₃)₃); 35.52 (OCH₂***C***H₂ (NPE)); 62.67 (***C***(5')); 69.72 (O-***C***H₂CH₂ (NPE)); 72.07 (***C***(3')); 76.61 (***C***(2')); 85.50 (***C***(4')); 87.98 (***C***(1')); 94.46 (***C***(1)); 115.69 (q, J= 288.51 Hz, ***C***F₃); 123.95 (m-***C***H-phenyl (NPE)); 124.29 (***C***(5)); 130.17 (m-***C***H-phenyl (NPE));140.67 (***C***(8)); 145.50 (CH₂-***C***(phenyl)); 146.15 (***C***(2)); 146.65 (***C***(4)); 147.11 (p-***C***H-phenyl (NPE)); 154.82 (q, J=38.02 Hz, ***C***=O(Tfa)); 158.20 (***C***(6)).

[1] H-NMR (400 MHz, $CDCl_3$, 25 °C) of compound **8**

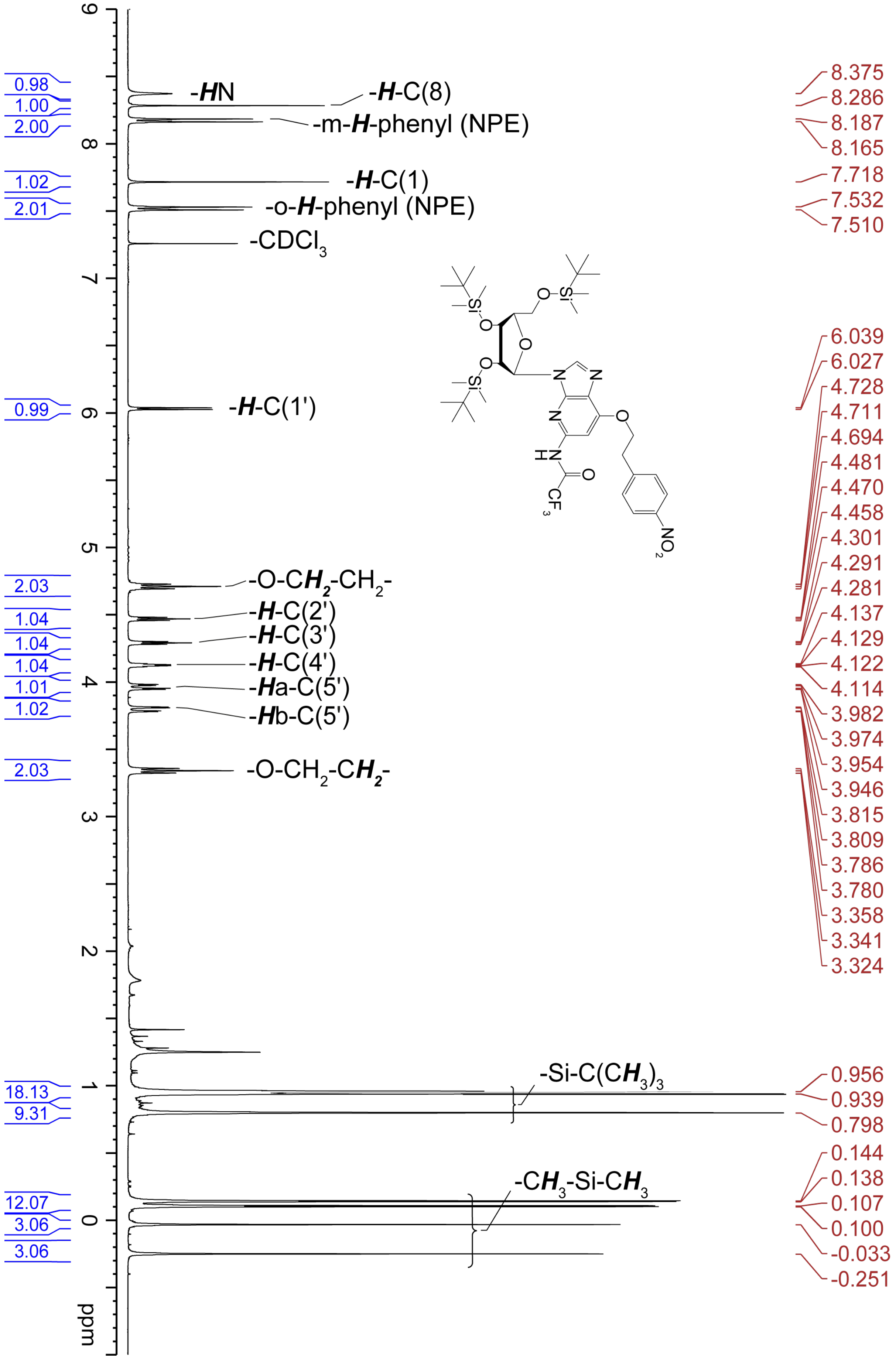

^{13}C-NMR (100 MHz, $CDCl_3$, 25 °C) of compound **8**

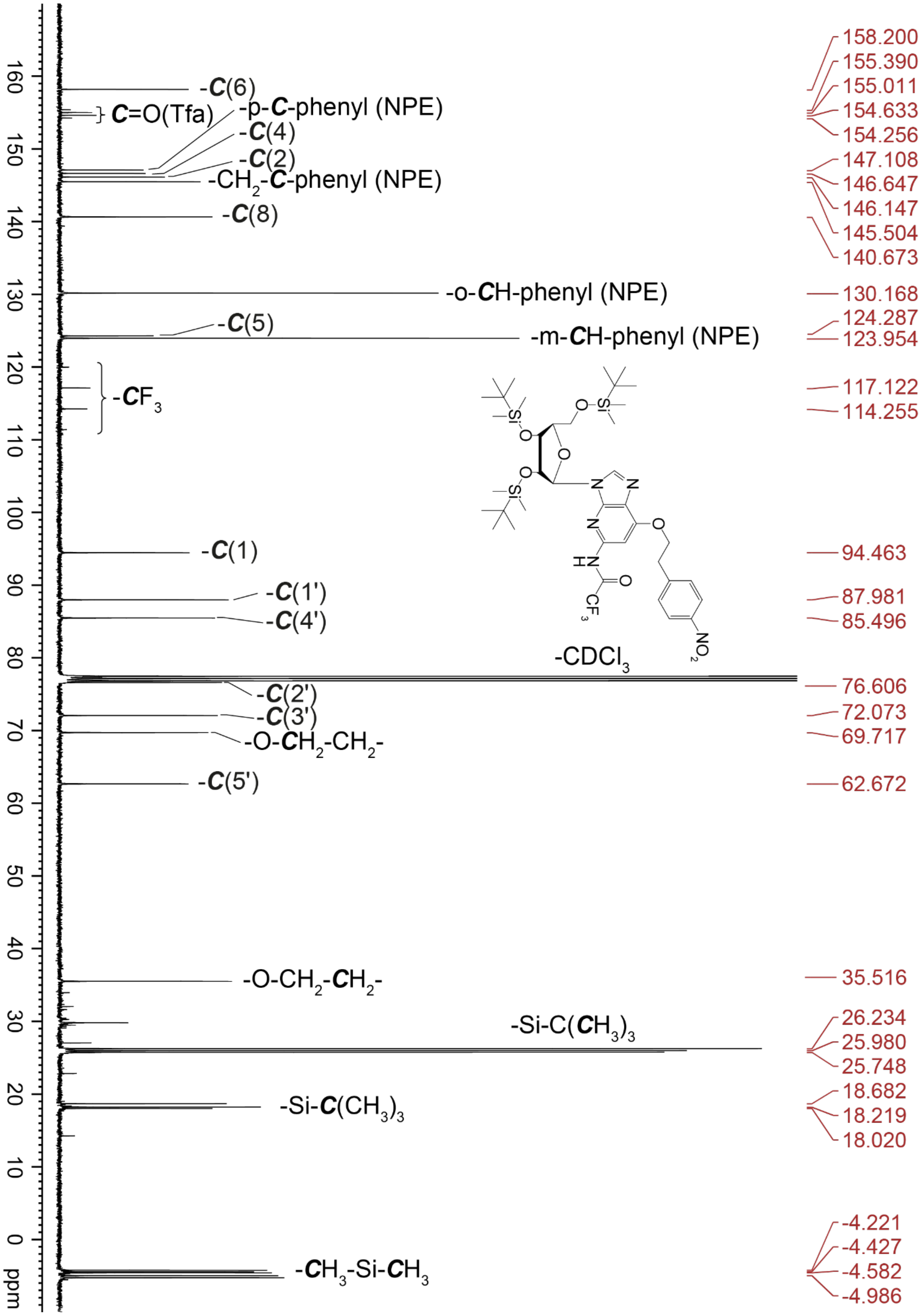

O^6-(*p*-Nitrophenylethyl)-N^2-trifluoroacetyl-1-deazaguanosine (9)

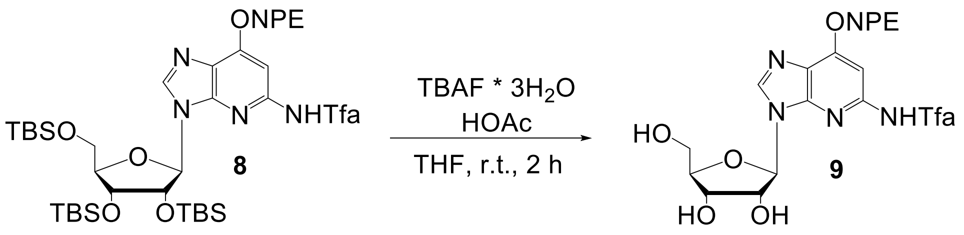

Compound **8** (1.54 g, 1.77 mmol), tetra-*n*-butylammoniumfluoride trihydrate (TBAF $3H_2O$, 2.23 g, 7.08 mmol) and a few drops of acetic acid were dissolved in tetrahydrofuran (15 mL) and stirred at room temperature for two hours. Afterwards, the solvent was removed and the crude product was purified on silica gel chromatography using 0 to 10 % methanol in dichloromethane as gradient. Yield: 920 mg of compound **9** as a white solid (98%). TLC: (methanol / dichloromathane 1:9): R_f = 0.52. ESI-MS (m/z): $[M+H]^+$ calcd.: 528.13; found: 528.13. ^{1}H-NMR: (400 MHz, DMSO-d_6, 25 °C): δ = 3.32 (2H, t, J=13.04 Hz, -OCH$_2$C***H₂*** (NPE)); 3.54 (1H, m, ***H(a)***-C(5')); 3.65 (1H, m, ***H(b)***-C(5')); 3.91 (1H, m, ***H***-C(4')); 4.16 (1H, q, J=4.23 Hz, ***H***-C(3')); 4.56 (1H, q J=6.60 Hz, ***H***-C(2')), 4.77 (2H, t, J=6.60 Hz, -OC***H₂***CH$_2$ (NPE)); 5.01 (1H, t, J=5.44 Hz, ***H***O-C(5')); 5.18 (1H, d, J=4.64 Hz, ***H***O-C(3')); 5.43 (1H, d, J=6.00 Hz, ***H***O-C(2')); 6.03 (1H, d, J=6.04 Hz, ***H***-C(1')); 7.45 (1H, s, ***H***-C(1)); 7.67 (2H, d, J=8.80 Hz, o-***H***-phenyl (NPE)); 8.19 (2H, d, J=8.80 Hz, m-***H***-phenyl (NPE)); 8.56 (1H, s, ***H***-C(8)); 11.95 (1H, b, -N***H***). ^{13}C-NMR: (100 MHz, DMSO-d_6, 25 °C): δ = 35.05 (OCH$_2$***C***H$_2$ (NPE)); 61.75 (***C***(5')); 69.89 (O-***C***H$_2$CH$_2$ (NPE)); 70.79 (***C***(3')); 74.03 (***C***(2')); 85.75 (***C***(4')); 86.85 (***C***(1')); 96.93 (***C***(1)); 115.71 (q, J= 288.42 Hz, ***C***F$_3$); 123.48 (***C***(5)); 123.91 (m-***C***H-phenyl (NPE)); 130.82 (m-***C***H-phenyl (NPE));141..86 (***C***(8)); 146.79 (CH$_2$-***C***(phenyl)); 146.91 (***C***(2)); 147.07 (***C***(4)); 147.49 (p-***C***H-phenyl (NPE)); 155.03 (q, J= 37.55 Hz, ***C***=O(Tfa)); 157.64 (***C***(6)).

^{1}H-NMR (400 MHz, DMSO-d_6, 25 °C) of compound **9**

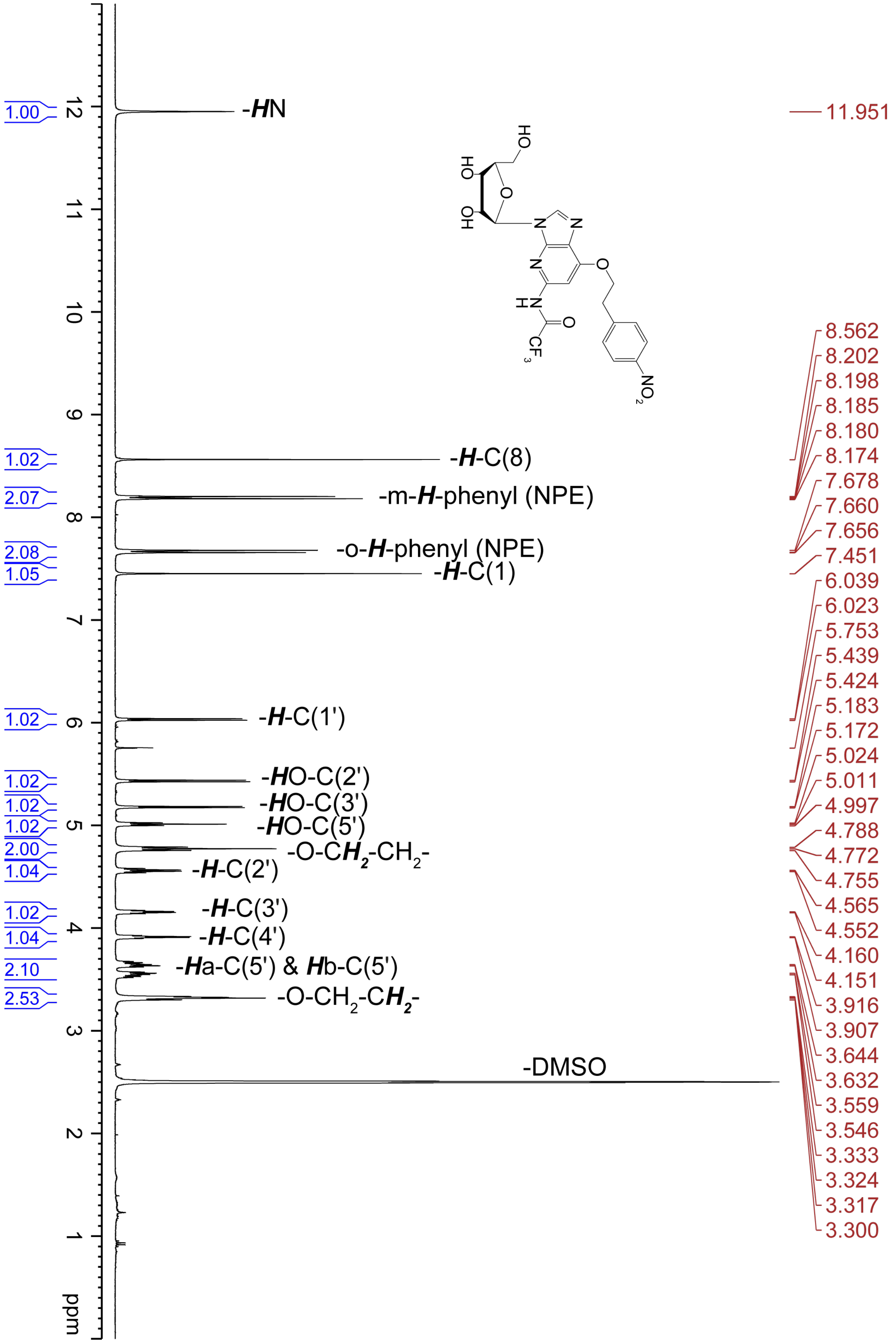

^{13}C-NMR (100 MHz, DMSO-d_6, 25 °C) of compound **9**

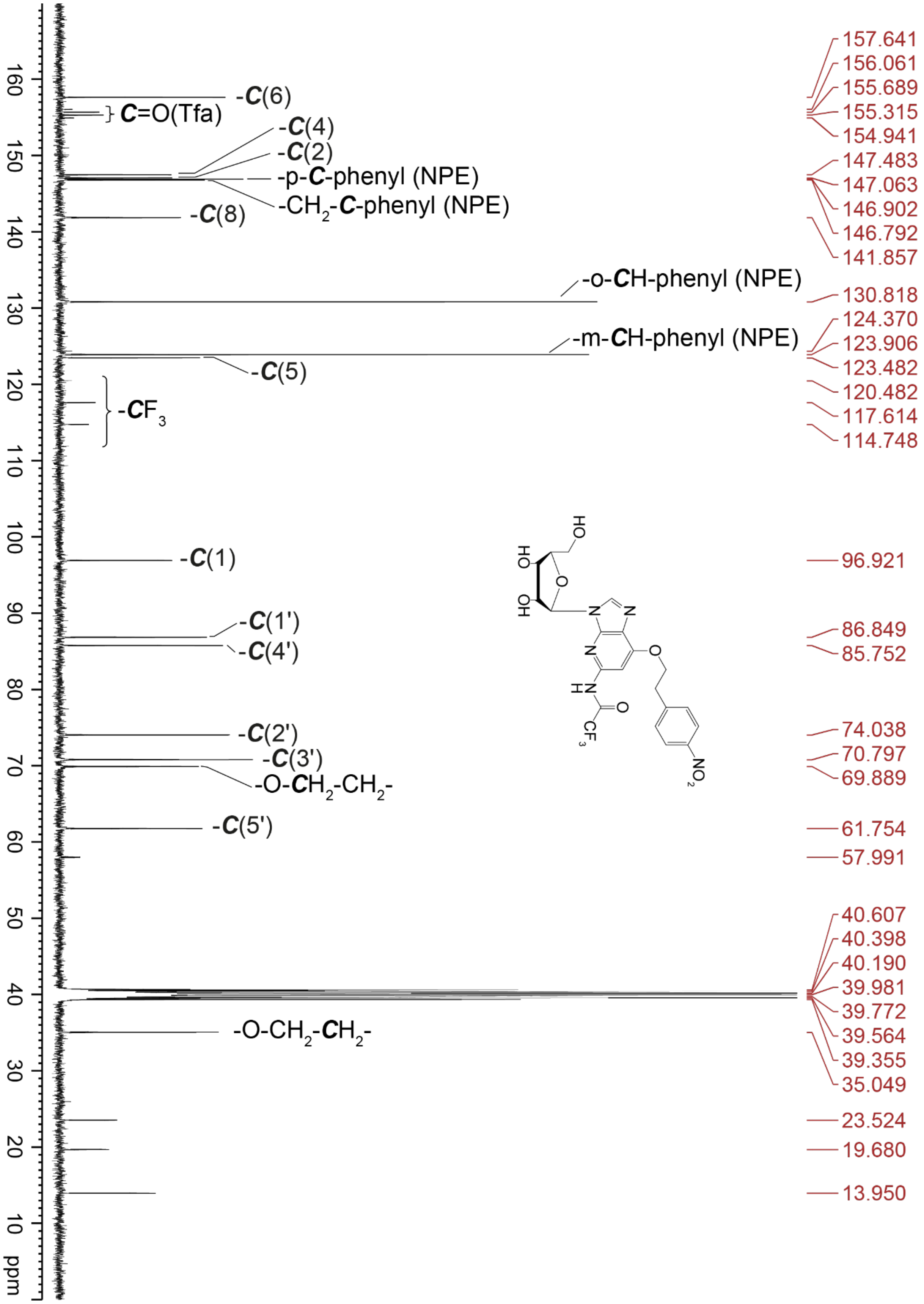

O^6-(*p*-Nitrophenylethyl)-2'-*O*-(tert-butyldimethylsilyl)-N^2-trifluoroacetyl-1-deazaguanosine (10)

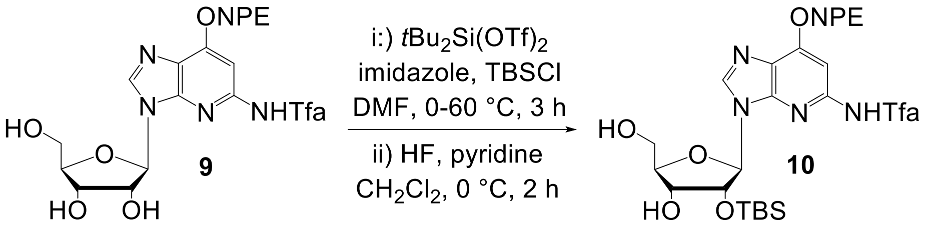

Step i: Compound **9** (790 mg, 1.50 mmol) was coevaporated twice with dry pyridine, then dissolved in 3 mL *N*,*N*-dimethylformamide and cooled to 0 °C in an ice bath. Then, di-*tert*-butylsilyl-bis(trifluoromethansulfonate) ($tBu_2Si(OTf)_2$, 725.70 mg, 537.56 mL, 1.65 mmol) was added dropwise and stirred for 30 minutes at the same temperature. Afterwards, imidazole (509.87 mg, 7.5 mmol) was added and stirring was continued for 30 minutes at room temperature. Subsequently, *tert*-butyldimethylsilylchloride (TBSCl, 270.91 mg, 1.8 mmol) was added and the reaction mixture was heated up to 60 °C and stirred for two hours. After quenching with methanol, the whole was concentrated under reduced pressure, dissolved in dichloromethane and transferred into a separatory funnel. The organic layer was washed once with saturated sodium bicarbonate solution and twice with brine, dried over Na_2SO_4 and evaporated to dryness. **Step ii:** The residue was dissolved in dry dichloromethane (7.5 mL) and cooled to 0 °C. Meanwhile, a solution made of pyridine (980 µL) and HF in pyridine (70%, 164.64 mg, 150 µL, 5.76 mmol) was prepared and added to the dissolved compound and stirred for 2 hours at 0 °C. Afterwards, the mixture was washed successively with water, twice with saturated sodium bicarbonate solution and brine. The resulting organic layer was dried over Na_2SO_4 and evaporated. The crude product was purified with silica gel chromatography using 0 to 3 % methanol in dichloromethane as gradient. Yield: 800 mg of compound **10** as a white foam (83%). TLC: (5 % methanol in dichloromethane): R_f = 0.44. ESI-MS (m/z): $[M+H]^+$ calcd.: 642.22; found: 642.22. ^{1}H-NMR: (400 MHz, $CDCl_3$, 25 °C): δ = -0.25 (3H, s, Si-C***H₃***); -0.09 (3H, s, Si-C***H₃***); 0.70 (9H, s, Si-C(C***H₃***)₃)); 3.30 (2H, m, -OCH₂C***H₂*** (NPE)); 3.56 (1H, m, ***H(a)***-C(5')); 3.68 (1H, m, ***H(b)***-C(5')); 4.12 (1H, m, ***H***-C(4')); 4.69 (1H, m, ***H***-C(3')); 4.69 (1H, m, ***H***-C(2')), 4.75 (2H, t, J=6.64 Hz, -OC***H₂***CH₂ (NPE)); 5.06 (2H, m, ***H***O-C(3 '& 5')); 6.06 (1H, d, J=6.48 Hz, ***H***-C(1')); 7.40 (1H, s, ***H***-C(1)); 7.66 (2H, d, J=8.72 Hz, o-***H***-phenyl (NPE)); 8.19 (2H, d, J=8.76 Hz, m-***H***-phenyl (NPE)); 8.57 (1H, s, ***H***-C(8)); 11.85 (1H, b, -N***H***). ^{13}C-NMR: (100 MHz, $CDCl_3$, 25 °C): δ = -5.42 & -4.96 (-***C***H₃-Si-***C***H₃); 17.72 (Si-C(***C***H₃)₃); 25.49 (Si-***C***(CH₃)₃); 34.56 (OCH₂***C***H₂ (NPE)); 61.29 (***C***(5')); 69.35 (O-***C***H₂CH₂ (NPE)); 70.52 (***C***(3')); 75.60 (***C***(2')); 85.90 (***C***(4')); 86.29 (***C***(1')); 96.55 (***C***(1)); 115.70 (q, J= 288.59 Hz, ***C***F₃); 123.02 (***C***(5)); 123.42 (m-***C***H-phenyl (NPE)); 123.89; 130.36 (m-***C***H-phenyl (NPE));141.28 (***C***(8)); 146.32; 146.37; 146.54; 146.90; 149.60 (***C***(4)); 154.97 (q, J=37.69 Hz, ***C***=O(Tfa)); 157.17 (***C***(6)).

^{1}H-NMR (400 MHz, DMSO-d_6, 25 °C) of compound **10**

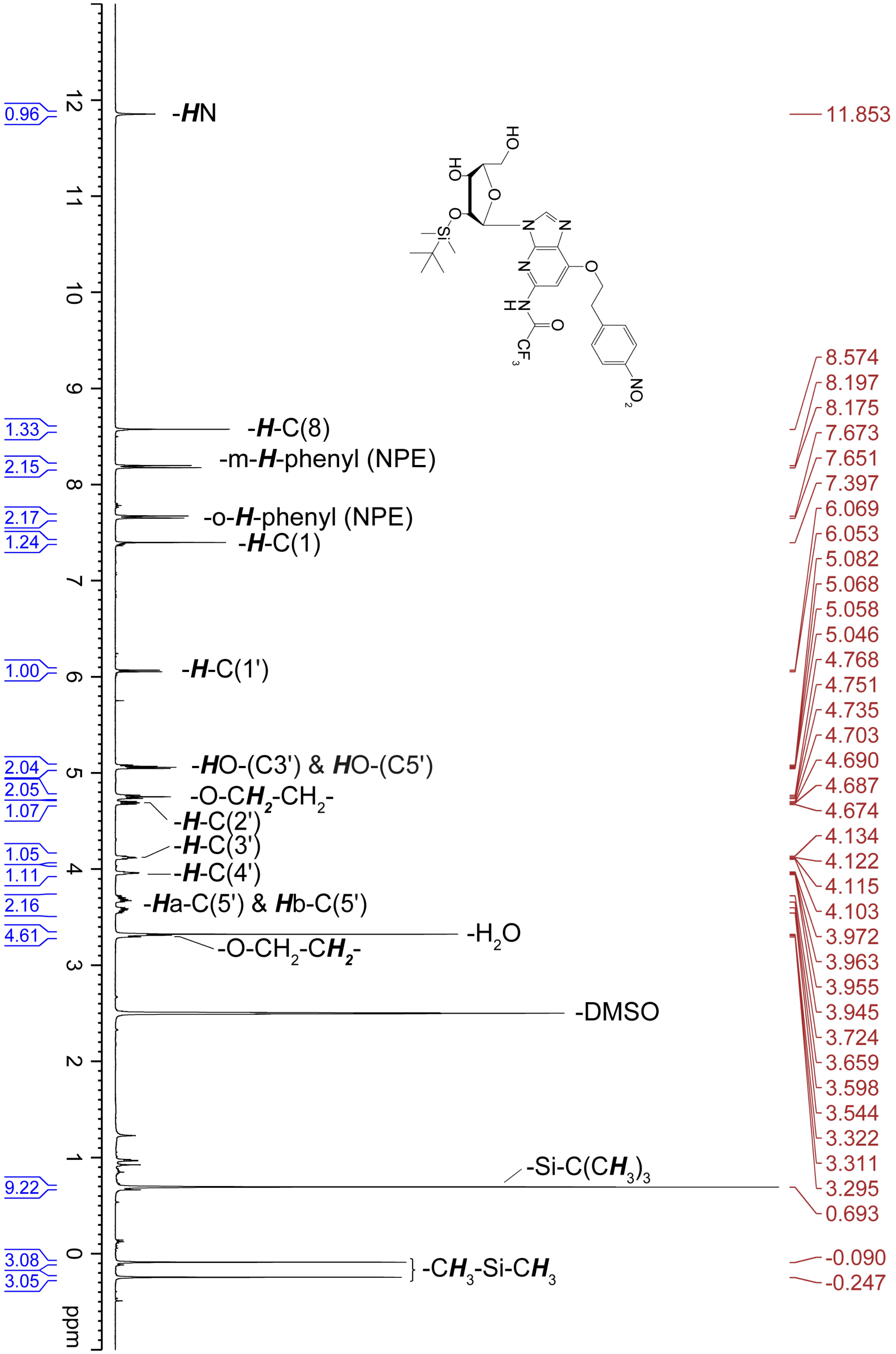

^{13}C-NMR (100 MHz, DMSO-d_6, 25 °C) of compound **10**

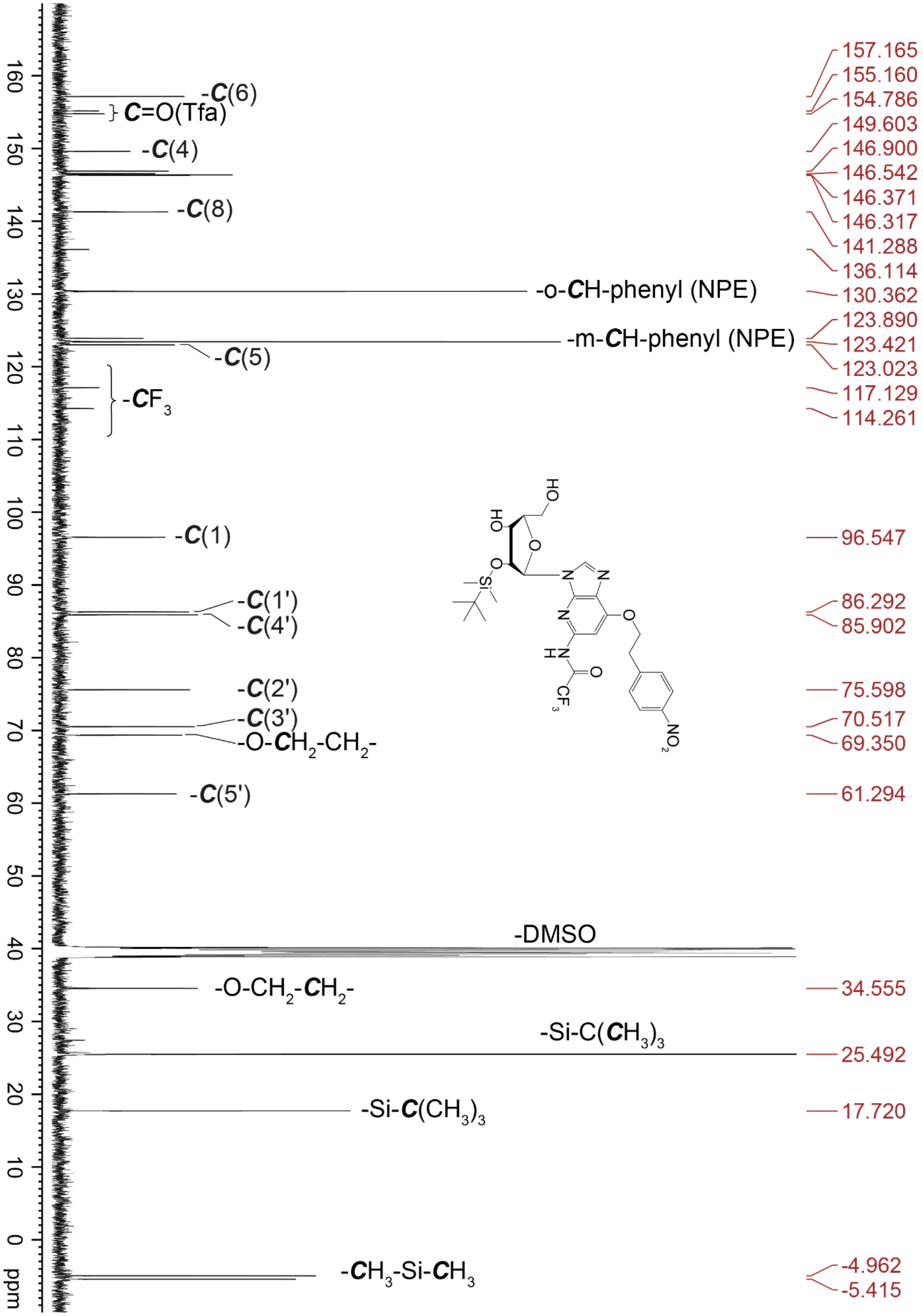

5'-*O*-(4,4'-Dimethoxytrityl)-O^6-(*p*-nitrophenylethyl)-2'-*O*-(tert-butyldimethylsilyl)-N^2-trifluoroacetyl-1-deazaguanosine (11)

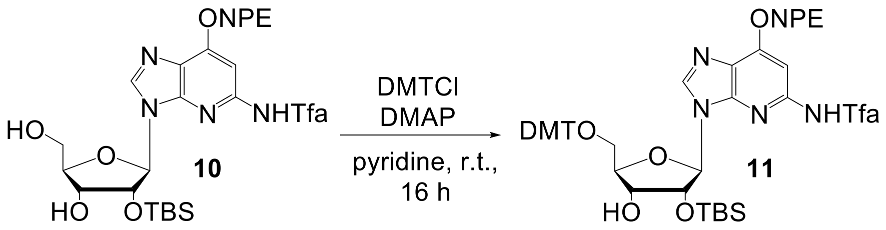

Compound **10** (800 mg, 1.25 mmol) and 4-dimethylaminopyridine (DMAP, 30.46 mg, 0.25 mmol) were coevaporated three times with dry pyridine and finally dissolved in dry pyridine (13.5 mL). Then, 4,4'-dimethoxytrityl chloride (DMTCl, 549.16 mg, 1.62 mmol) was added in two portions over an hour. By each addition of 4,4'-dimethoxytrityl chloride, the reaction solution was heated for one minute to 35 °C. After complete addition of 4,4'-dimethoxytrityl chloride, the mixture was stirred for 16 hours overnight at room temperature. The reaction was quenched by the addition of methanol (1 mL) and was evaporated to dryness. The remaining oily residue was dissolved in dichloromethane and washed successively with 5 % citric acid solution, saturated sodium bicarbonate solution and brine. The organic layer was dried over Na_2SO_4, filtered and concentrated under vacuum. The crude product was purified on silica gel chromatography using 0 to 30 % ethyl acetate in dichloromethane as gradient. Yield: 1.03 g of compound **11** as a slightly yellow foam (88%). TLC: (cyclohexane / ethyl acetate, 1:1): R_f = 0.71. ESI-MS (m/z): $[M+H]^+$ calcd.: 944.35; found: 944.35. ^{1}H-NMR: (400 MHz, $CDCl_3$, 25 °C): δ = -0.18 (3H, s, Si-C***H₃***); -0.04 (3H, s, Si-C***H₃***); 0.73 (9H, s, Si-C(C***H₃***)₃)); 3.22 (1H, m, ***H(b)***-C(5')); 3.32 (3H, m, -OCH₂C***H₂*** (NPE) & ***H(a)***-C(5')); 3.71 (6H, s, 2xO-C***H₃***); 4.07 (1H, m, ***H***-C(4')); 4.18 (1H, m, ***H***-C(3')); 4.75 (3H, m, ***H***-C(2') & -OC***H₂***CH₂ (NPE)); 5.08 (2H, m, ***H***O-C(3')); 6.09 (1H, d, J=5.60 Hz, ***H***-C(1')); 6.79 -7.36 (13H, m, C***H***-arom- DMT); 7.44 (1H, s, ***H***-C(1)); 7.66 (2H, d, J=8.74 Hz, o-***H***-phenyl (NPE)); 8.18 (2H, d, J=8.75 Hz, m-***H***-phenyl (NPE)); 8.43 (1H, s, ***H***-C(8)); 11.79 (1H, b, -N***H***). ^{13}C-NMR: (100 MHz, DMSO-d_6, 25 °C): δ = = -5.33 & -4.86 (-***C***H₃-Si-***C***H₃); 17.76 (Si-C(***C***H₃)₃); 25.50 (Si-***C***(CH₃)₃); 34.54 (OCH₂***C***H₂ (NPE)); 54.96 (2xO-***C***H₃); 63.94 (***C***(5')); 69.35 (O-***C***H₂CH₂ (NPE)); 70.47 (***C***(3')); 75.29 (***C***(2')); 83.90 (***C***(4')); 85.55 (***C***. quart. DMT); 86.78 (***C***(1')); 96.31 (***C***(1)); 115.66 (q, J= 288.73 Hz, ***C***F₃); 123.01 (***C***(5)); 123.41 (m-***C***H-phenyl (NPE)); 130.35 (o-***C***H-phenyl (NPE)); 126.67 – 135.45 (***C***H-arom.-DMT); 140.91 (***C***(8)); 144.85; 146.31 (p-***C***H-phenyl (NPE));146.37 (CH₂-***C***(phenyl)); 146.78 (***C***(4)); 154.84 (q, J=37.37 Hz, ***C***=O(Tfa)); 155.02 (***C***(6)).

^{1}H-NMR (100 MHz, DMSO-d_6, 25 °C) of compound **11**

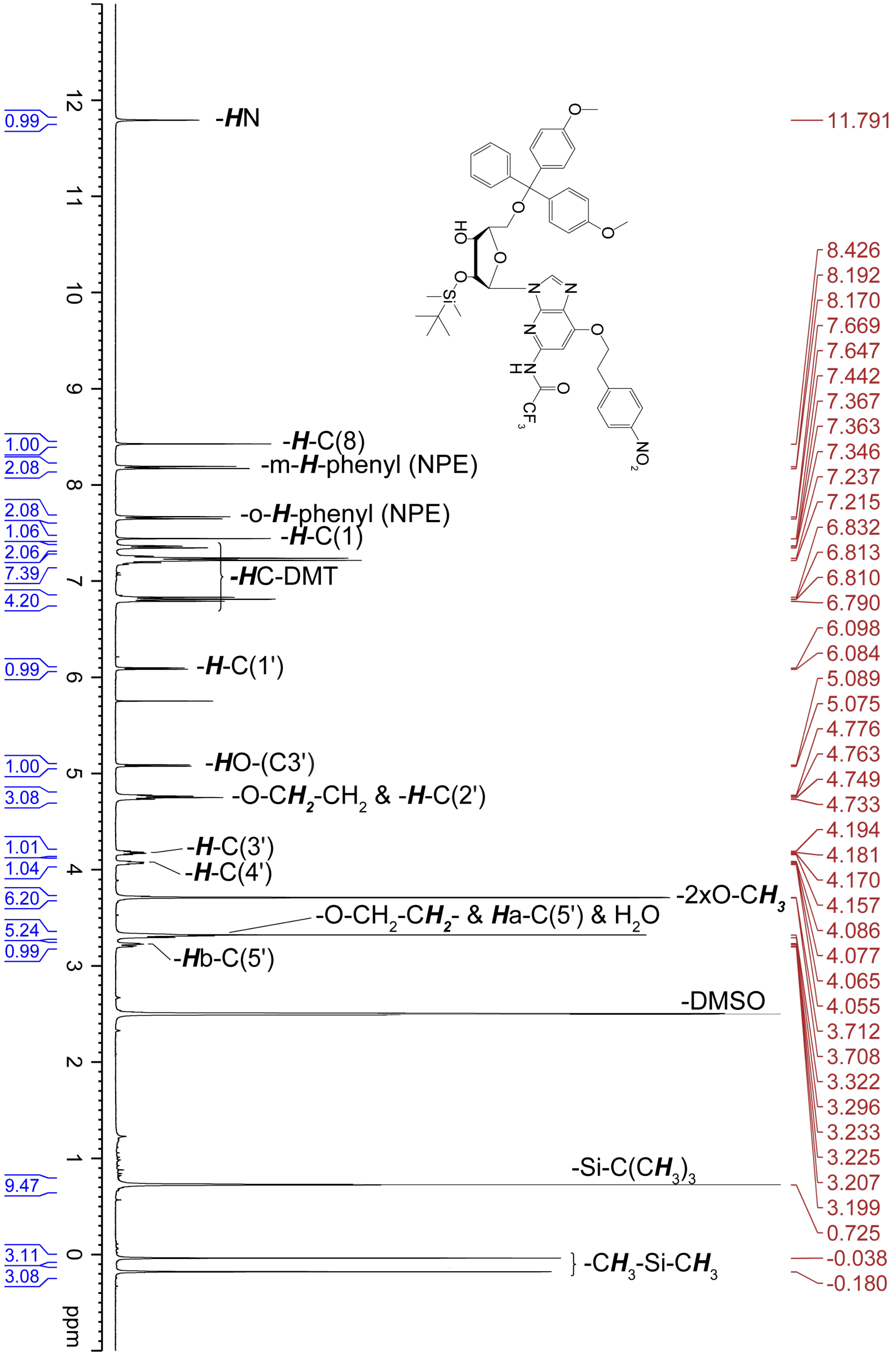

^{13}C-NMR (100 MHz, DMSO-d_6, 25 °C) of compound **11**

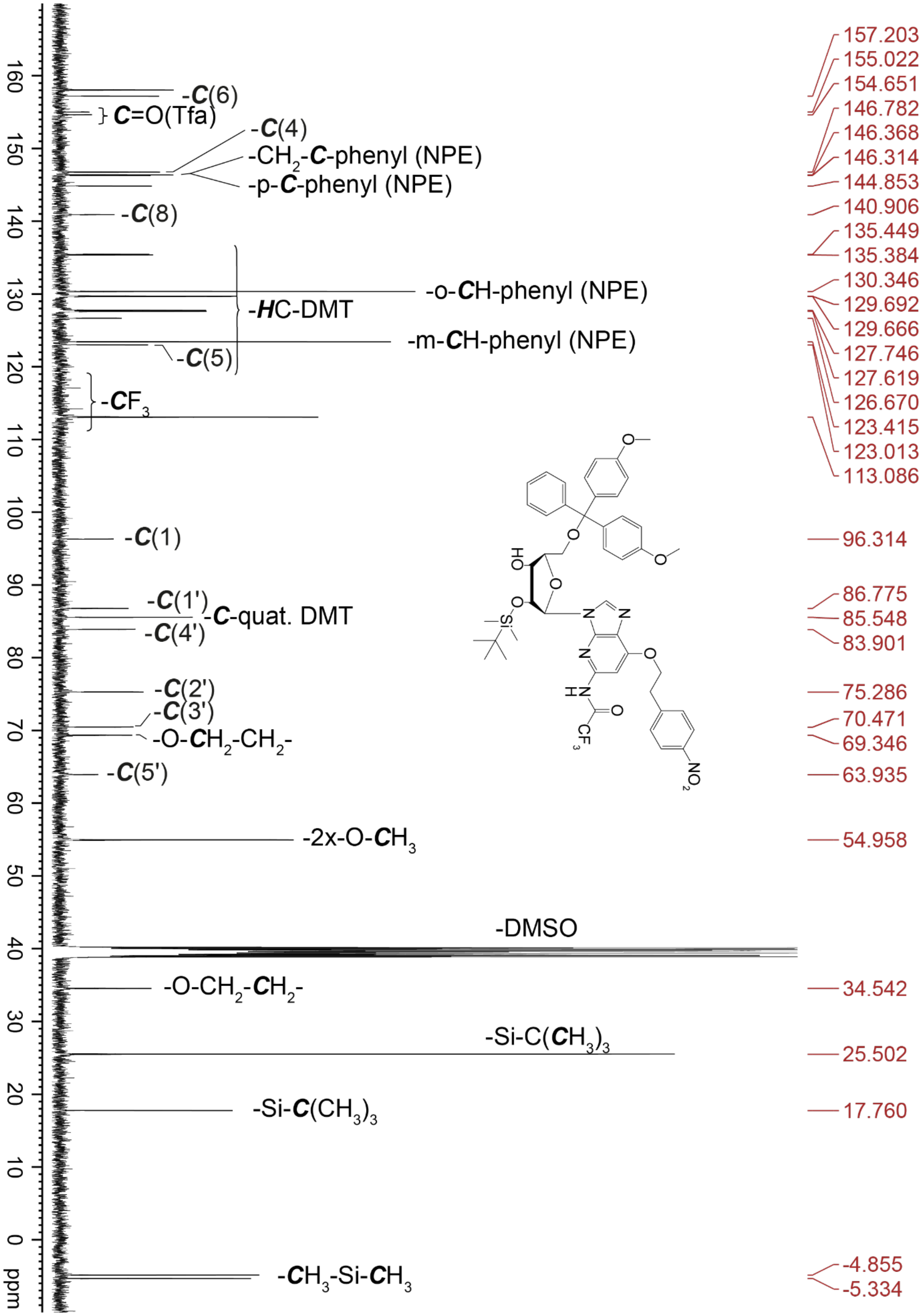

5'-*O*-(4,4'-Dimethoxytrityl)-*O*^{6}-(*p*-nitrophenylethyl)-2'-*O*-(tert-butyldimethylsilyl)-*N*^{2}-trifluoroacetyl-1-deazaguanosine 3'-O-2-cyanoethyl-N,N-diisopropylphosphoramidite (12)

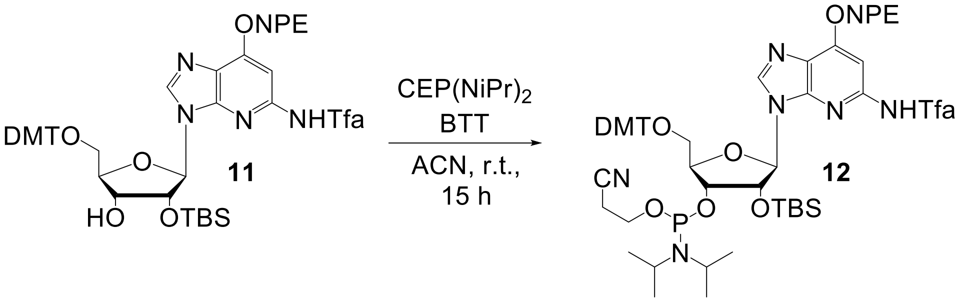

Compound **11** (527 mg, 558.23 µmol) and 5-(benzylthio)-1*H*-tetrazol (BTT, 53.66 mg, 279.15 µmol) were dissolved in dry acetonitrile (12 mL), then molecular sieve was added and stirred at room temperature for two hours. Afterwards, 2-cyanoethyl-*N*,*N*,*N'*,*N'*-tetraisopropyl-phosphorodiamidite (CEP(N*i*Pr)$_2$, 673.04 mg, 711.46 µl, 2.23 mmol) was added and the mixture was stirred at room temperature over night for 15 hours under argon atmosphere. The solvent was evaporated and the crude product was purified using 10 to 30 % ethyl acetate in cyclohexane as gradient. Yield: 448 mg of compound **12** as a white foam (70 %). TLC: (cyclohexane / ethyl acetate, 6:4): R_f = 0.62. ESI-MS (m/z): $[M+H]^+$ calcd.: 1144.46; found: 1144.46. ^{1}H-NMR: (400 MHz, $CDCl_3$, 25 °C): δ = -0.25 (3H, s, Si-C***H₃***); -0.03 (3H, d, J=18.66 Hz, Si-C***H₃***); 0.76 (9H, s, Si-C(C***H₃***)₃)); 1.00 – 1.19 (12H, m, ((C***H₃***)₂-CH)₂-N); 2.16 -2.76 (2H, m, OCH₂C***H₂***CN); 3.15-3.24 (1H, m, ***H***(a)-C(5')); 3.35 (2H, m, -OCH₂C***H₂*** (NPE); 3.50 – 3.61 (4H, m, ((CH₃)₂-C***H***)₂-N & ***H***(b)-C(5') & C***H₂***(b)-O-P)); 3.77 (6H, s, 2xO-C***H₃***); 4.27 – 4.42 (2H, m, ***H***-C(4') & ***H***-C(3')); 4.93 (2H, m, -OC***H₂***CH₂ (NPE)); 4.93 – 5.05 (3H, m, ***H***-C(2')); 5.92-6.10 (1H, dxd, J=23.85 Hz, ***H***-C(1')); 6.79 -7.55 (15H, m, C***H***-arom- DMT & o-***H***-phenyl (NPE); 7.75 (1H, d, J= 6.24Hz, ***H***-C(1)); 8.13-8.20 (3H, m, o-***H***-phenyl (NPE) & ***H***-C(8)); 8.67 (1H, b, -N***H***). ^{31}P-NMR: (162 MHz, $CDCl_3$, 25 °C)= 150.68 & 149.19 ppm.

^{1}H-NMR (100 MHz, CDCl3, 25 °C) of compound **12**

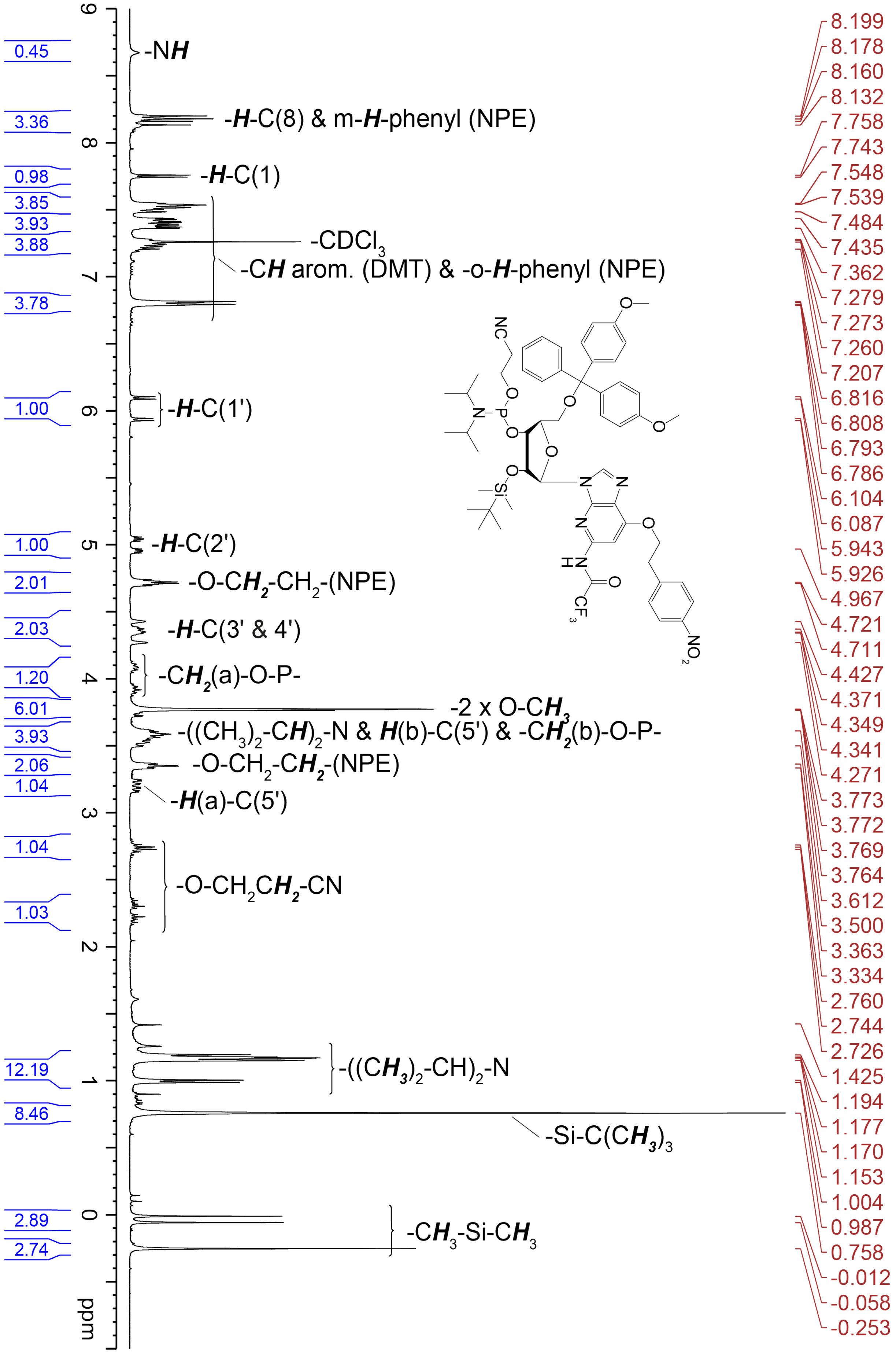

^{31}P-NMR (162 MHz, $CDCl_3$, 25 °C) of compound **12**

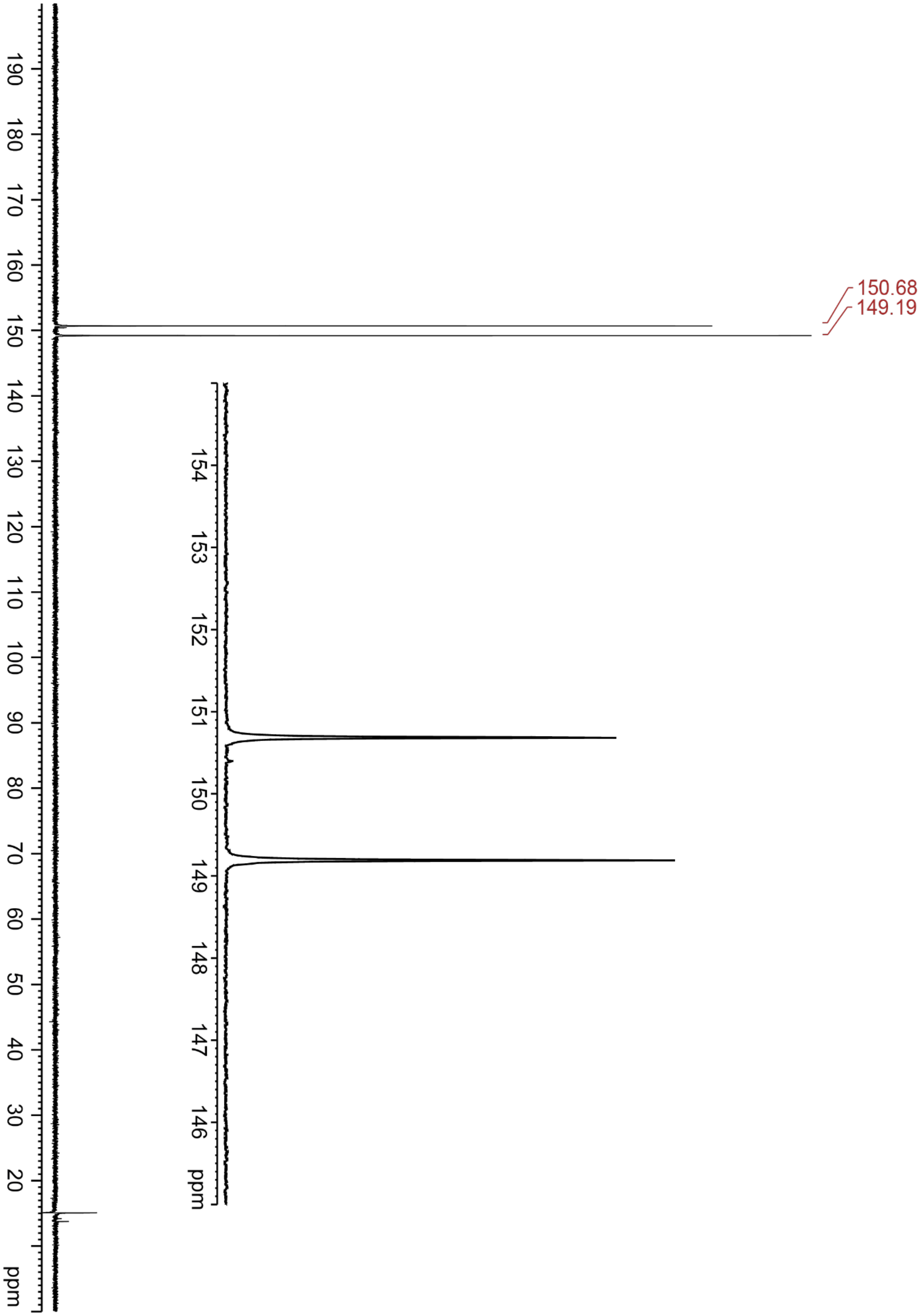

Supporting Table 1. Mass analysis of unmodified and modified RNAs used in this study.

Sequence (5' → 3')	nt	Molecular weight	
		calc.	found
GGUCGACC (**III**)	8	2524.59	2524.59
GGUCc^{1}GACC (**IIIa**)	8	2523.44	2523.77
GGCUAGCC (**III'**)	8	2524.59	2524.82
GGCUAc^{1}GCC (**III'a**)	8	2523.44	2523.47
GGCAGAGGC (**I**)	9	2932.87	2932.88
GCCUCUGCC (**Ia**)	9	2766.70	2766.88
GCCUUUGCC (**Ib**)	9	2767.69	2767.60
GCCUGUGCC (**Ic**)	9	2806.73	2806.52
GCCUAUGCC (**Id**)	9	2790.73	2790.61
GGCAc^{1}GAGGC (**I**)	9	2931.88	2932.02
GAAGGGCAACCUUCG (**II**)	15	4813.99	4813.55
GAAc^{1}GGGCAACCUUCG (**IIa**)	15	4812.84	4813.22
GAAGGc^{1}GCAACCUUCG (**IIb**)	15	4812.84	4812.50
UUUUUAAUGAAGCCACAGG	19	6046.70	6046.94
UGCUCCUAc^{1}GUACGAGAGGACCGGAGUG	27	8726.18	8726.67
GGCAGAUUGAGCCUGGCAGCUCUCUGCC	28	8985.44	8986.10
GGCAGAUUc^{1}GAGCCUGGCAGCUCUCUGCC	28	8984.29	8984.62
CCUGUGAGGGUCCUAAGCCCCUAAUUCAGAAGGGAAA	37	11901.26	11901.02
CCUGUGAGGGUCCUAAGCCCCUAAUUCAc^{1}GAAGGGAAA	37	11900.11	11900.30

Supporting Table 2. X-ray data collection and crystallographic refinement statistics.

	c^1G2655-SRL
PDB ID	7QP2
Space group	$P4_3$
a (Å)	29.48
b (Å)	29.48
c (Å)	76.49
Beamline	SLS PX III - X06DA
Resolution range (Å)	50 – 0.90
Number of frames	5400
Oscillation angle	0.2°
Wavelength	0.8
Average redundancy	37.9
Completeness [1]	100% (99.6%)
$CC_{1/2}$ [1]	100% (40.2%)
Average I/σ [1]	35.3 (1.2)
ISa0	22
R/R_{free}	11.7 / 13.8
Coordinate error (Å)	0.09
Wilson B (Å²)	9.5
Number of molecules	
RNA	1
Water	151
Glycerol	1

[1] Values for last resolution shell are shown in parenthesis

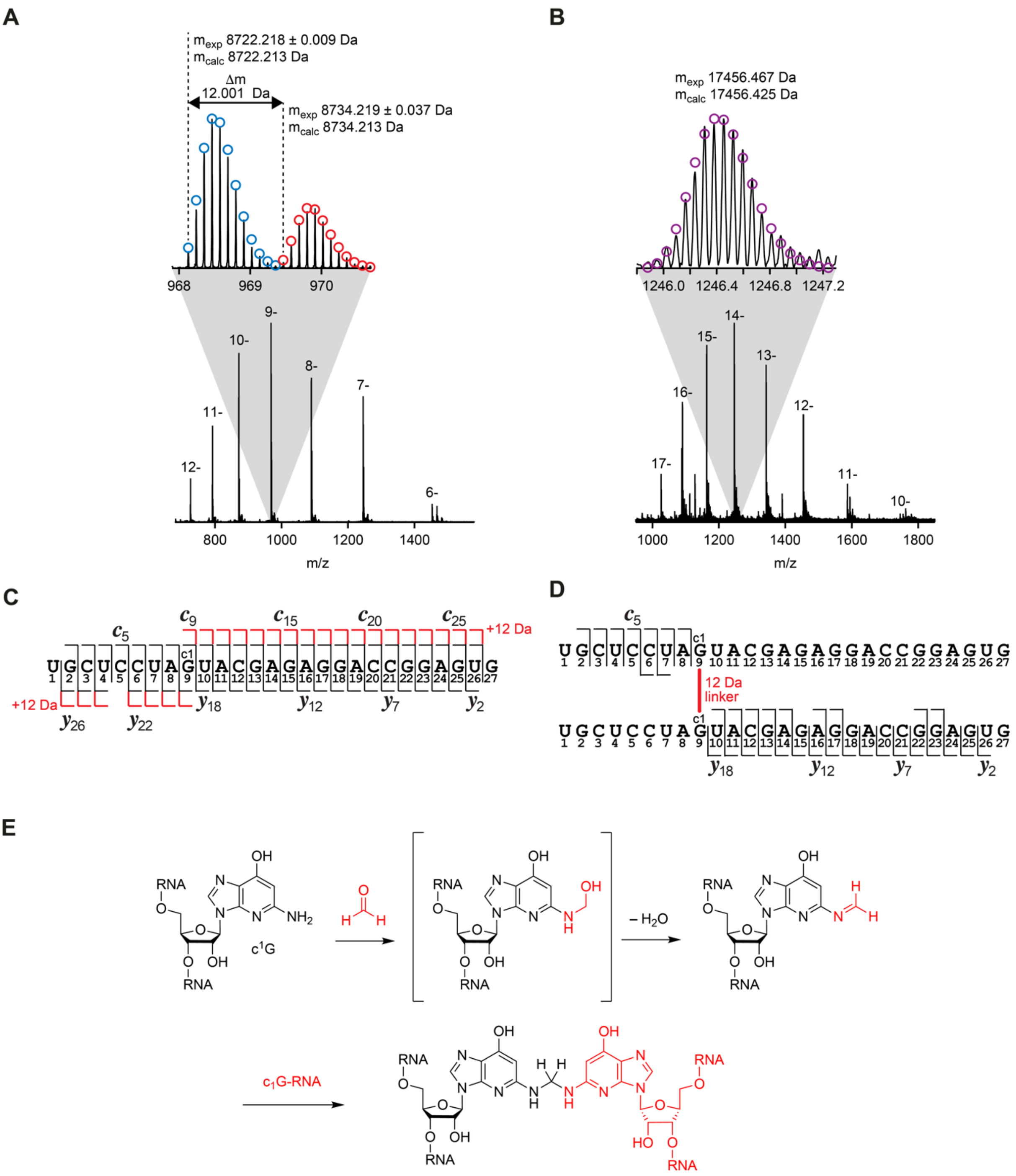

Supporting Figure 1. FT-ICR mass spectrometry of the *E. coli* sarcin/ricin stem-loop (SRL) RNA. **A**) The ESI mass spectra show excellent agreement between experimental (mexp 8722.218 ± 0.009 Da) and calculated (m_{calc} 8722.213 Da for $C_{260}H_{321}N_{107}O_{186}P_{26}$) monoisotopic mass, and reveal that approximately 25% of the monomeric RNA (peak 1 with shoulder in the chromatogram) had a mass value of 8734.219 ± 0.037 Da, which we attribute to reaction with formaldehyde (+12 Da,$C_{261}H_{321}N_{107}O_{186}P_{26}$). **B**) ESI MS of peak 2 in the chromatogram provides evidence for dimer formation between $C_{260}H_{321}N_{107}O_{186}P_{26}$ and $C_{261}H_{321}N_{107}O_{186}P_{26}$ (m_{exp} 17456.467 Da, m_{calc} 17456.425 Da). **C**) The sequence of the monomer was confirmed by top-down MS using collisionally activated dissociation (CAD) [see e.g. Flemmich, L. et al., *Nat. Commun*. **2021**, *12*, 3877.] and located the additional 12 Da to c^1G. **D**) Top-down MS of the dimer species produced fragments consistent with a formaldehyde crosslink between the two c^1G bases of each monomer. **E**) Putative mechanism and structure of the crosslinked c^1G-RNA dimer.

I

5' GC 3'
GC
CG
AU
GC
AU
GC
GC
3' CG 5'

concentration / µM	T_m(duplex) / °C
1.8	61.3
4.8	64.0
11.6	66.6
35.2	70.6
88.6	72.2

ΔG^0 kcal mol^{-1}	ΔH^0 kcal mol^{-1}	ΔS^0 cal mol^{-1} K^{-1}
-16.5	-79.7	-212.1

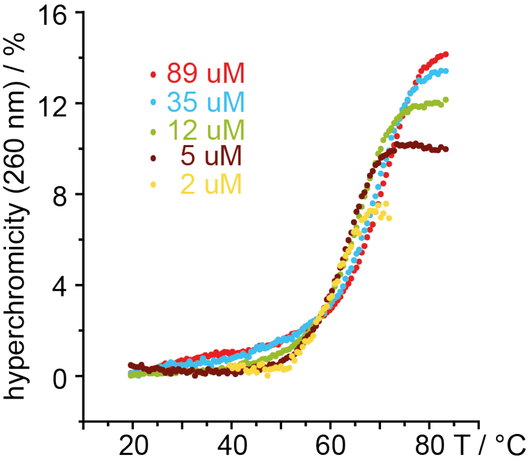

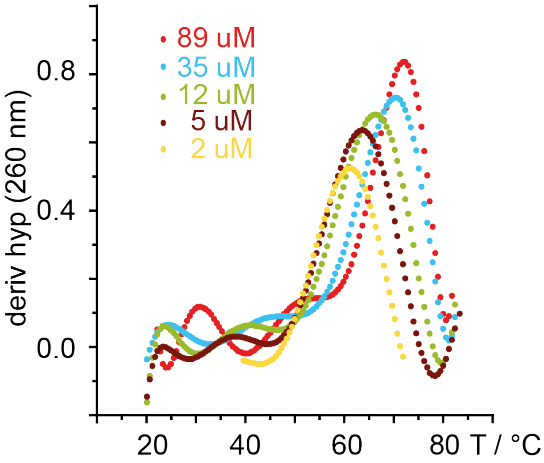

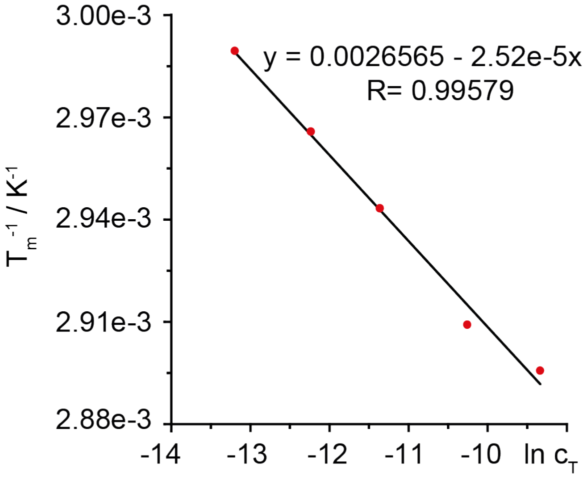

Supporting Figure 2. Thermodynamic analysis of RNA base pairing. Unmodified 9 bp duplex **I**: Sequence and secondary structure, summary of RNA concentrations, T_m values, and thermodynamic parameters (top left); graph illustrating the superposition of UV-melting profiles (top right), graph illustrating the superposition of the first derivatives of the melting curves (bottom left), and ln c versus 1/T plot (bottom right). Conditions: 10 mM Na_2HPO_4, 150 mM NaCl, pH 7.0.

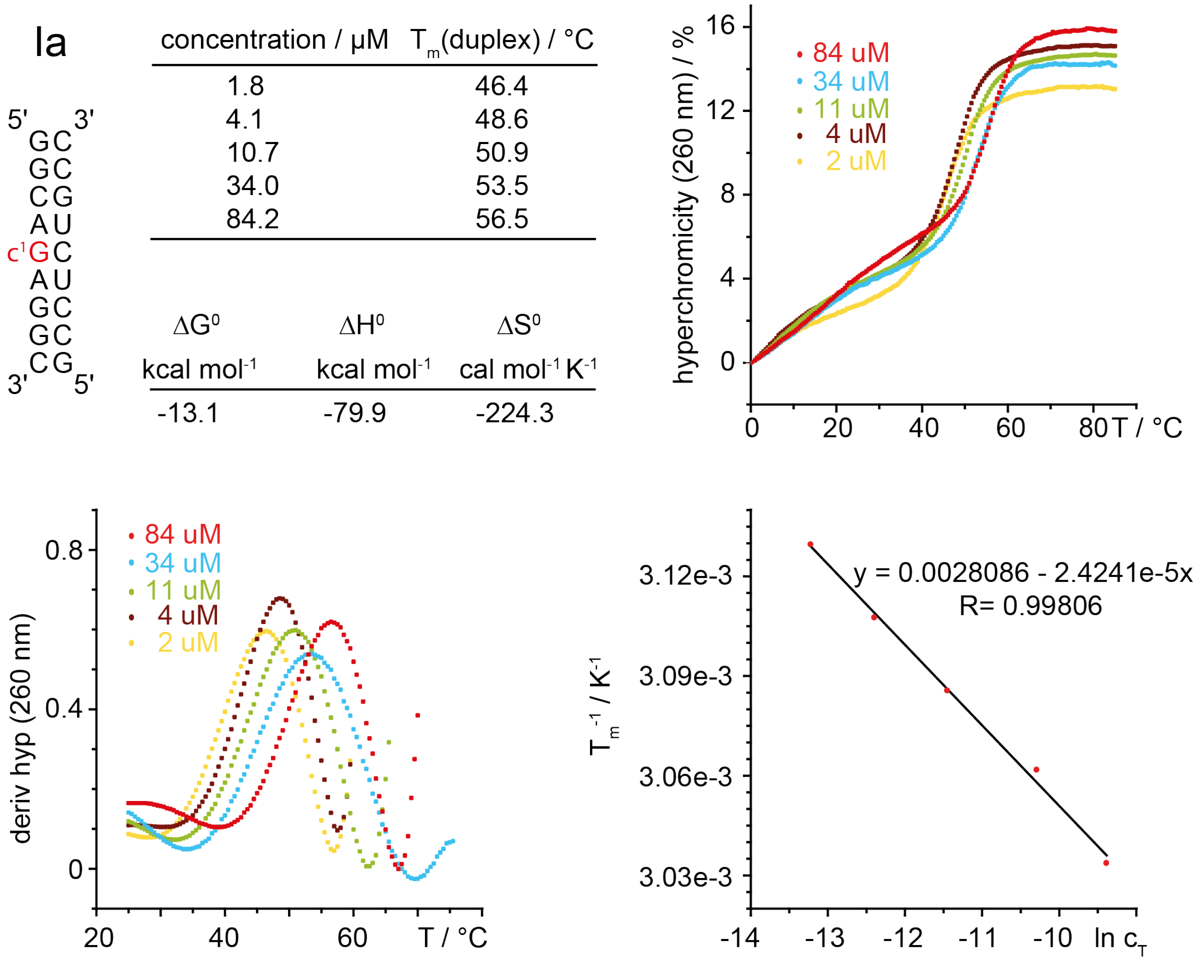

concentration / μM	T_m(duplex) / °C
1.8	46.4
4.1	48.6
10.7	50.9
34.0	53.5
84.2	56.5

ΔG^0 kcal mol^{-1}	ΔH^0 kcal mol^{-1}	ΔS^0 cal mol^{-1} K^{-1}
-13.1	-79.9	-224.3

Supporting Figure 3. Thermodynamic analysis of RNA base pairing. c^1G-C modified 9 bp duplex **Ia**: Sequence and secondary structure, summary of RNA concentrations, T_m values, and thermodynamic parameters (top left); graph illustrating the superposition of UV-melting profiles (top right), graph illustrating the superposition of the first derivatives of the melting curves (bottom left), and ln c versus 1/T plot (bottom right). Conditions: 10 mM Na_2HPO_4, 150 mM NaCl, pH 7.0.

Ib

5' 3'
GC
GC
CG
AU
c^1GU
AU
GC
GC
CG
3' 5'

concentration / µM	T_m(duplex) / °C
1.4	39.1
4.2	42.0
10.4	44.5
35.5	47.9
88.5	50.3

ΔG^0 kcal mol^{-1}	ΔH^0 kcal mol^{-1}	ΔS^0 cal mol^{-1} K^{-1}
-11.4	-73.6	-208.8

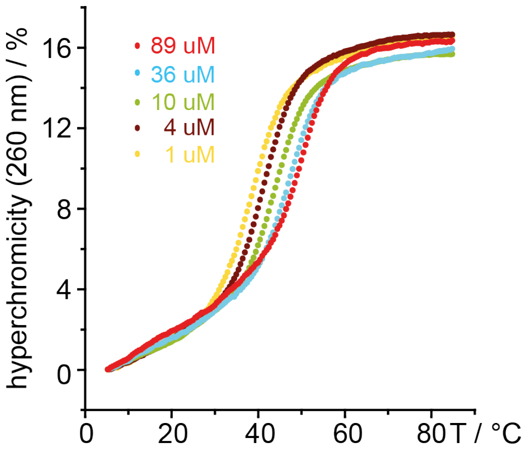

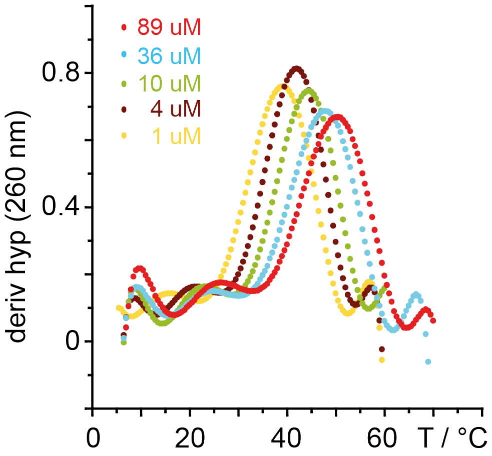

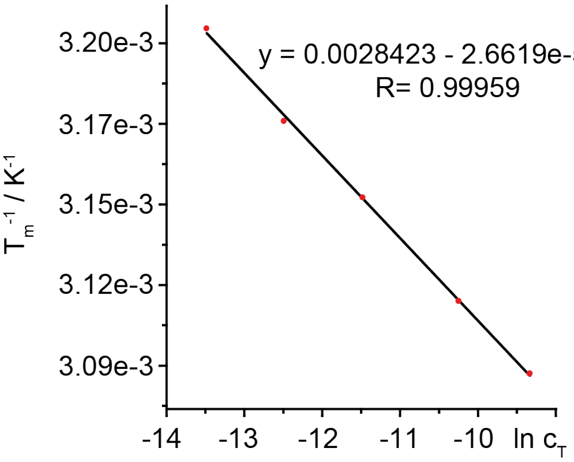

Supporting Figure 4. Thermodynamic analysis of RNA base pairing. c^1G-U modified 9 bp duplex **Ib**: Sequence and secondary structure, summary of RNA concentrations, T_m values, and thermodynamic parameters (top left); graph illustrating the superposition of UV-melting profiles (top right), graph illustrating the superposition of the first derivatives of the melting curves (bottom left), and ln c versus 1/T plot (bottom right). Conditions: 10 mM Na_2HPO_4, 150 mM NaCl, pH 7.0.

Ic

5' GC 3'
GC
GC
CG
AU
c1GG
AU
GC
GC
3' CG 5'

concentration / µM	T_m(duplex) / °C
1.7	38.0
4.6	41.0
12.2	43.3
36.8	47.0
91.3	48.9

ΔG^0 kcal mol^{-1}	ΔH^0 kcal mol^{-1}	ΔS^0 cal mol^{-1} K^{-1}
-10.9	-70.9	-201.5

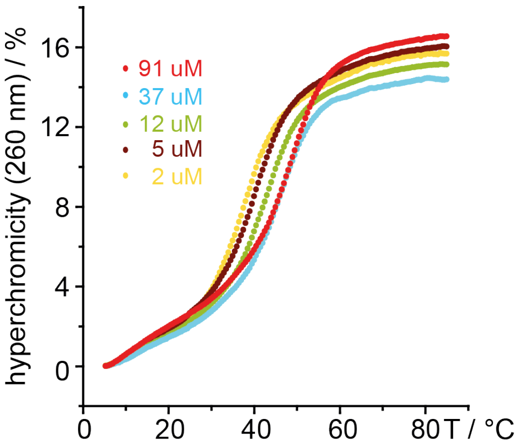

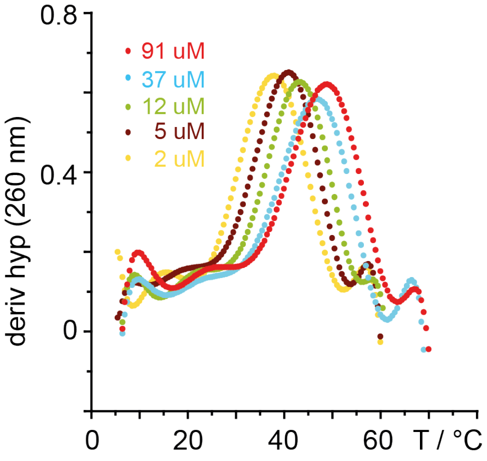

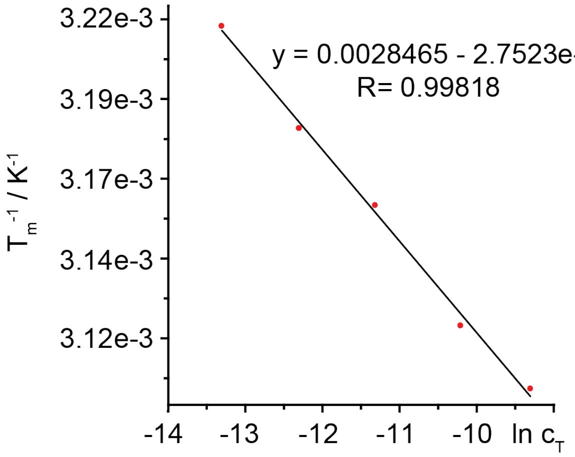

Supporting Figure 5. Thermodynamic analysis of RNA base pairing. c^{1}G-G modified 9 bp duplex **Ic**: Sequence and secondary structure, summary of RNA concentrations, T_m values, and thermodynamic parameters (top left); graph illustrating the superposition of UV-melting profiles (top right), graph illustrating the superposition of the first derivatives of the melting curves (bottom left), and ln c versus 1/T plot (bottom right). Conditions: 10 mM Na_2HPO_4, 150 mM NaCl, pH 7.0.

Id

```
5'   3'
  GC
  GC
  CG
  AU
c1G A
  AU
  GC
  GC
  CG
3'   5'
```

concentration / μM	T_m(duplex) / °C
1.8	35.2
4.8	38.4
12.4	40.7
36.7	43.5
93.9	46.5

ΔG^0 kcal mol^{-1}	ΔH^0 kcal mol^{-1}	ΔS^0 cal mol^{-1} K^{-1}
-10.2	-69.7	-199.6

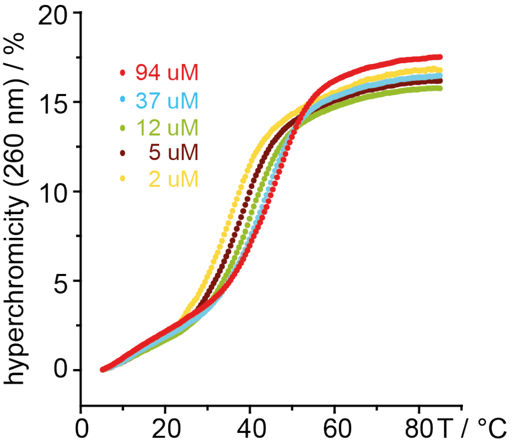

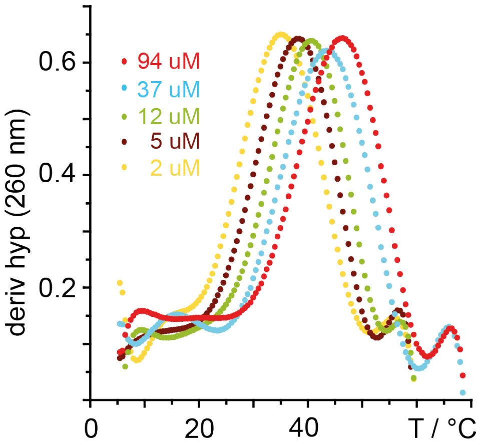

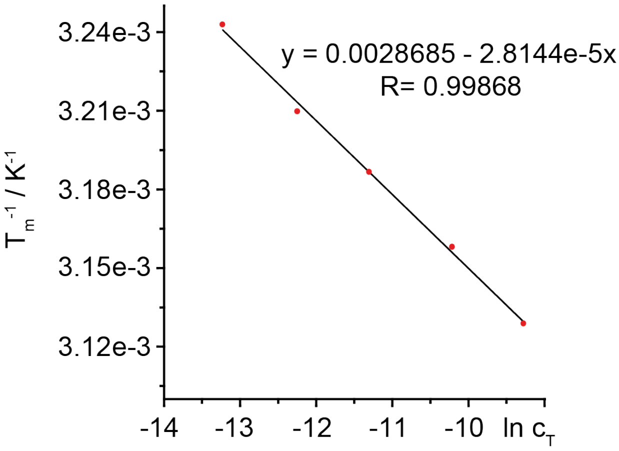

Supporting Figure 6. Thermodynamic analysis of RNA base pairing. c^1G-A modified 9 bp duplex **Id**: Sequence and secondary structure, summary of RNA concentrations, T_m values, and thermodynamic parameters (top left); graph illustrating the superposition of UV-melting profiles (top right), graph illustrating the superposition of the first derivatives of the melting curves (bottom left), and ln c versus 1/T plot (bottom right). Conditions: 10 mM Na_2HPO_4, 150 mM NaCl, pH 7.0.

III

5' G C 3'
G C
U A
C G
G C
A U
C G
C G
3' 5'

concentration / µM	T_m(duplex) / °C
2.4	53.2
5.2	55.6
12.5	58.2
40.8	62.4
99.9	65.4

ΔG^0 kcal mol^{-1}	ΔH^0 kcal mol^{-1}	ΔS^0 cal mol^{-1} K^{-1}
-13.2	-64.6	-172.2

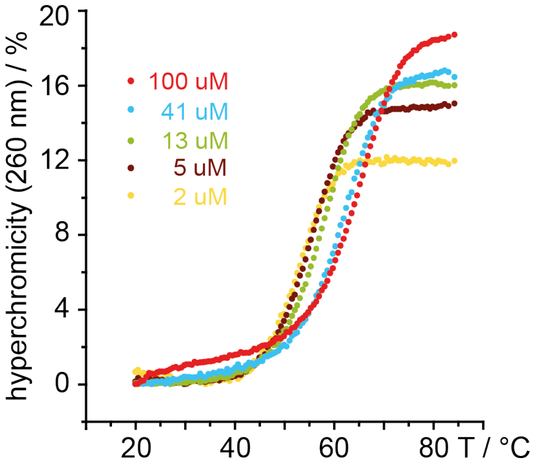

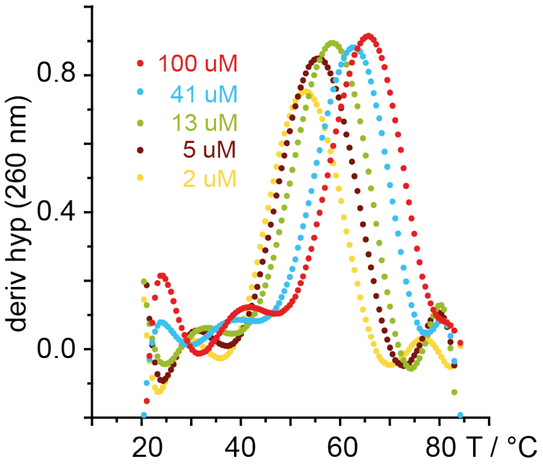

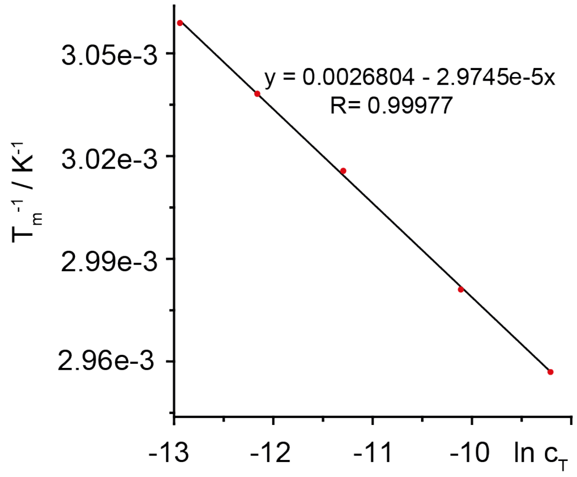

Supporting Figure 7. Thermodynamic analysis of RNA base pairing. Non-modified 8 nt oligoribonucleotide **III**: Sequence and secondary structure, summary of RNA concentrations, T_m values, and thermodynamic parameters (top left); graph illustrating the superposition of UV-melting profiles (top right), graph illustrating the superposition of the first derivatives of the melting curves (bottom left), and ln c versus 1/T plot (bottom right). Conditions: 10 mM Na_2HPO_4, 150 mM NaCl, pH 7.0.

IIIa

5' G C 3'
G C
U A
C c1G
c1G C
A U
C G
C G
3' 5'

concentration / µM	T_m(duplex) / °C
1.9	16.7
4.9	19.7
12.5	22.4
41.4	26.8
97.1	29.0

ΔG^0 kcal mol^{-1}	ΔH^0 kcal mol^{-1}	ΔS^0 cal mol^{-1} K^{-1}
-6.3	-55.0	-164.2

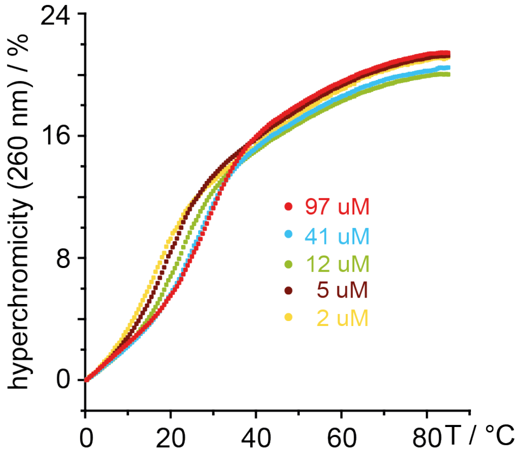

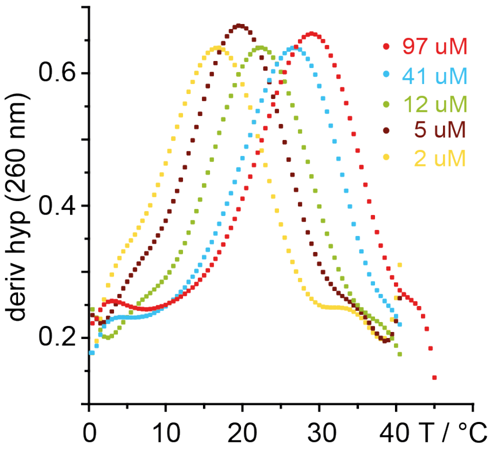

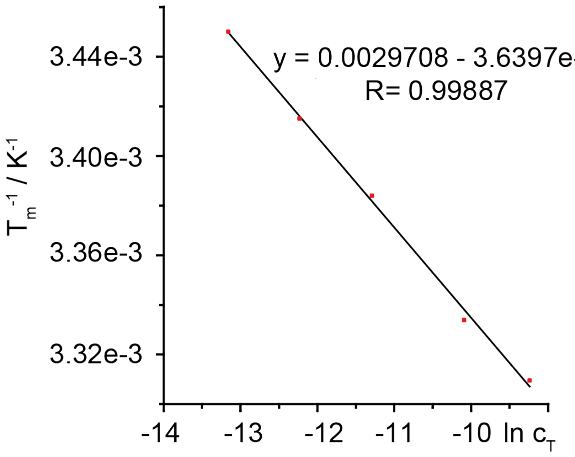

Supporting Figure 8. Thermodynamic analysis of RNA base pairing. c^{1}G-modified 8 nt oligoribonucleotide **IIIa**: Sequence and secondary structure, summary of RNA concentrations, T_m values, and thermodynamic parameters (top left); graph illustrating the superposition of UV-melting profiles (top right), graph illustrating the superposition of the first derivatives of the melting curves (bottom left), and ln c versus 1/T plot (bottom right). Conditions: 10 mM Na_2HPO_4, 150 mM NaCl, pH 7.0.

III'

5' G C 3'
G C
C G
U A
A U
G C
C G
C G
3' 5'

concentration / μM	T_m(duplex) / °C
2.3	55.9
4.7	58.0
10.7	60.3
33.2	63.8
65.0	65.9

ΔG^0 kcal mol^{-1}	ΔH^0 kcal mol^{-1}	ΔS^0 cal mol^{-1} K^{-1}
-14.5	-72.3	-194.2

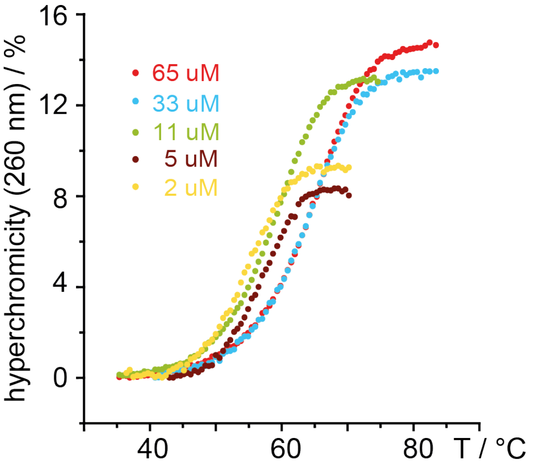

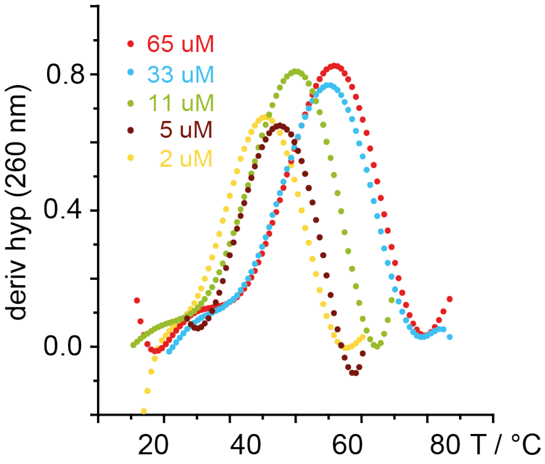

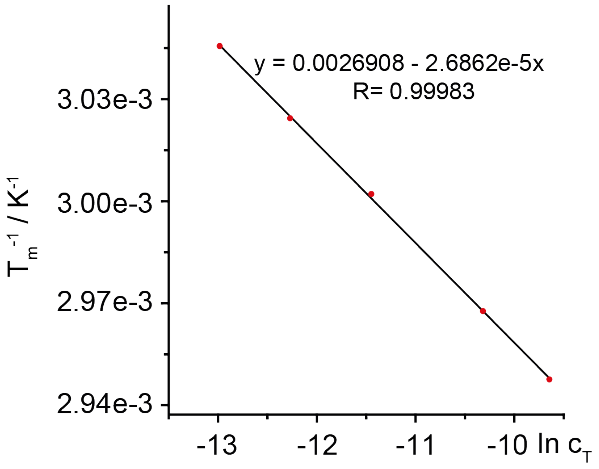

Supporting Figure 9. Thermodynamic analysis of RNA base pairing. Non-modified 8 nt oligoribonucleotide **III'**: Sequence and secondary structure, summary of RNA concentrations, T_m values, and thermodynamic parameters (top left); graph illustrating the superposition of UV-melting profiles (top right), graph illustrating the superposition of the first derivatives of the melting curves (bottom left), and ln c versus 1/T plot (bottom right). Conditions: 10 mM Na_2HPO_4, 150 mM NaCl, pH 7.0.

III'a

5' G C 3'
G C
C c1G
U A
A U
c1G C
C G
C G
3' 5'

concentration / µM	T_m(duplex) / °C
2.0	18.7
4.9	21.5
12.7	24.4
36.7	27.0
81.6	30.3

ΔG^0 kcal mol^{-1}	ΔH^0 kcal mol^{-1}	ΔS^0 cal mol^{-1} K^{-1}
-6.6	-58.4	-174.4

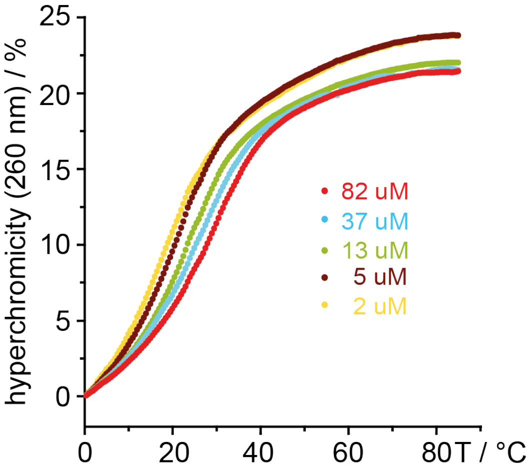

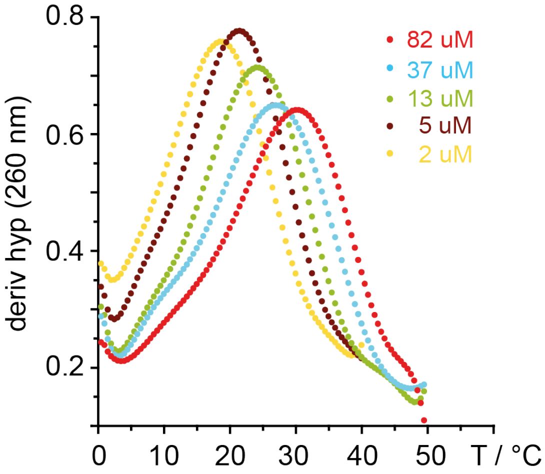

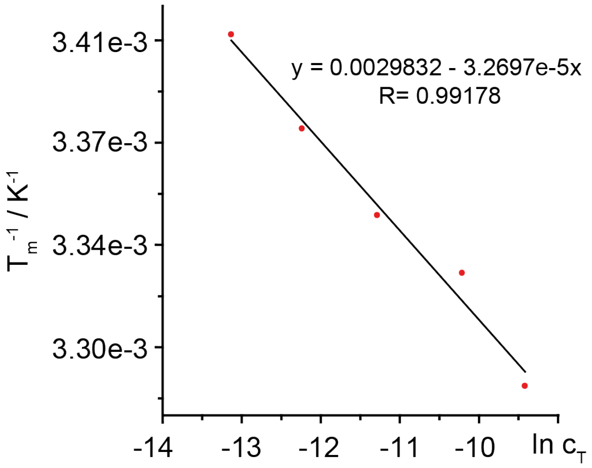

Supporting Figure 10. Thermodynamic analysis of RNA base pairing. c^{1}G-modified 8 nt oligoribonucleotide **III'a**: Sequence and secondary structure, summary of RNA concentrations, T_m values, and thermodynamic parameters (top left); graph illustrating the superposition of UV-melting profiles (top right), graph illustrating the superposition of the first derivatives of the melting curves (bottom left), and ln c versus 1/T plot (bottom right). Conditions: 10 mM Na_2HPO_4, 150 mM NaCl, pH 7.0.

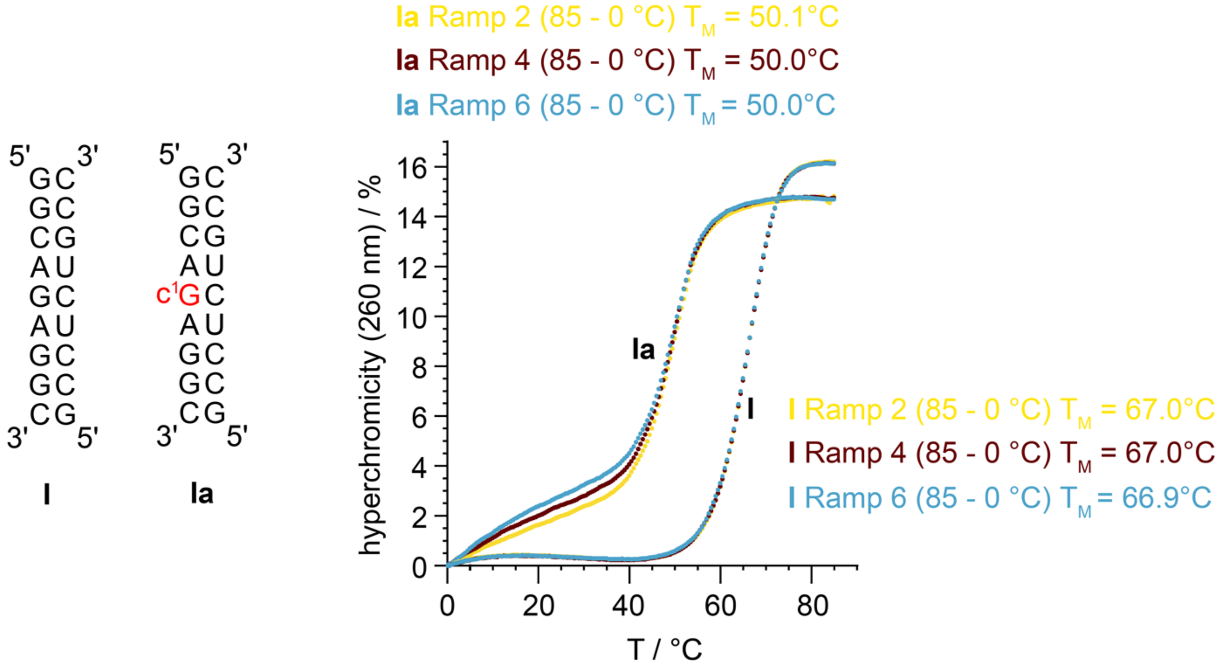

Supporting Figure 11. Thermodynamic analysis of RNA base pairing. Example showing the overlay of 3 ramps for two distinct RNAs providing evidence for retaining the integrity of RNA samples during extended experiment time. Conditions: c(RNA) = 10 µM, 10 mM Na_2HPO_4, 150 mM NaCl, pH 7.0.

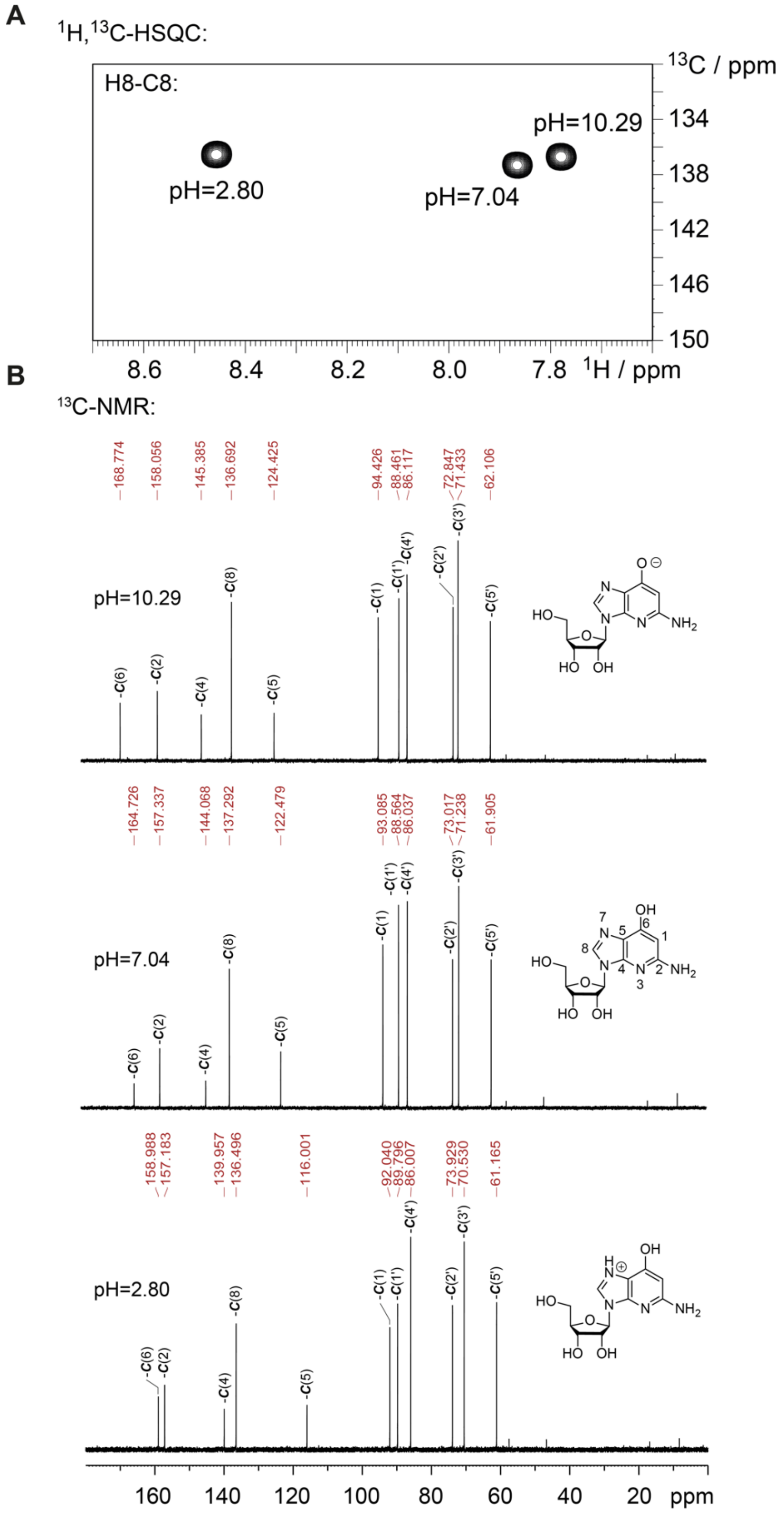

Supporting Figure 12. NMR spectroscopic experiments to assign the site of protonation of c[1]G; **A**) Overlay of ^{1}H,^{13}C HSQC spectra for the H8-C8 region, pH as indicated, and (**B**) ^{13}C NMR spectra, pH as indicated. Conditions: 3 mg c[1]G, in 0.5 mL H_2O, 5% D_2O. The ^{1}H chemical shift of H8 and the ^{13}C chemical shift of C5 suggested N7 as the site of protonation.

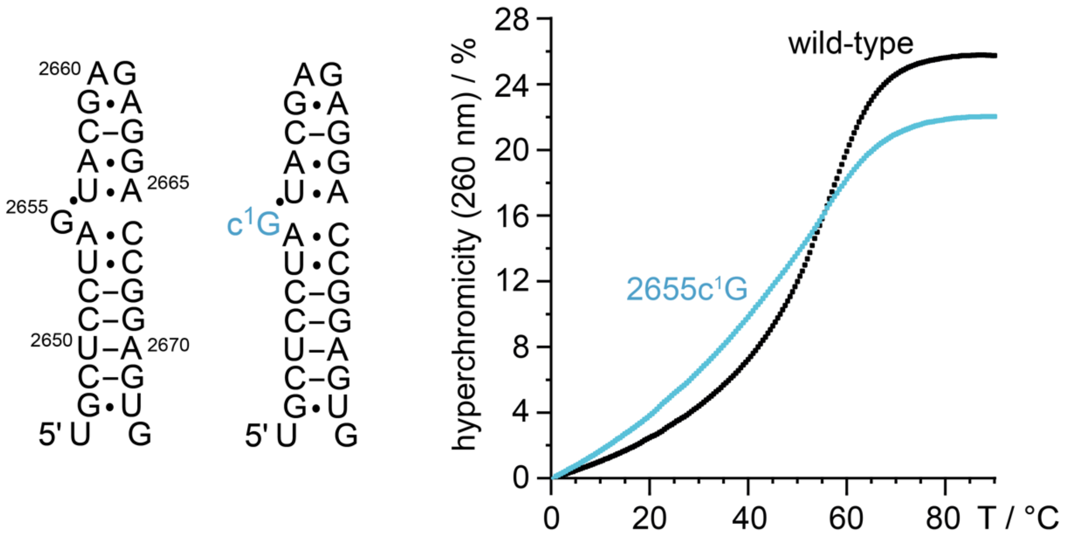

Supporting Figure 13. Melting curves of modified and unmodified *E. coli* sarcin/ricin stem-loop (SRL). Conditions: c(RNA) = 5 µM, 10 mM Na_2HPO_4, 150 mM NaCl, pH 7.0.

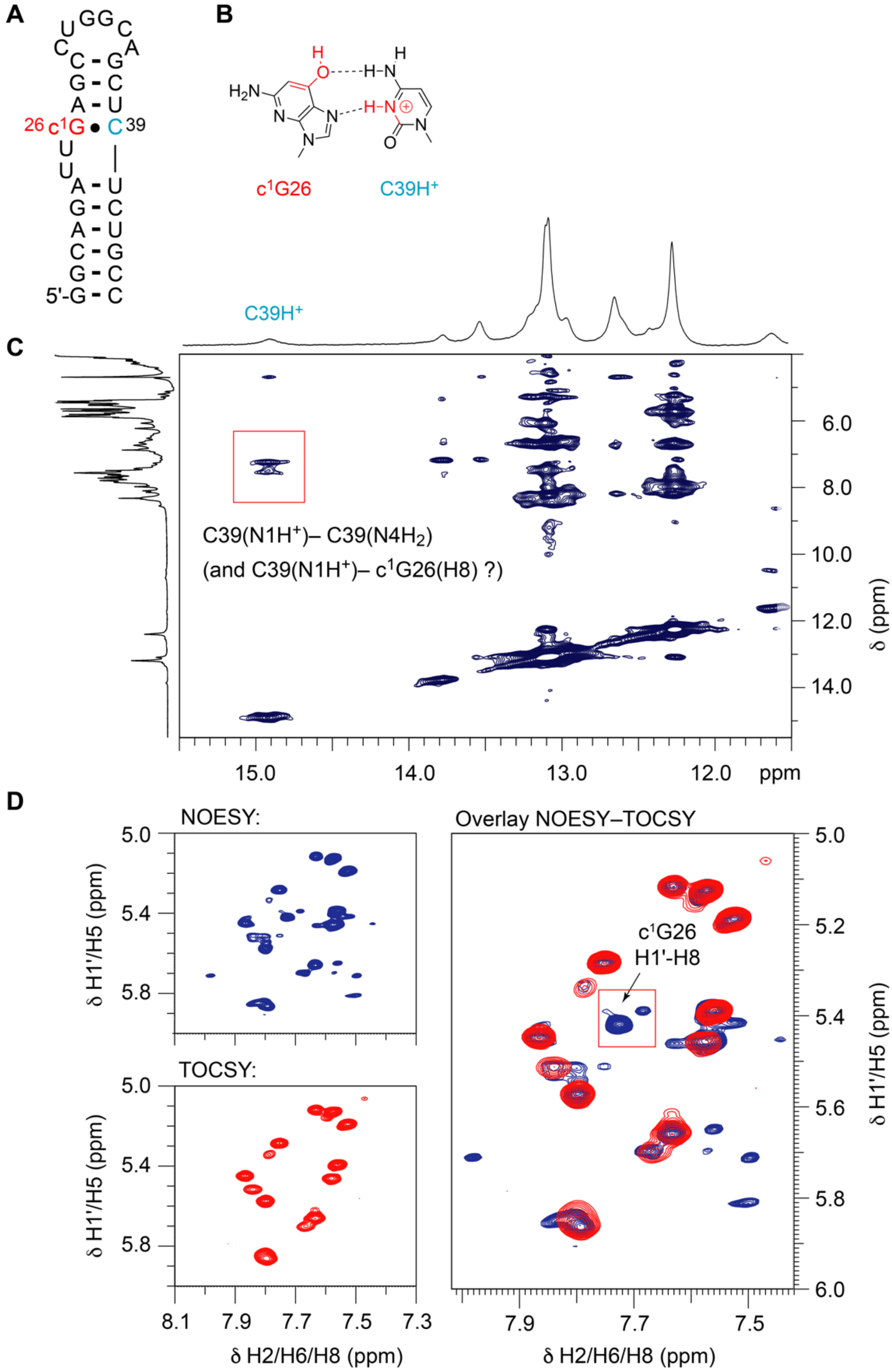

Supporting Figure 14. NMR spectroscopy of the 28nt c[1]G modified HIV-2-TAR RNA. **A**) Sequence and secondary structure; **B**) Hoogsteen base pair of c[1]G-C; **C**) ^{1}H/^{1}H-NOESY of the imino and imino/H2/H6/H8/amino region and tentative assignment. **D**) ^{1}H/^{1}H-NOESY of the H1'/H5 and H2/H6/H8 region (top left), ^{1}H/^{1}H-TOCSY of the H1'/H5 and H2/H6/H8 region (bottom left), **o**verlay of the NOESY and TOCSY spectra (right). Conditions: 25 mM NaCl, 10% D_2O, pH=5.4 at 25°C. For assignment comparison to m[1]G26 modified HIV-2-TAR RNA see A. Rangadurai, H. Zhou, D. K. Merriman, N. Meiser, B. Liu, H. Shi, E. S. Szymanski, H. M. Al-Hashimi, *Nucleic Acids Res*. **2018**, *46*, 11099–11114.

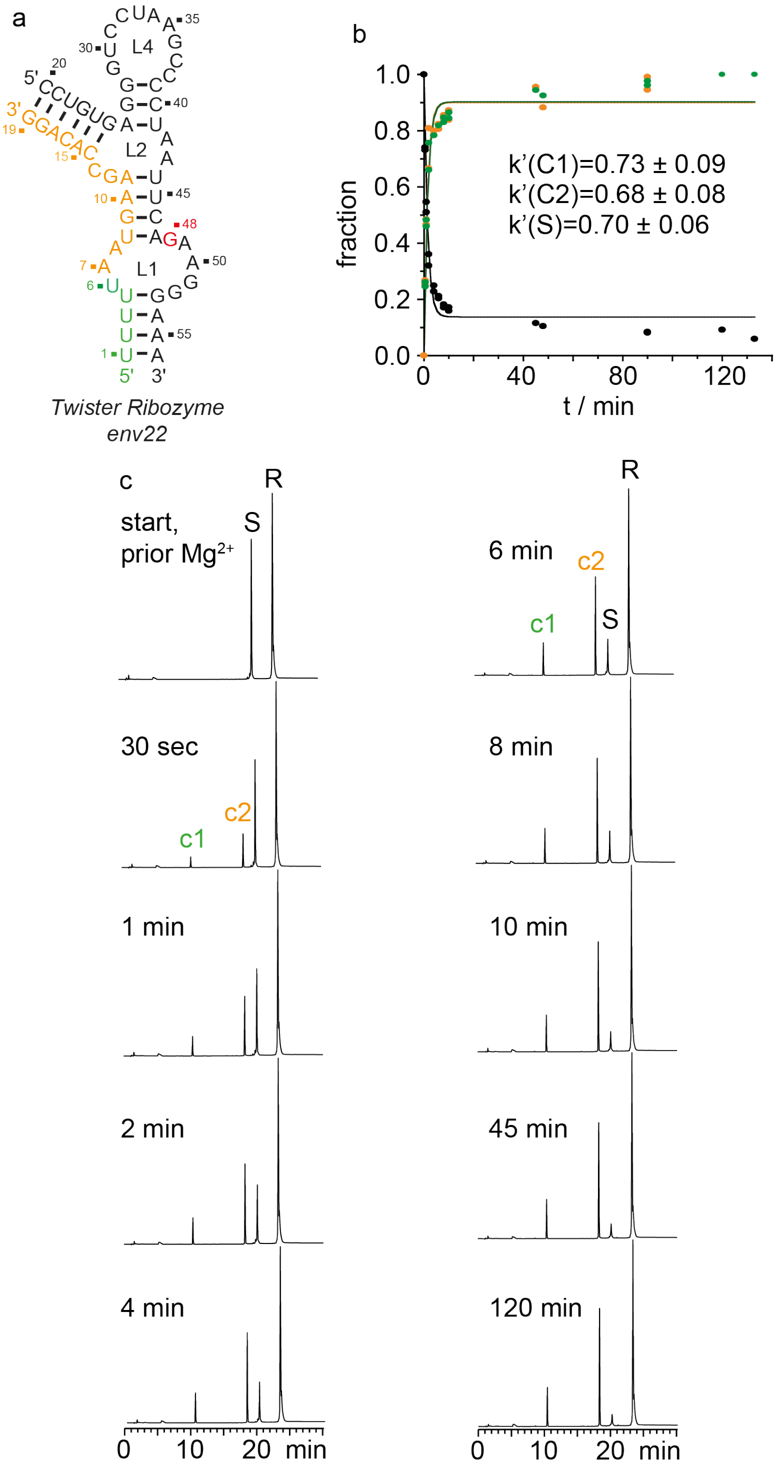

Supporting Figure 15. Self-cleavage of the *env22* twister ribozyme. **A**) Sequence and secondary structure; **B**) Estimation of observed rate from fractions (S, C1, and C2) obtained by HPLC analysis **C**) Anion exchange HPLC traces of the reaction time course; reaction conditions: c(RNA) = 55 µM each RNA strand (1:1 ratio); 2 mM $MgCl_2$, 100 mM KCl, 30 mM HEPES, pH 7.5, 23 °C. The reaction was stopped at the indicated time points by drawing a 4 µL sample and mixing it with 4 µL of 40 mM Na_2EDTA, followed by dilution to 100 µL of water. HPLC conditions: Dionex DNAPac column (4x250 mm), 60 °C, 1 ml min^{-1} , 0–60% buffer B in 45 min. Buffer A: Tris–HCl (25 mM), $NaClO_4$ (10 mM), 20% acetonitrile, pH 8.0. Buffer B: Tris–HCl (25 mM), $NaClO_4$ (600 mM), 20% acetonitrile, pH 8.0.

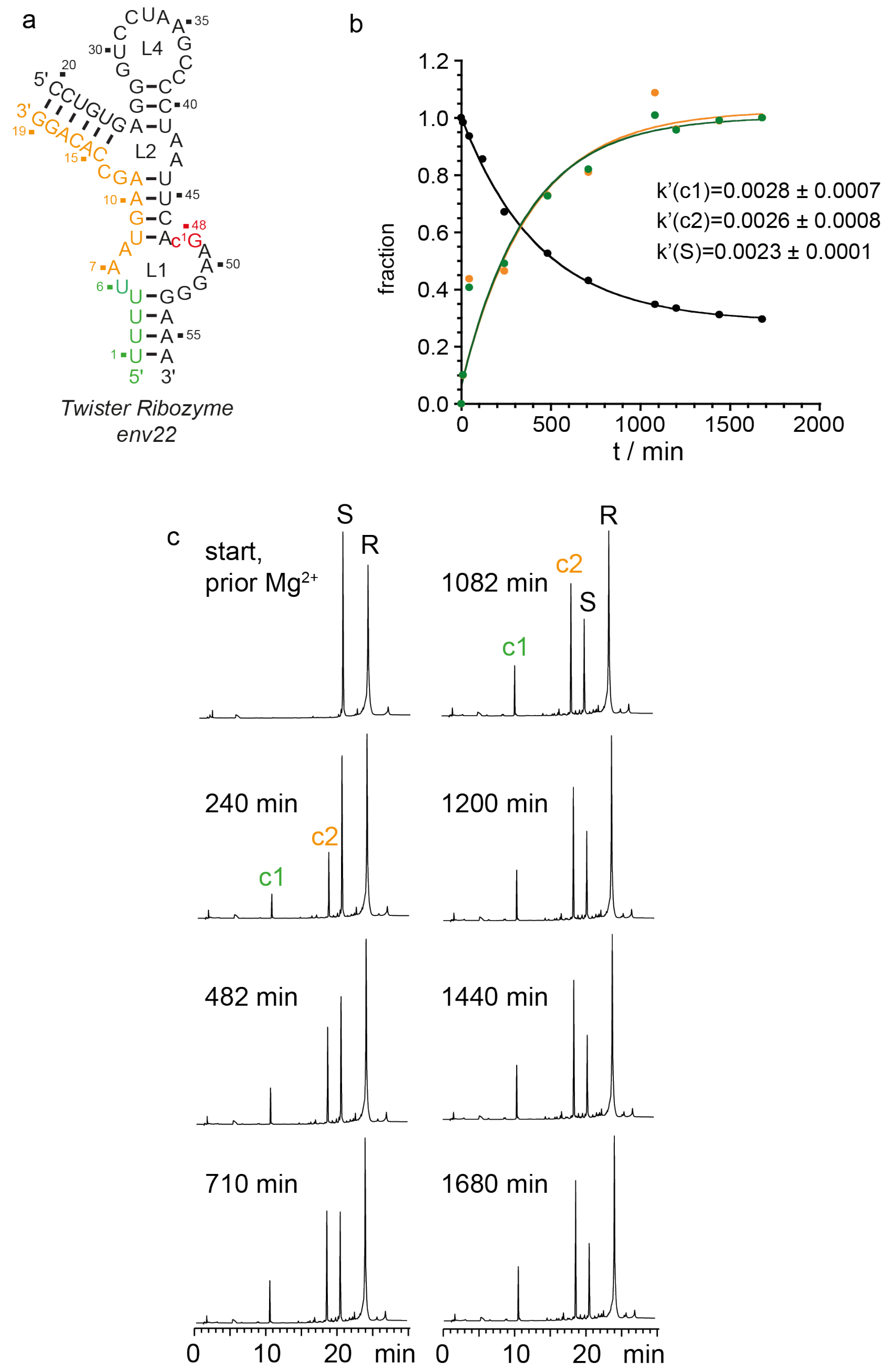

Supporting Figure 16. Self-cleavage of the twister G48c[1]G mutant. **A**) Sequence and secondary structure; **B**) Estimation of observed rate from fractions (S, C1, and C2) obtained by HPLC analysis **C**) Anion exchange HPLC traces of the reaction time course; reaction conditions: c(RNA) = 55 µM each RNA strand (1:1 ratio); 2 mM $MgCl_2$, 100 mM KCl, 30 mM HEPES, pH 7.5, 23 °C. The reaction was stopped at the indicated time points by drawing a 4 µL sample and mixing it with 4 µL of 40 mM Na_2EDTA, followed by dilution to 100 µL of water. HPLC conditions: Dionex DNAPac column (4x250 mm), 60 °C, 1 ml min^{-1} , 0–60% buffer B in 45 min. Buffer A: Tris–HCl (25 mM), $NaClO_4$ (10 mM), 20% acetonitrile, pH 8.0. Buffer B: Tris–HCl (25 mM), $NaClO_4$ (600 mM), 20% acetonitrile, pH 8.0.
